# Supplementary material for: A risk-adjusted and anatomically stratified cohort comparison study of open surgery, endovascular techniques and medical management for juxtarenal aortic aneurysms—the UK COMPlex AneurySm Study (UK-COMPASS): a study protocol
Source: BMJ Open. 2021 Nov 30;11(11):e054493. doi: 10.1136/bmjopen-2021-054493 (PMC8634354; doi:10.1136/bmjopen-2021-054493)
Supplement: Supplementary data [file bmjopen-2021-054493supp001.pdf]

Online Supplementary Material for UK-COMPASS protocol

Contents

Section	Content	Pages
Section 1	The UK-COMPASS Corelab protocol – details on CT scan management, quality control, protocol development and clinical consensus decision-making.	2-3
Section 2	Definitions used in the Corelab protocol measurement process (Table)	4
Section 3	The UK-COMPASS Corelab protocol – the measurement process	5-13
Section 4	The UK-COMPASS Corelab protocol – internal validation of the measurement process.	14-16
Section 5	Microcosting exercise data collection template to be used in the site-recruited data stream.	17-23

Patel SR, *et al. BMJ Open* 2021; 11:e054493. doi: 10.1136/bmjopen-2021-054493

Section 1 - The UK-COMPASS Corelab protocol – details on Computerised Tomography (CT) scan management, quality control and protocol development.

UK-COMPASS's Corelab is a fully digital and paper-free facility in keeping with NIHR's carbon reduction guidelines.

CT scan management

CT scan management involves the retrieval, pseudonymisation and upload of CT scans for Corelab analysis in line with regulatory approvals. It is performed by the 'Research PACS Clerk' who is a trained member of the UK-COMPASS team and an employee of the host NHS trust. The process is conducted in compliance with the principles of The General Data Protection Regulation (GDPR), tailored by the Data Protection Act 2018, with appropriate training for all members of the study team.

The date and location of the most recent CT scan of the abdominal aorta, prior to the date of the patient's operation, is identified from Hospital Episodes Statistics (administrative data warehouse containing details of all admissions, outpatient appointments and emergency department attendances at NHS hospitals in England). This CT scan Digital Imaging and Communications in Medicine (DICOM) data is retrieved via the NHS Picture Archiving Communication System (PACS) utilising NHS number, date of birth and the date of specific CT scan in question, as provided by NHS Digital under a data sharing agreement. Legal basis was secured to access patient data without consent through the Secretary of State's Section 251 approval with Confidentiality Advisory Group (CAG) recommendation. Upon receipt, the scan data is irreversibly pseudonymised. This process was initially carried out using Carestream Vue PACS v11.4.1.1011 software (Carestream Health, Rochester, NY, USA), but later Osirix MD (Osirix, Geneva, Switzerland) was used for faster processing. This results in removal of all identifiers from the DICOM dataset such as name, date of birth, NHS and local hospital ID numbers. These data are replaced with a unique Study ID number which acts as pseudonym. It is not possible for the study team to re-identify any patient from this point.

The pseudonymised scans are uploaded onto a research section of the local PACS server, entitled the "Vendor Neutral Archive" (VNA), accessible via a two-stage authentication process and only to members of the UK-COMPASS team approved for Corelab analysis. Analysis of these scans is therefore blinded. It is possible to see the Study ID and date of scan acquisition when a CT is loaded for analysis, but not possible to re-identify a patient.

Quality Control of Management process

The Research PACS Clerk removes identifiable data from a retrieved CT scan using the PACS computer programme. Successful completion of pseudonymisation is verified for each scan in a 3-step quality control process. **Step 1:** The PACS clerk will perform complete anonymisation at DICOM data level upon receipt of the CT scan. **Step 2**: CT image set placed into an upload portal and success of anonymisation is verified. **Step 3:** The PACS clerk will retrieve the CT image set from the VNA into the Carestream viewing client and conduct a final check.

As of June 2021, of the 8613 scans that had been measured in the Corelab, no scans were found to have failed anonymisation confirming robustness of the quality control process.

Development of measurement protocol

The Corelab protocol was initially devised by consensus between three researchers (Chief Investigator: SV and two clinical research fellows: SP and DO) following literature review regarding anatomical features of complexity with respect to the neck of abdominal aortic aneurysms. With paucity of information regarding Corelab set-up in the published literature, content was determined by consensus and subsequent approval by members of the UK-COMPASS Clinical Consensus Group.

Clinical consensus exercise

A Clinical Consensus Group made up of Vascular Surgeons and relevant multidisciplinary experts from the U.K. met (December 2019) to decide anatomical definitions and a priori grouping for analysis. Corelab procedures were provided in advance. Suggestions and discussion were undertaken in a focus group format with opportunity for all to express opinion before reaching consensus through voting.

It was decided that neck length is the morphological feature of most interest with regards to complex aneurysms. Grouping by neck length in mm would provide clinical relevance to the findings and limit anatomical heterogeneity. Furthermore, the degree to which an infrarenal neck is short will influence which specific endovascular techniques are likely to be employed as well as whether the aortic clamp during OSR can be placed below the renal arteries. Therefore, the following primary anatomical stratification was recommended:

Group 1: neck length 4mm or shorter
Group 2: neck length 5-9mm
Group 3: neck length ≥10mm AND unsuitable for standard EVAR within IFU

Within group 3, it is appreciated that there will be a variety of adverse neck features present other than neck length (conicality, angulation, thrombus burden and calcification). It may be possible to separate these depending on the numbers included but the a priori analysis plan will be to group these cases.

Large neck diameter is an adverse neck feature. It was felt that 30mm should be the distinction between normal aortic neck and aneurysmal aorta. Therefore, it is recognised that there may be cases of uniform necks with adequate length but large diameter (≥30mm) in Group 1. These can be removed and interrogated as a subgroup analysis. There will therefore be no large diameter necks in Group 3.

Section 2 - Definitions used in the Corelab protocol measurement process (Table)

Measurement terminology	Unit of measurement	Definition
**Total neck length**	Millimetres (mm)	Distance between:<br>a) Lowermost major renal artery and,<br><br>b) - Point of abrupt change in calibre *(if the neck is parallel sided and <30mm diameter)* OR,<br>- where the aorta reached 30mm in diameter
**Neck diameter**	Millimetres (mm)	Orthogonal diameter of infrarenal neck at specified distances (0mm, 5mm, 10mm and 15mm) below the origin of the lowermost major renal artery (outer wall to outer wall diameter)
**α neck angle**	Degrees (°)	Angle between axis of suprarenal aorta and axis of infrarenal neck
**β neck angle**	Degrees (°)	Angle between axis of infrarenal neck and axis of aneurysm
**Non-straight neck**	Binary	Presence of intra-neck angulation >60°
**Intra-neck angle** *(when non-straight neck present)*	Degrees (°)	Angle between juxtarenal neck and remainder of neck
**Juxtarenal neck length** *(when non-straight neck present)*	Millimetres (mm)	Distance between lowermost major renal artery and point of intra-neck angulation
**Aneurysm Diameter**	Millimetres (mm)	Maximum orthogonal diameter of aneurysm (outer-to-outer)
**Excessive thrombus**	Binary	Presence of thrombus lining >1/3 circumference of neck or filling >1/3 surface area of the axial slice, along a 3mm length of neck
**Excessive calcification**	Binary	Presence of calcium involving >1/3 circumference of neck in axial slices along a 3mm length of neck

Patel SR, *et al. BMJ Open* 2021; 11:e054493. doi: 10.1136/bmjopen-2021-054493

Section 3 - The UK-COMPASS Corelab protocol – the measurement process

Image analysis was initially performed using the multi-planar reconstruction function of Carestream Vue PACS v11.4.1.1011 software (Carestream Health, Rochester, NY, USA). After completion of measurement on approximately 3000 scans, the host site installed an updated version of the same programme (v12.2.2.1025) for the measurement process of the remainder. h

CT series of the abdominal aorta with arterial phase contrast were loaded in double-oblique multiplanar reconstructed (MPR) format. This permitted visualisation of the aneurysm/aneurysm neck in axial, sagittal and coronal views. Contrast windowing and magnification were considered baseline adjustments prior to commencement of the measurement process, which is detailed below for specific measures:

1. *Aneurysm diameter*

Both the coronal and sagittal planes are aligned with the long axis of the aneurysm body (not the flow lumen) using double oblique adjustment. This allows orthogonal axial view of the aneurysm at its largest point. A ruler function is used to measure the maximal (outer to outer) diameter of the aneurysm in the axial plane (Figures 1a and 1b).

1a

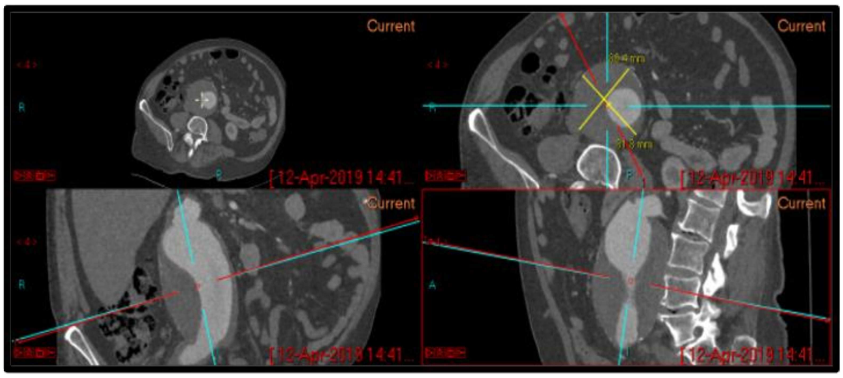

1b

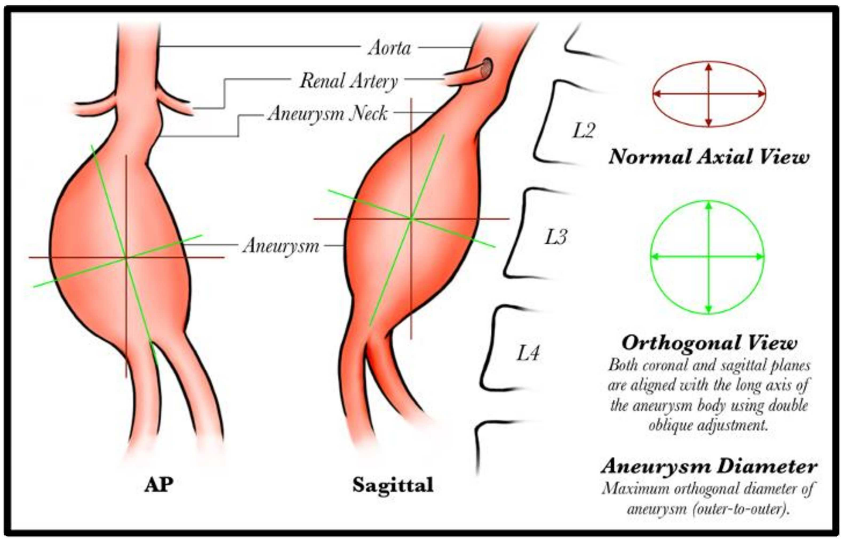

Patel SR, *et al. BMJ Open* 2021; 11:e054493. doi: 10.1136/bmjopen-2021-054493

2. *Total neck length*

Total neck length is defined as the distance between the inferior aspect of the lowermost main renal artery and the superior aspect of the aneurysm.
The superior aspect of the aneurysm is defined as:

- The transition between normal aorta and aneurysm, where the aneurysm commences abruptly (if the neck is parallel sided and <30mm diam), OR
- the point at which the neck diameter becomes 30mm in cases where the transition to aneurysm is not abrupt.

Long axis of the aneurysm neck is aligned to both coronal and sagittal planes and the distal margin of the lower most renal artery is profiled by aligning double-oblique planes to the renal artery ostium in the axial view. A ruler function is used to measure the neck length in both coronal and sagittal views (Figures 2a and 2b).

2a

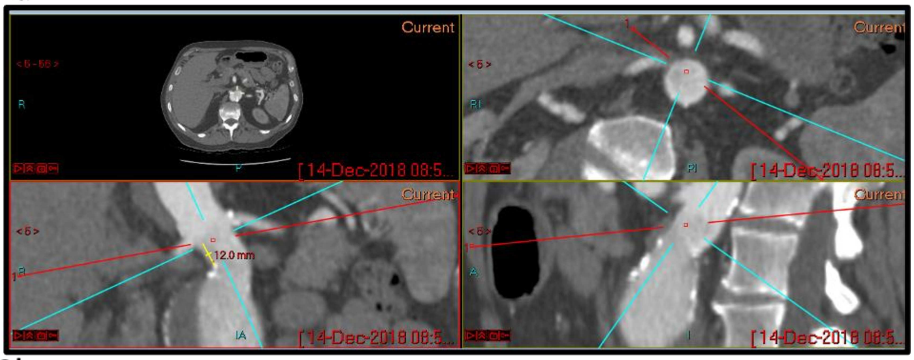

2b

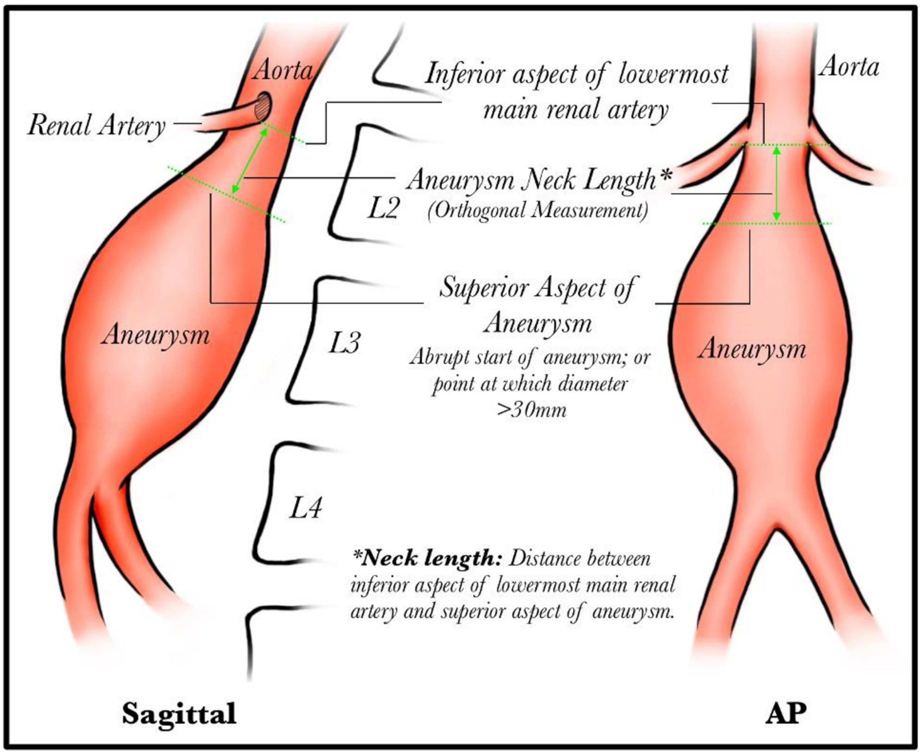

6

3. <u>β neck angle</u>

Beta (β) neck angle refers to the angle between the axis of the neck and the axis of the aneurysm, measured in degrees.

The first step is to align both coronal and sagittal planes to the axis of the aneurysm neck. Tilting the double-oblique marker lines in the axial view will permit visualisation of the aneurysm axis in the coronal or sagittal views. The largest possible angle between the axis of the neck and the axis of the aneurysm is measured and subtracted from 180 to calculate the β neck angle (Figures 3a and 3b).

3a

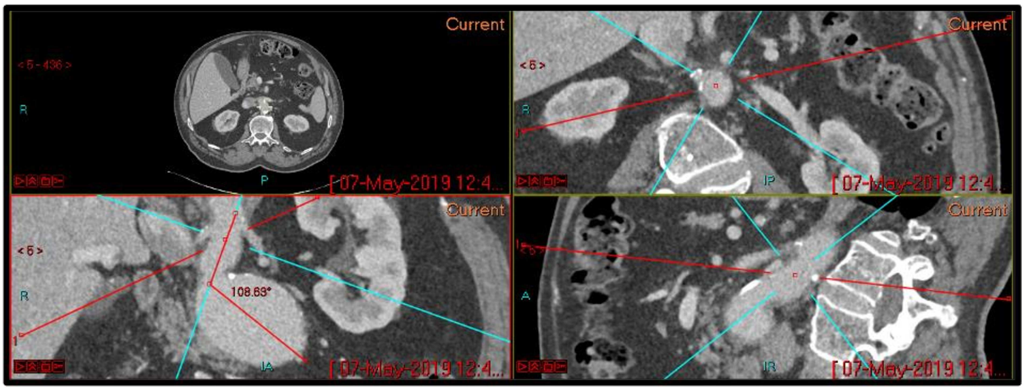

3b

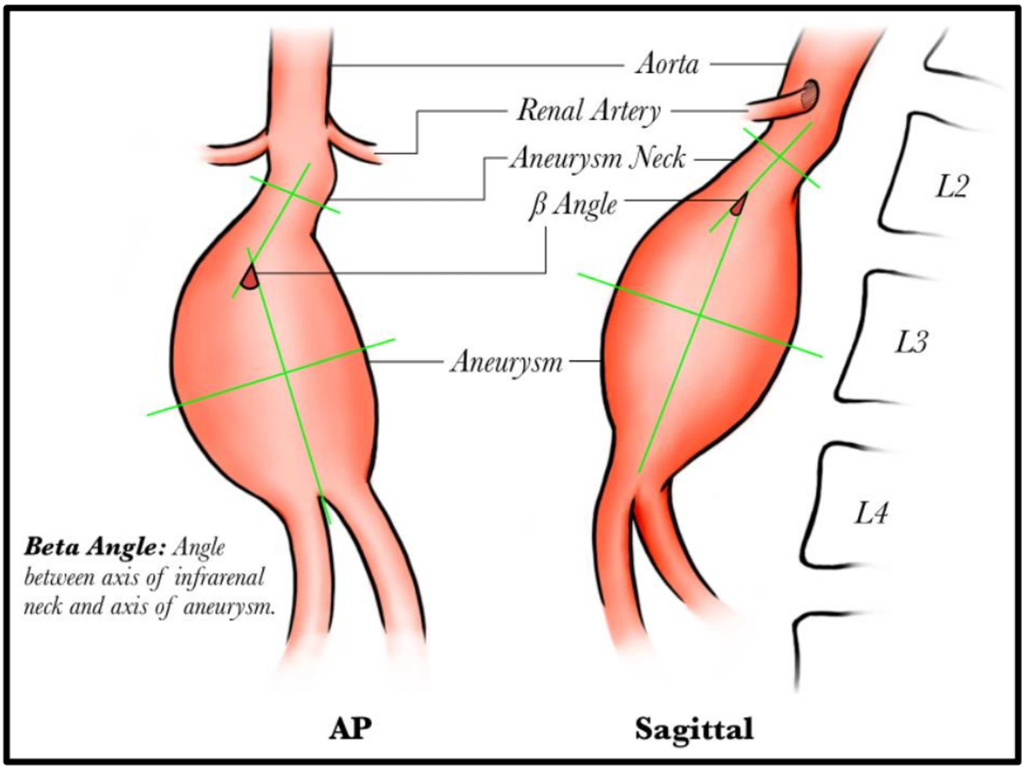

4. *Neck Calcification*

Excessive calcium load is defined as the presence of calcification affecting >1/3 circumference of the neck along a 3mm length of neck, anywhere in the first 15mm of neck (the "seal zone"), either as 1 continuous "plate" of calcium, multiple plaques or as speckled coverage.

Measurement involves profiling the neck in both coronal and sagittal views, with subsequent assessment in the orthogonal axial over 3mm lengths of neck (Figures 4a and 4b). Presence of excess calcification is determined based on a visual estimation of the 1/3 circumference threshold and recorded as binary outcome.

4a

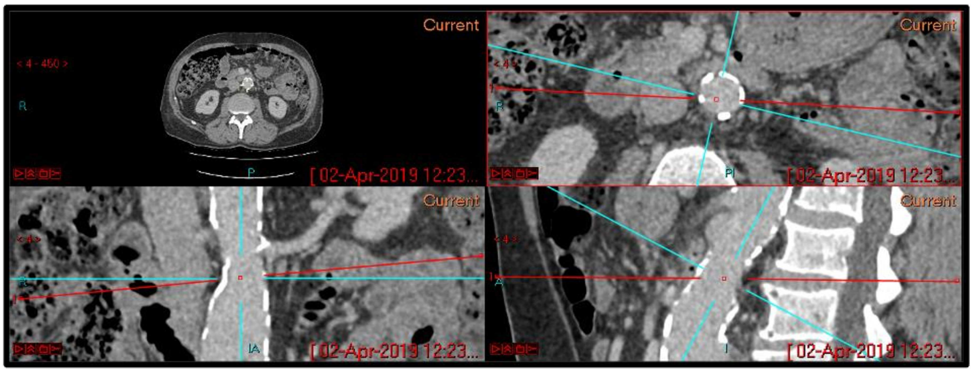

4b

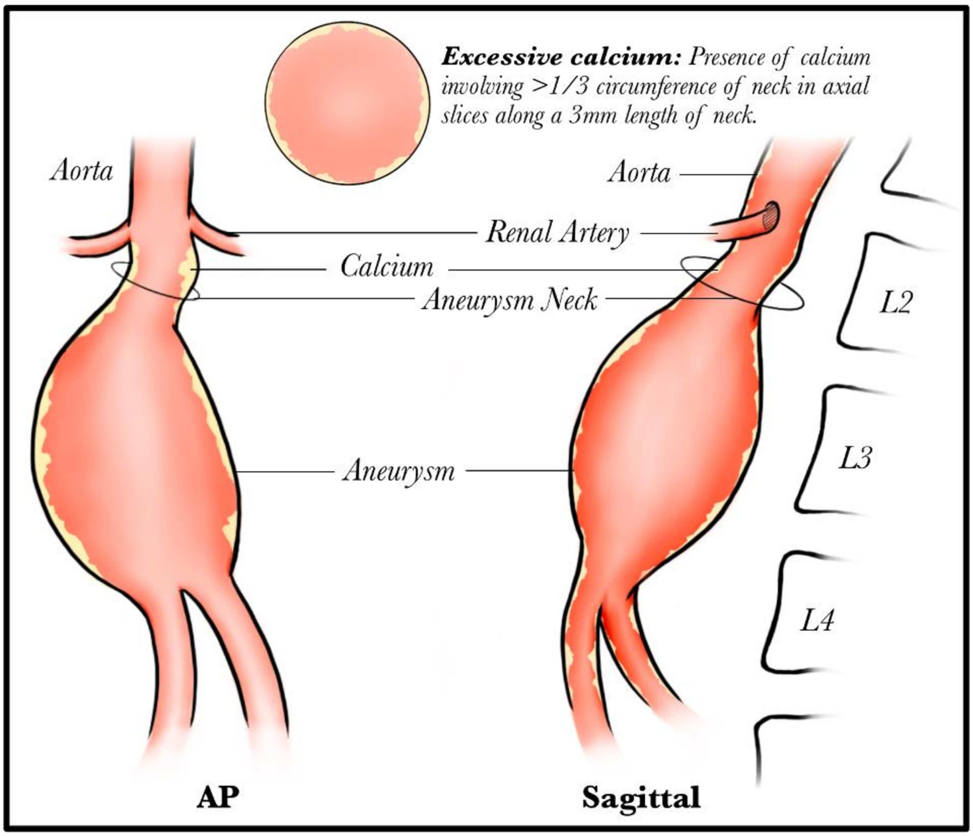

5. <u>Neck Thrombus Load</u>

Excessive thrombus load is defined as the presence of thrombus within the lumen of the aortic neck, lining >1/3 circumference of the neck OR filling 1/3 the surface area of the neck in axial slices along 3mm of neck length, anywhere in the first 15mm of neck (the "seal zone").

Assessment involves profiling the neck in both coronal and sagittal views, with subsequent assessment in the orthogonal axial view. Thrombus load is assessed on visual estimation of the 1/3 circumference threshold or 1/3 surface area threshold across multiple axial views of the neck (Figures 5a and 5b) and recorded as a binary outcome.

5a

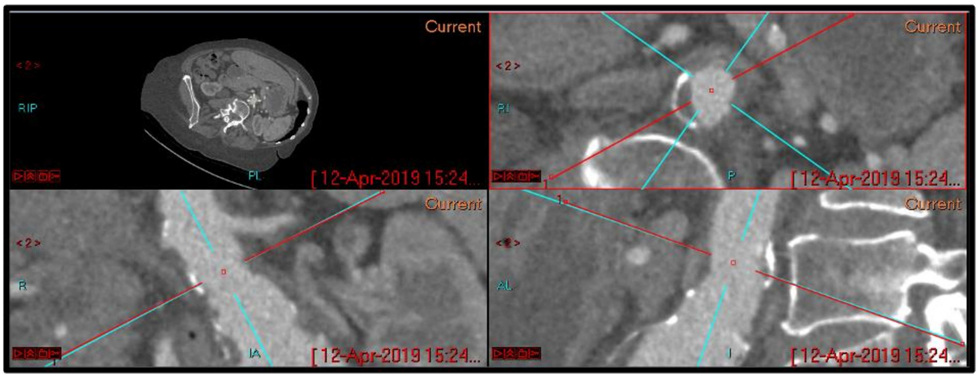

5b

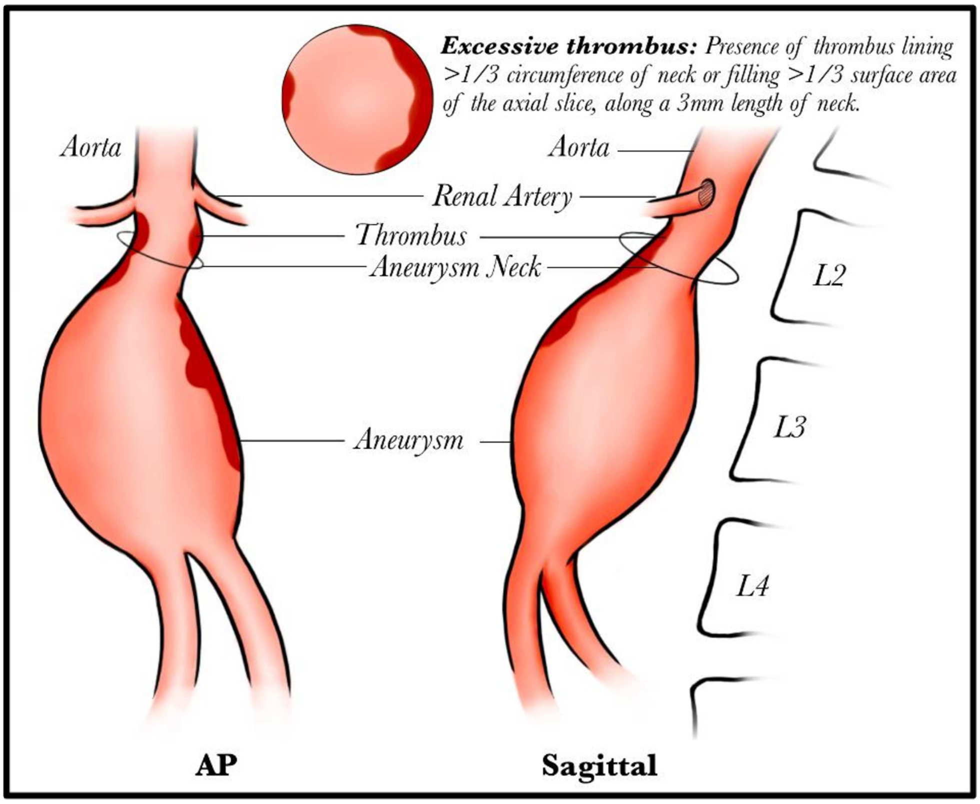

6. Neck diameters

Neck diameter is defined as the maximal outer to outer diameter (mm) in orthogonal cross section. The aneurysm neck is profiled in both coronal and sagittal views with subsequent assessment in the orthogonal axial view. Neck diameters are measured at the level of the lowermost renal artery (Figure 6a), at 5mm below this point (Figure 6b), at 10mm below this point (Figure 6c), and at 15mm below this point (Figure 6d). 15mm of neck length was chosen for diameter assessment as it is the most common length of seal zone stipulated in an EVAR stent graft's instructions for use document. The various positions along the length of the neck will be defined using the ruler function on the coronal view as seen in Figures 6a to 6e. Where the neck is <15mm in length, a neck diameter measurement will additionally be taken at the most distal point of the neck.

6a

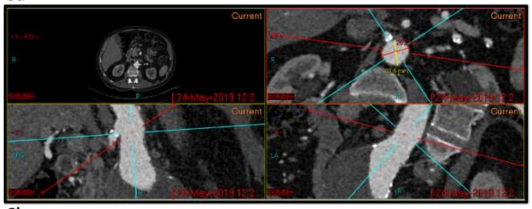

6c

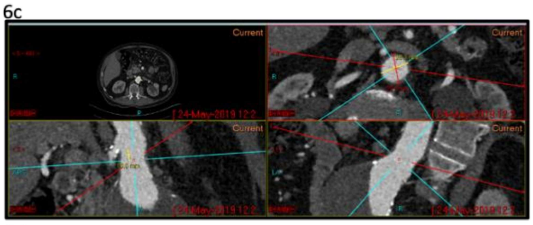

6b

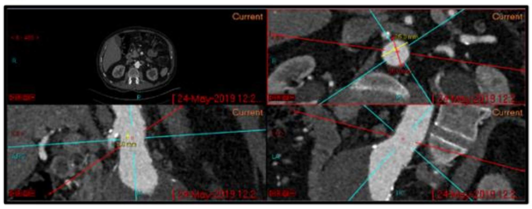

6d

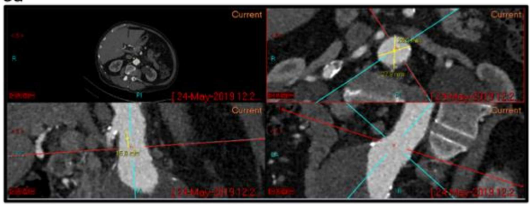

6e

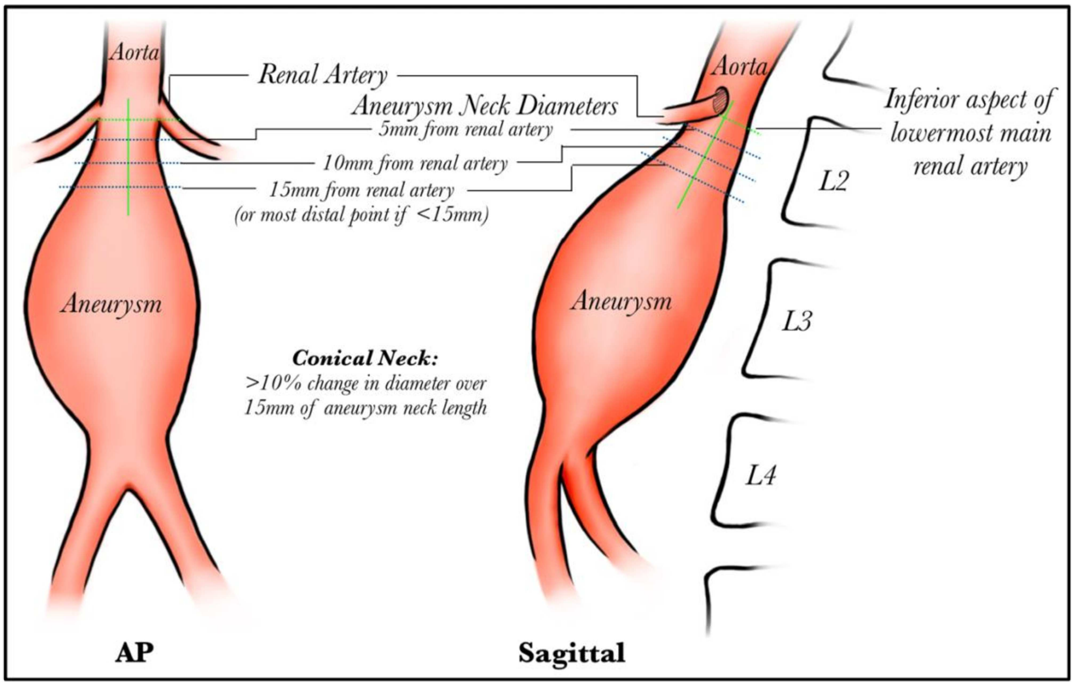

7. α neck angle

α neck angle refers to the angle, in degrees, between the axis of the suprarenal aorta and the axis of the infrarenal neck.

The first step of measurement is alignment of the axis of the neck in both coronal and sagittal planes. Tilting the double-oblique marker lines in the axial view in the next step will align the aorta in the coronal or sagittal views with the neck visible in the same field. The angle function is used to measure the angle between the neck and the suprarenal aorta and α neck angle is calculated by subtraction from 180 (Figures 7a and 7b).

7a

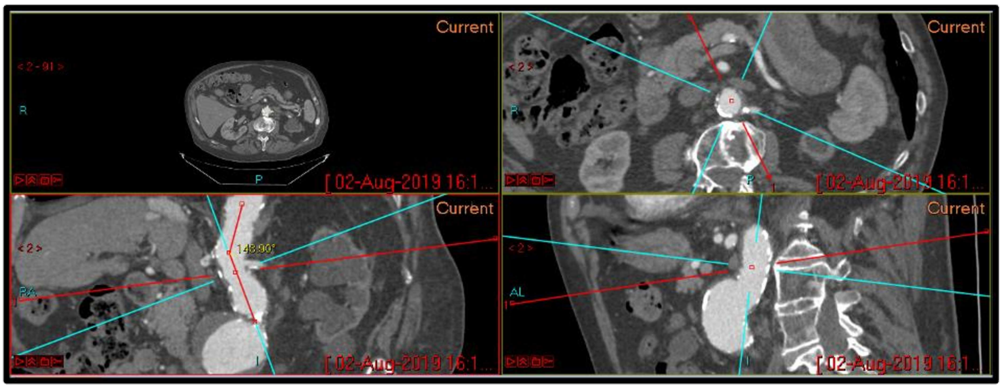

7b

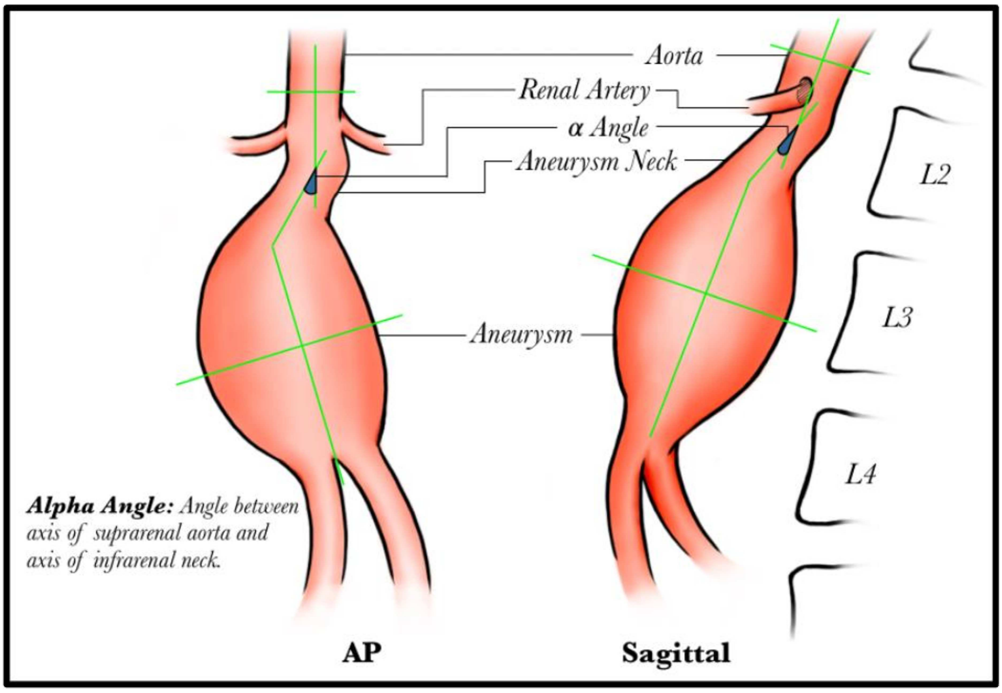

8. ***Non-straight necks***

Non-straight neck refers to the presence of intra-neck angulation >60 degrees. This is recorded as binary outcome. If present, then a further "juxtarenal neck length" is measured, referring to the length of aneurysm neck proximal to the point of angulation but below the level of the lowermost major renal artery. This is demonstrated in Figures 8a and 8b.

Measurement of intra-neck angulation utilises the same tools and techniques as described for the aforementioned α and β neck angle measurements (Figures 3 and 7). Measurement of juxtarenal neck length utilises the same tools and techniques as described for the aforementioned total neck length measurement (Figure 2).

8a

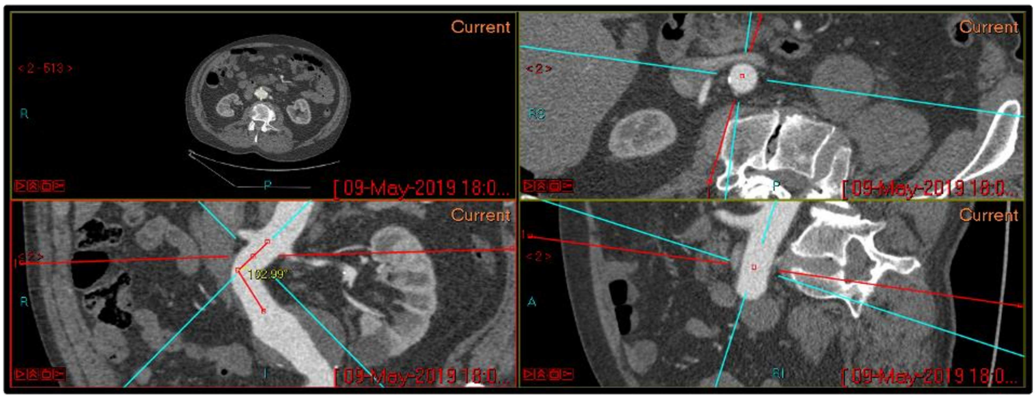

8b

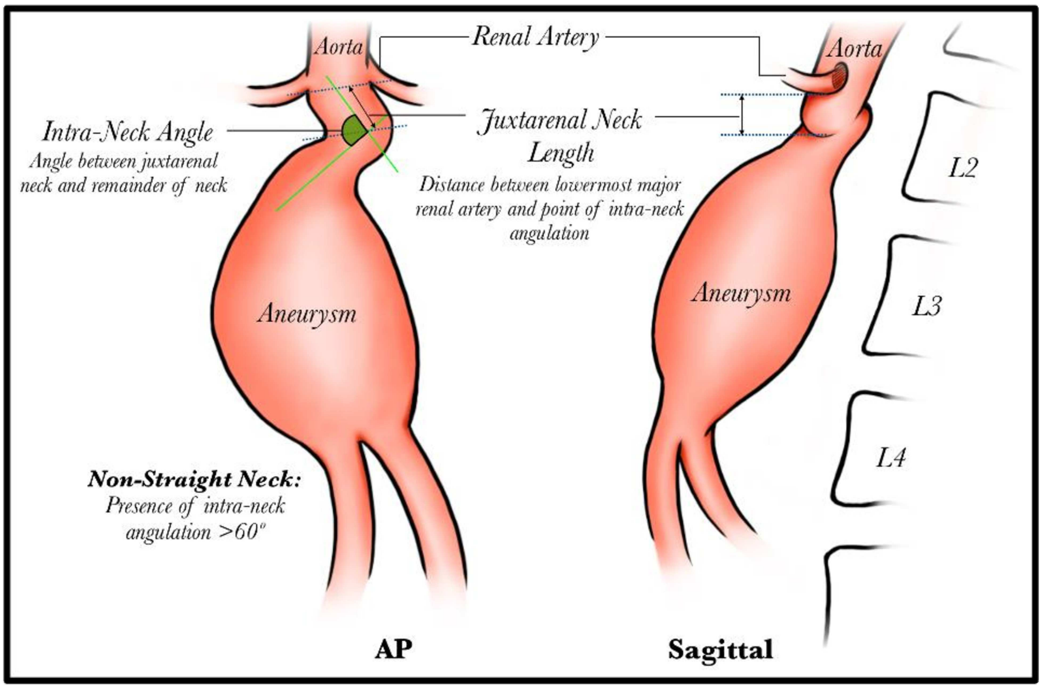

9. *Estimated Aortic Clamp Level*

This refers to the most likely position of an aortic cross clamp in the event of the aneurysm being repaired by open surgery. The levels are:

- **Infra-renal** (below the lowermost major renal artery)- if total neck length ≥5mm and free from excessive thrombus.
- **Inter-renal** (between the left and right renal arteries) - if total neck length <5mm and there is an aortic length of ≥5mm between the renal arteries.
- **Supra-renal** (between the uppermost major renal artery and the superior mesenteric artery) - if total neck length <5mm and there is no adequate inter-renal space and there is distance between uppermost renal artery and SMA ≥5mm.
- **Supra-coeliac** (above the coeliac axis) - if none of the aforementioned clamp positions are possible.

Estimating clamp level prediction requires 3 separate measurements:

1) Total neck length - this has been previously described.

2) Inter-vessel separation for renal arteries.
   This can be measured by profiling the main renal arteries on either side in the coronal view and using the ruler function to measure the distance between the two renal arteries.

3) Inter-vessel separation distance between uppermost main renal artery and SMA.
   This can be measured by profiling the renal arteries in the coronal view, marking the position of the pointer at that level so that the renal artery position can be correlated on the sagittal view. In the sagittal view, the SMA can be profiled enabling measurement of distance between the 2 with the ruler function.

Patel SR, *et al. BMJ Open* 2021; 11:e054493. doi: 10.1136/bmjopen-2021-054493

Section 4 - The UK-COMPASS Corelab protocol – internal validation of the measurement process.

An internal validation study was performed on a subset of 70 CT scans, randomly selected from a pool of the first 500 CT scans received in the Corelab. 3 assessors of varying seniority (medical student, junior vascular trainee and senior vascular trainee) undertook independent and blinded assessment of the CT scans using the Corelab Protocol measurement process described in this manuscript. Seven measurements undergoing validity testing included maximal aneurysm diameter, total neck length, beta angle and neck diameters at 4 points along a 15mm length. Additionally, the results of the two trainees (of a seniority level reasonably expected to perform the Corelab analysis) underwent analysis to investigate potential discrepancies of patient grouping (by neck length) as a result of the measurement process.

Comparisons between Rater 1 vs Rater 2, Rater 3 vs Rater 1 and Rater 3 vs Rater 2 were performed in order to calculate and summarise the inter-rater variability. A two-way mixed effects model (each measurement done by the same set of raters, who are the only possible evaluators) was used to estimate the intra-class correlations (ICC) along with 95% confidence intervals for all 7 different measurements. Furthermore, Bland-Altman plots were generated. All statistical analysis was performed using STATAv15.1 (StataCorp. 2017. Stata Statistical Software: Release 15. College Station, TX: StataCorp LLC) An alpha level of 5% was assumed.

Inter-rater variability for the 3 raters across the 7 different measurement domains is demonstrated:

Statistic	Total neck length (units)	β Neck angle	Aneurysm diameter (units)	Diameter 0 (units)	Diameter 5 (units)	Diameter 10 (units)	Diameter 15 (units)
n	210	190	210	190	184	180	164
Mean	1.03	-0.38	0.56	0.03	0.05	0.10	-0.11
SD	6.65	10.14	2.47	1.10	1.27	1.18	2.43
Minimum	-18.0	-51.0	-4.0	-3.0	-6.0	-4.0	-19.0
Maximum	31.0	19.0	15.0	5.0	5.0	4.0	3.0

Patel SR, *et al. BMJ Open* 2021; 11:e054493. doi: 10.1136/bmjopen-2021-054493

There was minimal inter-rater variability for all 7 domains as shown in these Bland-Altman plots:

Bland Altmann plots demonstrating excellent inter-rater agreement for measuring (a) neck length, (b) beta angle, (c) aneurysm diameter, (d) diameter of neck at level of renal artery, (e) diameter of neck 5mm below renal artery, (f) diameter of neck 10mm below renal artery, (g) diameter of neck 15mm below renal artery.
To generate the scatter plots below the mean value across the three observers (for each anatomical parameter) was calculated for each scan (x-axis). The average difference was calculated for each measurement for all three observers (y-axis).

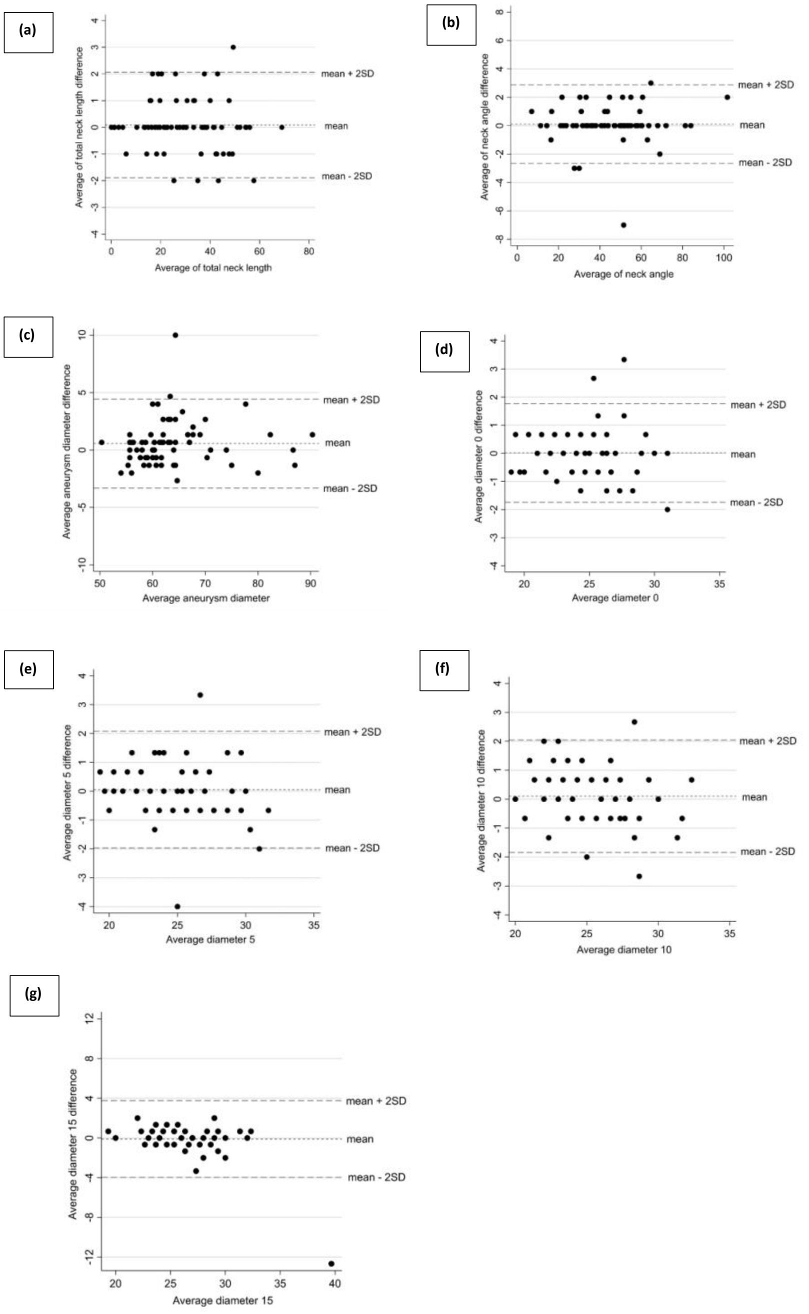

Inter-class correlation is demonstrated:

Statistic	Total neck length (units)	β Neck angle	Aneurysm diameter (units)	Diameter 0 (units)	Diameter 5 (units)	Diameter 10 (units)	Diameter 15 (units)
ICC	0.972	0.952	0.983	0.976	0.971	0.974	0.909
95% CI	0.959 – 0.982	0.927 – 0.969	0.974 – 0.989	0.964 – 0.985	0.956 – 0.982	0.960 – 0.983	0.857 – 0.945
F (df1, df2)	F(69, 138)=36.76	F(62, 124)=20.60	F(69, 138)=62.32	F(62, 124)=41.22	F(60, 120)=34.36	F(59, 118)=38.09	F(52, 104)=10.89
p-value	*<0.001*	*<0.001*	*<0.001*	*<0.001*	*<0.001*	*<0.001*	*<0.001*

A high degree of reliability was found between all measurements in all 7 domains across all 3 raters (ICC >90% for all 7 measures; p<0.001 for all).

For the grouping validation exercise of the 2 trainee measurements, a kappa statistic was calculated in order to examine inter-rater reliability. The distribution of grouping numbers for the two raters is shown:

		**Rater 1**				
		**1**	**2**	**3**	**4**	**Total**
**Rater 2**	**1**	*8*	*0*	*0*	*0*	8
	**2**	*0*	*2*	*0*	*0*	*2*
	**3**	*0*	*0*	*27*	*0*	*27*
	**4**	*0*	*0*	*0*	*33*	*33*
	**Total**	*8*	*2*	*27*	*33*	*70*

(Grp 1: length <5mm, Grp 2: length 4-9mm, Grp 3: length ≥10mm, Grp 4: not complex)

There was a perfect inter-rater agreement [k=1.00, se=0.0881], suggesting that any discrepancies in measurement did not correlate to discrepancies in subsequent grouping for analysis.

BMJ Publishing Group Limited (BMJ) disclaims all liability and responsibility arising from any reliance placed on this supplemental material which has been supplied by the author(s)

Section 5 - Microcosting exercise data collection template

Micro-costing Template for UK-COMPASS

Please complete the proforma below and provide quantities and further details in the free text boxes, in as much detail as possible e.g. brands/sizes/versions etc.

Date of procedure

1) Operation for juxtarenal aneurysm. Please tick one:

FEVAR	Open repair	Infrarenal EVAR	EVAR + Endoanchors	EVAR + Chimneys	Other (please specify)

2) Pre-operative care *(from day of diagnosis of complex aneurysm until the day of surgery)*

ITEM	Quantity	Further details
**Outpatient appt with operator**		
**Outpatient appt with other** *(Anaesthetics, another referral)*		
**Pre-assessment appt**		
**Blood tests**		
**Scans**		

Patel SR, *et al. BMJ Open* 2021; 11:e054493. doi: 10.1136/bmjopen-2021-054493

**ECG**		
**Spirometry**		
**Transthoracic ECHO**		
**Dobutamine stress ECHO**		
**Exercise stress ECHO**		
**CPET**		
**Other tests (please specify)**		

3) **Surgery** *(from arrival to hospital until the day of discharge)*

- **Theatre utilisation**

ITEM	Quantity	Further details
**Hybrid suite (time in mins)**		
**Operating theatre (time in mins)**		

**IR suite (time in mins)**		

- **Personnel**

ITEM	Quantity	Further details
**Surgeon**		
**Radiologist**		
**Assistants**		
**Anaesthetist**		
**Anaesthetist Juniors**		
**ODP**		
**Scrub nurse**		
**Other theatre staff (please specify)**		

- **Medications**

ITEM	Quantity	Further details

**Anaesthetic Drugs**		
**Antibiotics**		
**Heparin**		
**Other intra-operative meds (please specify)**		

- **Standard Equipment**

ITEM	Quantity	Further details
**Anaesthetic equipment (endotracheal tube, cannulas, other lines etc.)**		
**Theatre trays**		
**Skin prep**		
**Drapes**		

**Other general theatre equipment (please specify)**		

- **Specialist Equipment**

ITEM	Quantity	Further details
**USS machine for puncture**		
**Puncture kit with standard J wire x2 (for both sides)**		
**Initial Sheaths x2 (with puncture, both sides)**		
**Large Sheaths (for device delivery and cannulation, both sides)**		
**Pigtail catheter**		
**Stiff guidewire x2 (for both sides)**		
**PROXIMAL MAIN BODY (fenestrated piece if FEVAR)**		
**Catheters for target vessels (if appropriate)**		

**Guidewires for target vessels (if appropriate)**		
**Sheaths for target vessels (if appropriate)**		
**Target vessel stents (for FEVAR and Chimney cases)**		
**DISTAL MAIN BODY (for FEVAR)**		
**CONTRALATERAL LIMB + ADDITIONAL LIMBS ON EITHER SIDE**		
**Coda Balloon**		
**Contrast Agent**		
**Other specialist equipment (please specify)**		

4) Post-operative care *(from day 1 post-op to discharge from service/death)*

- **Inpatient stay**

ITEM	Quantity	Further details
**Length of stay in bed on ward (level 1)**		

**Length of stay in Level 2 bed**		
**Length of stay in Level 3 bed**		
**Nursing care**		
**Physiotherapy care**		
**OT care**		
**Intermediate care if applicable**		

- **Outpatient care**

ITEM	Quantity	Further details
**Follow-up clinic appointments**		
**Scans**		
**Community Nursing input**		
**GP visits**		
