# Supplementary material for: A risk-adjusted and anatomically stratified cohort comparison study of open surgery, endovascular techniques and medical management for juxtarenal aortic aneurysms—the UK COMPlex AneurySm Study (UK-COMPASS): a study protocol
Source: BMJ Open. 2021 Nov 30;11(11):e054493. doi: 10.1136/bmjopen-2021-054493 (PMC8634354; doi:10.1136/bmjopen-2021-054493)
Supplement: Author's manuscript [file bmjopen-2021-054493.draft_revisions.pdf]

BMJ Open is committed to open peer review. As part of this commitment we make the peer review history of every article we publish publicly available.

When an article is published we post the peer reviewers' comments and the authors' responses online. We also post the versions of the paper that were used during peer review. These are the versions that the peer review comments apply to.

The versions of the paper that follow are the versions that were submitted during the peer review process. They are not the versions of record or the final published versions. They should not be cited or distributed as the published version of this manuscript.

BMJ Open is an open access journal and the full, final, typeset and author-corrected version of record of the manuscript is available on our site with no access controls, subscription charges or pay-per-view fees (http://bmjopen.bmj.com).

If you have any questions on BMJ Open's open peer review process please email info.bmjopen@bmj.com

BMJ Open

A risk-adjusted and anatomically stratified cohort comparison study of open surgery, endovascular techniques and medical management for juxtarenal aortic aneurysms: The UK COMPlex AneurySm Study (UK-COMPASS).

Journal:	*BMJ Open*
Manuscript ID	bmjopen-2021-054493
Article Type:	Protocol
Date Submitted by the Author:	13-Jun-2021
Complete List of Authors:	Patel, Shaneel; Liverpool University Hospitals NHS Foundation Trust, Vascular Surgery; University of Liverpool, Liverpool Centre for Cardiovascular Science<br>Ormesher, David; Liverpool University Hospitals NHS Foundation Trust, Vascular Surgery<br>Smith, Samuel; University of Liverpool, School of Medicine<br>Wong, Kitty; North Bristol NHS Trust, Vascular Surgery<br>Bevis, Paul; North Bristol NHS Trust, Vascular Surgery<br>Bicknell, Colin; Imperial College London, Department of Surgery and Cancer<br>Boyle, Jonathan ; Cambridge University Hospitals NHS Foundation Trust, Vascular Surgery<br>Brennan, John; Liverpool University Hospitals NHS Foundation Trust, Vascular Surgery<br>Campbell, Bruce; Royal Devon and Exeter Hospital, Vascular Surgery<br>Cook, Andrew; University of Southampton, Wessex Institute<br>Crosher, Alastair; Liverpool University Hospitals NHS Foundation Trust, Radiology<br>Duarte, Rui; University of Liverpool, Liverpool Reviews and Implementation Group<br>Flett, Murray; Ninewells Hospital, Vascular Surgery<br>Gamble, Carrol; University of Liverpool, Liverpool Clinical Trials Centre<br>Jackson, R; University of Liverpool, Liverpool Clinical Trials Centre<br>Juszczak, Maciej; University Hospitals Birmingham NHS Foundation Trust, Vascular Surgery<br>Loftus, Ian ; St George's Healthcare NHS Trust, Vascular Surgery<br>Nordon, Ian; University Hospital Southampton NHS Foundation Trust, Vascular Surgery<br>Patel, Jai; Leeds Teaching Hospitals NHS Trust, Interventional Radiology<br>Platt, Kellie; University of Liverpool, Liverpool Clinical Trials Centre<br>Psarelli, Eftychia; University of Liverpool, Liverpool Clinical Trials Centre<br>Rowlands, Peter; Liverpool University Hospitals NHS Foundation Trust, Radiology<br>Smyth, John; Manchester University NHS Foundation Trust, Vascular Surgery<br>Spachos, Theodoros ; Lancashire Teaching Hospitals NHS Foundation Trust, Vascular Surgery<br>Taggart, Leigh; University of Liverpool, Liverpool Clinical Trials Centre

	Taylor, Claire; University of Liverpool, Liverpool Clinical Trials Centre<br>Vallabhaneni, Srinivasa; Liverpool University Hospitals NHS Foundation Trust, Vascular Surgery; University of Liverpool, Liverpool Centre for Cardiovascular Science
Keywords:	Vascular surgery < SURGERY, Clinical trials < THERAPEUTICS, Protocols & guidelines < HEALTH SERVICES ADMINISTRATION & MANAGEMENT, VASCULAR SURGERY

SCHOLARONE™
Manuscripts

I, the Submitting Author has the right to grant and does grant on behalf of all authors of the Work (as defined in the below author licence), an exclusive licence and/or a non-exclusive licence for contributions from authors who are: i) UK Crown employees; ii) where BMJ has agreed a CC-BY licence shall apply, and/or iii) in accordance with the terms applicable for US Federal Government officers or employees acting as part of their official duties; on a worldwide, perpetual, irrevocable, royalty-free basis to BMJ Publishing Group Ltd ("BMJ") its licensees and where the relevant Journal is co-owned by BMJ to the co-owners of the Journal, to publish the Work in this journal and any other BMJ products and to exploit all rights, as set out in our licence.

The Submitting Author accepts and understands that any supply made under these terms is made by BMJ to the Submitting Author unless you are acting as an employee on behalf of your employer or a postgraduate student of an affiliated institution which is paying any applicable article publishing charge ("APC") for Open Access articles. Where the Submitting Author wishes to make the Work available on an Open Access basis (and intends to pay the relevant APC), the terms of reuse of such Open Access shall be governed by a Creative Commons licence – details of these licences and which Creative Commons licence will apply to this Work are set out in our licence referred to above.

Other than as permitted in any relevant BMJ Author's Self Archiving Policies, I confirm this Work has not been accepted for publication elsewhere, is not being considered for publication elsewhere and does not duplicate material already published. I confirm all authors consent to publication of this Work and authorise the granting of this licence.

For peer review only

BMJ Open - Protocol submission

Title

A risk-adjusted and anatomically stratified cohort comparison study of open surgery, endovascular techniques and medical management for juxtarenal aortic aneurysms: The UK COMPlex AneurySm Study (UK-COMPASS).

Authors

Shaneel R Patel[1,2], David C Ormesher[1], Samuel R Smith[3], Kitty H F Wong[4], Paul Bevis[4], Colin D Bicknell[5], Jonathan R Boyle[6], John A Brennan[1], Bruce Campbell[7], Andrew Cook[8], Alastair P Crosher[1], Rui V Duarte[9], Murray M Flett[10], Carrol Gamble[11], Richard J Jackson[11], Maciej T Juszczak[12], Ian M Loftus[13], Ian M Nordon[14], Jai V Patel[15], Kellie Platt[11], Eftychia-Eirini Psarelli[11], Peter C Rowlands[1], John V Smyth[16], Theodoros Spachos[17], Leigh Taggart[11], Claire Taylor[11], Srinivasa R Vallabhaneni[1,2]

1 Liverpool University Hospitals NHS Foundation Trust, United Kingdom
2 Liverpool Centre for Cardiovascular Science, University of Liverpool, United Kingdom
3 School of Medicine, University of Liverpool, United Kingdom
4 North Bristol NHS Trust, United Kingdom
5 Department of Surgery and Cancer, Imperial College London, United Kingdom
6 Cambridge University Hospitals NHS Foundation Trust, United Kingdom
7 Royal Devon and Exeter Hospital, United Kingdom
8 Wessex Institute, University of Southampton, United Kingdom
9 Department of Health Services Research, University of Liverpool, United Kingdom
10 Ninewells Hospital, Dundee, Scotland, United Kingdom
11 Liverpool Clinical Trials Centre, University of Liverpool, United Kingdom
12 University Hospitals Birmingham NHS Foundation Trust, United Kingdom
13 St George's University Hospitals NHS Foundation Trust, United Kingdom
14 University Hospital Southampton NHS Foundation Trust, United Kingdom
15 The Leeds Teaching Hospitals NHS Trust, United Kingdom
16 Manchester University NHS Foundation Trust, United Kingdom
17 Lancashire Teaching Hospitals NHS Foundation Trust, United Kingdom

Corresponding Author (and reprint requests to):
Shaneel Patel
8c Link, Royal Liverpool Hospital
Prescot Street
Liverpool
L7 8XP
shaneel.patel@nhs.net
+447824396578

For peer review only

Abstract

Introduction

In a third of all abdominal aortic aneurysms (AAA) the aneurysm neck is short (juxtarenal) or shows other adverse anatomical features rendering operations potentially more complex, hazardous and expensive. Surgical techniques used are open surgical repair and endovascular aneurysm repair (EVAR) techniques including Fenestrated EVAR, EVAR with adjuncts (chimneys/endoanchors) and off-label standard EVAR. The aim of UK-COMPASS is to answer the research question identified by the NIHR HTA Programme: '*What is the clinical and cost-effectiveness of strategies for the management of juxtarenal abdominal aortic aneurysm, including fenestrated endovascular repair?*'.

Methods and Analysis

UK-COMPASS is an empirical cohort study comparing clinical and cost-effectiveness of different strategies used to manage complex AAAs with stratification of physiological fitness and anatomical complexity and use of statistical methods to correct for baseline risk and indication biases. There are two data streams. First, a stream of routinely collected data from Hospital Episode Statistics and National Vascular Registry. Preoperative computerised tomography scans of all patients who underwent elective AAA repair in England between 1st November 2017 and 31st October 2019 are subjected to Corelab analysis to accurately identify and include every complex aneurysm treated without further selection or randomisation. Secondly, a site-reported data stream regarding quality-of-life and treatment costs from prospectively recruited patients across England. Site recruitment also includes patients with complex aneurysms larger than 55 mm diameter in whom an operation is deferred (Medical management). The primary outcome measure is perioperative all-cause mortality. Follow-up will be to a median of 5 years.

Ethics and Dissemination

The study has received full regulatory approvals from a Research Ethics Committee, the Confidentiality Advisory Group and the Health Research Authority. Data sharing agreements are in place with NHS Digital and the National Vascular Registry/HQIP. Dissemination will be via NIHR HTA reporting, peer-reviewed journals and conferences.

Trial Registration Number ISRCTN85731188

For peer review only

Strengths and Limitations of this study

(5 bullet points, 1 sentence each, related to methods)

- UK-COMPASS will include all patients undergoing repair of a complex AAA in England over a 24-month period in a comparative effectiveness analysis of high external validity.
- This study will involve the largest Corelab analysis of CT aortograms ever conducted to identify complex aneurysms according to predefined objective criteria pertaining to aneurysm neck anatomy.
- This study will capture every treatment method used to repair complex aneurysms.
- This study will incorporate methods of accounting for baseline risk and indication biases arising from physiological status and anatomical complexity, which will improve internal validity of comparative effectiveness analyses.
- The main limitation of the study design is a lack of random allocation, which leaves potential for unknown biases remaining uncorrected.

For peer review only

Introduction

Approximately 4000 individuals undergo elective repair of an abdominal aortic aneurysm (AAA) each year in the United Kingdom[1] with the intention of preventing premature death from aneurysm rupture. The two distinct repair methods used, open surgical repair (OSR) and endovascular aneurysm repair (EVAR), each have unique advantages and disadvantages. OSR is safer within the perioperative period, but more durable in the years to follow compared to EVAR. Treatment costs also differ between the two methods.

The complexity of aneurysm repair depends mainly upon anatomical detail relating to the segment of un-dilated aorta between renal arteries and the aneurysm, referred to as the aneurysm “neck”. Ideal aneurysm neck has normal diameter in cross section which is uniform along its length with little angulation, thrombus or calcification. Infrarenal aneurysms with at least 10-15mm length of ideal aneurysm neck are amenable to OSR with aortic occlusion clamp below the renal arteries. Such anatomy also allows standard EVAR within ‘Instructions for Use’ (IFU) defined by manufacturers of stent-grafts implanted during EVAR. Some 40-60% of aneurysms fall within this category[2-4] and there is a wealth of comparative effectiveness evidence relating to such patients[5, 6]. The remainder, whose aneurysm neck is too short (commonly called “juxtarenal”) or otherwise unsuitable for standard EVAR within IFU (on-label device use), are pragmatically referred to as having a “complex aneurysm”.

OSR of complex aneurysms involve aortic occlusion clamps at a variety of levels; below the renal arteries (infrarenal), below the superior mesenteric artery (suprarenal) or above the coeliac artery (supravisceral or supracoeliac) with increasing levels of physiological burden and technical difficulty as the clamp level moves higher. Complex aneurysms also call for advanced EVAR techniques such as fenestrated endovascular aneurysm repair (FEVAR), and EVAR with adjuncts (including chimneys and endoanchors)[7], all of which are more complex than standard EVAR. Standard EVAR outside IFU (off-label standard EVAR) is also used in routine clinical practice to treat complex aneurysms.

Repair of complex aneurysms by any technique is associated with greater risk than repair of standard aneurysms. Therefore, in patients with aneurysms larger than the conventional treatment threshold of 55mm diameter and poor physiological status, a shared decision may be made to defer operation until the risk versus benefit turns favourable towards intervention. Such ‘Medical management’ of complex aneurysms with continued surveillance beyond the 55mm size threshold has never been compared to immediate operation.

Existing evidence for different methods of repair of complex aortic aneurysms is largely in the form of case series. Most published analyses are limited by selection and indication biases. The practice of offering OSR to physiologically fitter patients and reserving EVAR techniques for the less fit patients is seldom accounted for in analyses comparing outcomes [8-10]. Furthermore, in most analyses patients are grouped by the operation techniques used rather than the detail of the anatomy being treated. A lack of precise and objective information regarding aneurysm neck anatomy, pooling of short-neck aneurysms with aneurysms without a neck as well as aneurysms of the visceral aortic segment and thoracoabdominal aortic aneurysms seriously limits the value of most published evidence[11-14]. These sources of bias,

confounding and heterogeneity have been consistently recognised as weaknesses in the evidence base by authors of systematic reviews[15, 16].

There is therefore a pressing need to generate comparative effectiveness evidence in this area with adequate internal and external validity. Expert opinion sought by the study funder, as well as an independently conducted workshop revealed that in the United Kingdom, a randomised controlled trial comparing OSR against FEVAR (the two principal repair methods) would be not be feasible due mainly to a lack of sufficient equipoise. Additionally, such a trial would neglect other methods of complex aneurysm repair that are widely used, including off-label EVAR and EVAR with adjuncts (such as endoanchors).

Endovascular treatments are associated with greater cost than OSR due to the cumulative costs of the stent-grafts, ancillary devices, postoperative surveillance, and reintervention. National Health Service (NHS) clinical service commissioners have noted an increasing demand for FEVAR in the absence of evidence regarding clinical effectiveness identifying which patients would benefit from this procedure and whether the substantial cost associated with the technique is an appropriate use of NHS resources. Prohibitive cost and lack of experience with FEVAR in many arterial centres has concomitantly led to a substantial use of off-label standard EVAR in complex neck anatomy, an intervention that is known to have poorer long-term results compared to on-label EVAR [17]. The National Institute for Health Research Health Technology Assessment (NIHR HTA) programme have commissioned this comparative research study in order to resolve this NHS decision problem.

The primary aim of UK-COMPASS is to answer the research question identified by the NIHR HTA Programme: '*What is the clinical and cost-effectiveness of strategies for the management of juxtarenal abdominal aortic aneurysm, including fenestrated endovascular repair?'.* The five objectives of this study are shown in Table 1.

For peer review only

Methods and Analysis

Overview

UK-COMPASS is an empirical cohort study comparing the clinical and cost-effectiveness of different management strategies used to repair complex aneurysms, incorporating statistical methods of correcting for confounding by physiological risk and anatomical complexity.

The study incorporates aspects which has been referred to by NIHR as "efficient study design", where a substantial proportion of data required for the analysis is retrieved from routinely collected sources. This reduces the cost of conducting research, facilitates inclusion of a large number of patients within a short time frame, and reflects "real world" practice. The study has two streams of data collection:

1. Routinely collected data:

This stream pertains to patients who have undergone repair of a complex aneurysm in England, over a 2-year period between 1st November 2017 and 31st October 2019. Health data, demographic data and health care resource use data relating to operated patients will be retrieved from Hospital Episode Statistics datasets (NHS Digital), the National Vascular Registry (HQIP), and the NHS Picture Archiving and Communication System (PACS). Median follow-up will be 5-years, until 31st October 2023.

The National Vascular Registry (NVR) is run by the Clinical Effectiveness Unit of the Royal College of Surgeons of England as a national clinical audit. At least 85% of AAAs treated in England are included[1], with at least 85% data completeness[18]. The NVR includes a wide range of data relevant to this study, including demographics, comorbidity, preoperative assessment, intraoperative detail, postoperative complications, duration of hospital stay and critical care use for all types of AAA repair.

Hospital Episode Statistics (HES) is a data warehouse containing details of all admissions, outpatient appointments, investigations, and emergency department attendances at National Health Service (NHS) hospitals in England. These data are collected during a patient's time at hospital and is designed to enable administration as well as medical research. In addition to health and resource use data during the primary admission for aneurysm repair, follow-up data will be collected for five years after the operation to identify late complications, treatment of complications and re-interventions. Survival data is retrieved via Office for National Statistics (ONS) data linked to HES.

2. Site-reported data:

a. Quality of Life (QoL) data prospectively reported by consenting patients (both Operated and Medical Management) from participating arterial centres in England will supplement the routinely collected data. Recruitment will be undertaken between 1st November 2017 and 31st December 2022, with follow-up until 31st December 2023.

For peer review only

b. Baseline Clinical Data: Participating centres will report baseline health and demographic data for patients recruited to the QoL aspect of the study.

Primary outcome measures are perioperative mortality, late all-cause mortality and late aneurysm-related mortality.

Secondary outcome measures are perioperative complication rates (including organ system complications, infections, and complications specific to stent-grafts), perioperative secondary interventions, length of stay, intensive care usage, late complication rates (including complications specific to stent-grafts, renal failure/dialysis, aneurysm complications, infection, incisional hernia, late secondary intervention and health economic measures including incremental cost effectiveness ratio in terms of cost per incremental gain in quality adjusted life years.

All interventions that have been used in the study population will be compared as numbers allow. We anticipate methods of repair to include open surgery (OSR) with a variety of clamp levels (infrarenal, suprarenal or supravisceral), fenestrated endovascular aneurysm repair (FEVAR), off-label use of standard EVAR and EVAR with adjuncts (including chimneys and endoanchors).

Methods

1. Routinely collected data

The study population of complex aneurysms for the routinely collected data stream will be identified through anonymised Corelab analysis of pre-operative computerised tomography (CT) scans; a Corelab refers to a combination of infrastructure and methodology of image analysis according to predetermined reporting standards and definitions. Corelab CT analysis will be undertaken for all abdominal aortic aneurysms repaired in England between 1st November 2017 and 31st October 2019. These patients and details regarding their pre-operative CT scans will be identified from the Hospital Episode Statistics (HES) dataset and Diagnostic Imaging Dataset (DID) of NHS Digital. This will permit retrieval of said CT scans from the provider site to the Corelab based at the Royal Liverpool University Hospital using the Internet Exchange Portal (IEP) and PACS.

The CT scans will be analysed in the Corelab using a pre-specified protocol that was devised using a clinical consensus exercise conducted with experts from throughout the United Kingdom in December 2019. The Corelab protocol is provided in full in the Online Supplementary Material file and includes information on anatomical definitions, in-depth information regarding the measurement process, as well as the results of a validation exercise. The anatomical CT-based inclusion and exclusion criteria for the routinely collected data stream are summarised in Table 2.

It is anticipated that approximately 8000 pre-operative CT scans will be analysed in the Corelab and that of these, approximately 3000 patients will meet the anatomical inclusion criteria for a complex neck. These patients are included in the analysis without further selection or randomisation. Pseudonymised baseline and postoperative follow-up data (up to

For peer review only

5 years) will be provided for this population of patients with complex necks, using datasets from NHS Digital and the National Vascular Registry. Additionally, intra-operative images and postoperative surveillance for the patients treated by endovascular techniques will be retrieved to the study Corelab.

Site-reported data

All arterial vascular centres in England are eligible to participate as recruitment centres for the site-reported data stream. Recruitment will be undertaken between 1st November 2017 and 31st December 2022, with follow up QoL data collected until 31st December 2023.

Patients will be prospectively identified at recruiting sites and approached for QoL follow-up using Good Clinical Practice (GCP) compliant processes. The anatomical inclusion criteria used to identify the patients mirror those of routinely collected data (see Table 2). Patients will be eligible for contribution to QoL data collection if they are due to have a complex aneurysm managed with open or endovascular surgery, as well as cases that have reached 55mm in maximal diameter and are selected to remain on extended surveillance ('Medical Management') with decision for treatment made at a later date once the aneurysm has reached a larger threshold diameter.

In addition to the anatomical exclusion criteria listed in Table 2, cases utilising surgeon-modified stent-grafts and cases in which patients are outright turned down for surgery due to co-morbidity or patient choice ('Operation Declined') will be ineligible for recruitment to QoL follow-up.

Both generic (EQ-5D-5L[19]) and aneurysm-specific (Aneurysm-DQoL[20]) QoL questionnaires will be used to collect data according to pre-specified time points for both Operated and Medically Managed patients as shown in Table 3. The data will be collected in the form of written completed questionnaires or telephone interviews, in accordance with patient preference. A patient diary will also be completed by patients annually to provide data on health resource use following their aneurysm treatment.

Recruiting sites will also be asked to conduct a microcosting exercise in order to assess variations in endovascular consumables and local practice. A detailed cost profile will be obtained for at least ten patients treated by each treatment modality: OSR, FEVAR and EVAR (+/-adjuncts). A proforma for microcosting data collection is included in the Online Supplementary Material file.

Sample size calculations

Perioperative mortality:

The primary outcome measure is perioperative death. The primary efficacy parameter is the odds ratio comparing FEVAR to both open repair and off label EVAR. For OSA, conservative estimate of 8% peri-operative death might be expected, and a reduction to 4 % could be considered clinically relevant, which is equivalent to an odds ratio of 0.48 (based on NVR 2015 report[21] and GLOBALSTAR registry[22]). This effect is slightly larger than that noted in

For peer review only

randomised controlled trials of standard infrarenal aneurysm repair[5]. A Bonferroni adjusted two-sided alpha level of 0.025 is used. Based on these assumptions, collecting data on 2000 patients will ensure a power in excess of 80% if the allocation between treatments is relatively equal and should be preserved above 70% even if one treatment strategy is used twice as much as the other two.

Stratified comparative analysis for perioperative mortality and identification of risk factors:

Based on the best available estimates, it is anticipated that 120 events (perioperative deaths) will be available if 2000 patients are included in the study. The aim is to identify anatomical/physiological characteristics for which there is a differential effect on patient outcome depending on the treatment strategy employed. We plan to use multivariable models which include propensity scores as continuous covariates, adjusted for appropriate confounding variables and including interaction effects with treatment effect. Using the statistical rule of thumb of 10 events per variable, approximately 12 variables can be included in any single model. If, for example, a two-level factor is to be investigated, main effects and interaction effects and a covariate of propensity scores will account for 6 variables, allowing for a further inclusion of other confounding variables as required.

Long term survival; all-cause mortality:
Estimated survival rates at 1, 2 and 4 years are 90%, 80% and 70% respectively. All patients will have a median of 5 years follow-up after entering the study. It is approximated that 2000 patients should then contribute approximately 550 events (all-cause mortality after 30 days/discharge). As the study is not designed to identify a difference in long-term overall survival, no Power calculation is provided to detect some minimum clinically relevant difference. Instead, assuming the difference between two treatment arms to be measured using a log hazard ratio it is estimated that from 550 events, a standard error of approximately 0.085 will be observed. This translates to a 95% confidence interval of length of approximately 0.34. Here, a hazard ratio smaller than 0.71 or larger than 1.40 will be shown to be significant at a 5% level.

Data analysis plan

Analyses will be carried out on an intention-to-treat basis, retaining all patients in the surgery group to which they are assigned irrespective of any procedural issues. The intervention groups will be OSR, FEVAR, EVAR +/- adjuncts (including chimneys and endoanchors). The exact treatment received will be determined by triangulation of NVR data and HES data for OSR. Patients receiving endovascular treatment will be identified from NVR data and HES data, and exact technical details of each procedure (including the use of adjuncts) will be determined through analysis of procedural images in the Corelab.

As an analysis of observational cohort data, all attempts to present a measure of efficacy free from confounding bias will be undertaken. Patients will be stratified by infrarenal neck length for analysis into 3 groups: <5mm (juxtarenal), 5-9mm (juxtarenal) and ≥10mm neck length (not juxtarenal but complex based on device-specific IFU criteria). This will serve to limit anatomical heterogeneity and bias within comparisons. Additionally, patients will be

For peer review only

stratified by physiological risk. The British Aneurysm Repair score[23] will be used (11 clinical parameters available from National Vascular Registry data) to divide each comparison group into those that are high-risk (the highest risk quartile) and the remainder as standard risk.

Statistical analyses of the primary outcome shall follow the principle of propensity score analysis. Propensity score estimation is chosen for the analysis of the primary endpoint instead of multivariable regression techniques because it is considered the most appropriate means of accounting for selection bias at the low level of peri-operative death rates anticipated[24]. Propensity scores estimate the probability of a patient being given each treatment strategy based on baseline clinical and demographic characteristics. Patients can then be stratified based on their propensity to receive each treatment strategy and an estimate of treatment effect is then obtained, which adjusts for potential selection bias in the data.

Models to estimate the propensity for each patient shall be generated using multi-variable logistic modelling techniques using as candidate covariates only variables that influence simultaneously the treatment assignment and the outcome variable. Covariates shall be retracted to demographic/clinical data available at the point of treatment decision with the aim of producing a propensity model that satisfies the assumptions of conditional independence. The final analysis shall be carried out using stratified logistic where the strata are defined by the quintiles of the fitted propensity score values. Prior to analyses it shall be confirmed that each stratum contains at least 20 observed perioperative deaths. If not, the strata will be combined with an adjacent strata level.

The key efficacy parameter of interest will be an odds ratio presented with a 97.5% confidence interval. Results will be presented as a forest plot with the odds ratio within each stratum presented along with the overall effect.

It is recognised that recruitment for site-reported quality of life data will occur either side of a cessation of elective services in the United Kingdom due to the COVID-19 pandemic in 2020/2021. There may be differences in patient behaviour and clinical practice before and after this period and we aim to investigate this further in the analysis phase. Firstly, outcome data before and after the dates of interest will be compared. Secondly, clinical practice before and after the dates of interest will be compared using data from the National Vascular Registry.

Health Economic (HE) Analysis

The UK-COMPASS study will adapt the recent NICE Health Economic Analysis model[25] which will facilitate updates of NICE Clinical Guideline 'Abdominal Aortic Aneurysm – Diagnosis and Management (NG156)'.

Our aim is to inform NICE decision-making in a timely manner. The NICE HE model has been developed from a UK NHS and Personal Social Services perspective, appraised by a NICE Committee and scrutinised during consultation, and we see this fit to be adapted without developing a de novo model.

For peer review only

The purpose of the health economic analysis in this study is defined in the original objectives 4 & 5. The main outputs therefore will remain A) estimation of incremental cost effectiveness ratios comparing different treatment strategies in terms of life years and quality adjusted life years gained, and B) to identify whether particular level of physiological fitness (stratified into two groups) and / or anatomical baseline characteristics (stratified into three groups) are associated with better health economic efficiency using one or other treatment strategy.

At least five-year follow-up is considered the essential minimum to capture attrition of treatment utility and accrual of treatment costs that occur from late failure/secondary intervention and postoperative surveillance.

For peer review only

Ethics and Dissemination

The study protocol, informed consent form, patient information sheet and all other patient facing study documentation has received the favourable opinion of the North West – Preston Research Ethics Committee (REC) and Health Research Authority (HRA) approval.

Access to patient data in NHS Digital datasets, the NVR dataset as well as to imaging, without the need for patient consent, has been granted by the Confidentiality Advisory Group (CAG); CAG is an independent body which provides expert advice to the HRA and the Secretary of State for Health on whether applications to access confidential patient or service user information without consent should or should not be approved.

Summary of approval data (including latest amendments/extensions):
REC approval – 30/11/2017
CAG approval – 13/12/2017
Full HRA approval – 18/12/2017
NHS Digital Data Sharing Agreement (latest) – 01/11/2020
HQIP NVR Data Sharing Agreement (latest) – 21/09/2020

The study is managed by the Liverpool Clinical Trials Centre. The sponsor for the study is Liverpool University Hospitals NHS Foundation Trust

Dissemination plan

The results of this study will be written up and published as an official report for the NIHR HTA. It will also be published in a peer-reviewed journal and presented at international conferences.

Authors' contributions

All authors contributed to writing and/or editing of this protocol manuscript and all supplementary information. Additional specific roles are as follows:

- Chief investigator: *SRV*.
- Writing of the trial protocol, writing of the Corelab protocol and performance of the validation exercise: *SRP, DCO, SRS, KHFW and SRV.*
- Co-investigators: *CDB, IML, JRB, JAB, JVP, AC, BC, JVS, PCR, RJJ and RVD*.
- Trial centre and host site staff responsible for coordinating trial activity, retrieving imaging data, general data management and statistics: *APC, CG, RJJ, DCO, SRP, KP, EEP, LT and CT*.
- Clinical Consensus Group members responsible for development of the Corelab protocol, anatomical definitions, and groupings for analysis: *PB, CDB, JRB, JAB, MMF, MTJ, IMN, SRP, TS and SRV.*

Funding statement

This study is funded solely by NIHR HTA programme. Total funding contracted is £1,266,687.59. This funding source had no role in the design of this study but oversee the study through regular progress reports and monitoring to ensure the study is delivered in accordance with contractual arrangements.

For peer review only

Competing interests statement

There are no competing interests to declare.

Registration details

ISRCTN registration: ISRCTN85731188

We would like to acknowledge the following UK-COMPASS committees

- ***Trial Steering Committee (TSC)***
  Janet Powell (Independent chair), Florian Dick (Independent specialist), Jonathan Michaels (Independent specialist, Health Economics), Andrew Tambyraja (Independent specialist) and Paul Massey (Public and Patient Involvement representative)

- ***Independent Data Monitoring and Safety Committee (IDMSC)***
  Mike Roberts (Chair), Viktoria McMillan, Ulrike Naumann and Murray Flett

- ***Trial Management Group (TMG)***
  Barbara Arch, Colin Bicknell, James Boateng, Jonathan Boyle, Bruce Campbell, Andrew Cook, Alastair Crosher, Rui Duarte, Carol Gamble, Richard Jackson, David Ormesher, Shaneel Patel, Kellie Platt, Heather Rogers, Leigh Taggart, Claire Taylor and Syed Yusuf

Word Count excluding references

3635

For peer review only

Table 1 – The UK-COMPASS study objectives

**Objective 1**	To compare different treatment strategies for their perioperative mortality and morbidity, corrected for confounding physiological and anatomical characteristics, in order to account for baseline risk and indication biases.
**Objective 2**	To identify whether particular physiological and/ or anatomical baseline characteristics are associated with better clinical outcomes or better health economic efficiency using one or other treatment strategy.
**Objective 3**	To compare different treatment strategies in terms of overall survival and in terms of treatment failure in the long-term follow-up (stent-graft related complications, secondary interventions, aneurysm-related mortality).
**Objective 4**	To perform cost effectiveness analyses from NHS and personal social care perspective to establish incremental cost effectiveness ratios, comparing different treatment strategies in terms of cost per incremental gain in and quality adjusted life years.
**Objective 5**	To establish the clinical and cost utility of FEVAR and alternatives, in patients who are considered physiologically unfit for OSR, and to compare these against medical managements.

For peer review only

Table 2 – CT-based anatomical inclusion and exclusion criteria for the routinely collected data stream

Inclusion criteria	Exclusion criteria
Aneurysm diameter ≥55mm AND neck length <10mm	Ruptured aneurysm
Aneurysm diameter ≥55mm AND neck length ≥10mm AND the presence of ≥1 of the following adverse neck features:<br>- Beta (β) angle >60°<br>- Conicality (>10% change in diameter along 15mm length of neck)<br>- Thrombus lining >1/3 circumference of the neck OR filling 1/3 the surface area of the neck along a 3mm length of neck<br>- Calcium load in the wall of the neck affecting >1/3 circumference of the neck along a 3mm length of neck<br>- Non-straight neck, defined by the presence of intra-neck angulation of >60 degrees with a juxtarenal neck length of <15mm<br>- Alpha (α) angle >45°	Subthreshold aneurysm (<55m)
	Thoracic aortic aneurysm
	Thoracoabdominal aortic aneurysm
	Visceral aortic aneurysm (where aorta at level of SMA is ≥30mm)

For peer review only

Table 3 – Quality of Life data collection schedule

QoL tool	Pre-op	1-month post-op	3-months post-op	6-months post-op	1-year post-op	Annually up to 5 years post-op
**Operation Patients**						
EQ-5D-5L	✓	✓		✓	✓	✓
Aneurysm-DQoL	✓		✓		✓	✓
Health Diary					✓	✓
**Medically Managed Patients**						
EQ-5D-5L	✓			✓	✓	✓
Aneurysm-DQoL	✓				✓	✓
Health Diary					✓	✓

For peer review only

References

1. Waton S, Johal A, Birmpili P, Li Q, Cromwell D, Pherwani A, O'Neill R and Boyle J. National Vascular Registry: 2020 Annual Report. 2020.

2. Giménez-Gaibar A, González-Cañas E, Solanich-Valldaura T, Herranz-Pinilla C, Rioja-Artal S and Ferraz-Huguet E. Could Preoperative Neck Anatomy Influence Follow-up of EVAR? *Ann Vasc Surg*. 2017;43:127-133.

3. Marone EM, Freyrie A, Ruotolo C, Michelagnoli S, Antonello M, Speziale F, Veroux P, Gargiulo M and Gaggiano A. Expert Opinion on Hostile Neck Definition in Endovascular Treatment of Abdominal Aortic Aneurysms (a Delphi Consensus). *Ann Vasc Surg*. 2020;62:173-182.

4. Patelis ND, Malli A, Mylonas KS, Schizas D, Papa N, Economopoulos KP, Damaskos C, Moulakakis K, Katsargyris A, Georgopoulos S, Klonaris C and Liakakos T. Suitability study of current endovascular aortic repair devices based on real-life anatomic data. *Expert Rev Med Devices*. 2019;16:165-171.

5. Stather PW, Sidloff D, Dattani N, Choke E, Bown MJ and Sayers RD. Systematic review and meta-analysis of the early and late outcomes of open and endovascular repair of abdominal aortic aneurysm. *Br J Surg*. 2013;100:863-72.

6. Powell JT, Sweeting MJ, Ulug P, Blankensteijn JD, Lederle FA, Becquemin JP and Greenhalgh RM. Meta-analysis of individual-patient data from EVAR-1, DREAM, OVER and ACE trials comparing outcomes of endovascular or open repair for abdominal aortic aneurysm over 5 years. *Br J Surg*. 2017;104:166-178.

7. Buck DB, van Herwaarden JA, Schermerhorn ML and Moll FL. Endovascular treatment of abdominal aortic aneurysms. *Nat Rev Cardiol*. 2014;11:112-23.

8. O'Donnell TFX, Boitano LT, Deery SE, Schermerhorn ML, Schanzer A, Beck AW, Green RM, Takayama H and Patel VI. Open Versus Fenestrated Endovascular Repair of Complex Abdominal Aortic Aneurysms. *Ann Surg*. 2020;271:969-977.

9. Locham S, Dakour-Aridi H, Bhela J, Nejim B, Bhavana Challa A and Malas M. Thirty-Day Outcomes of Fenestrated and Chimney Endovascular Repair and Open Repair of Juxtarenal, Pararenal, and Suprarenal Abdominal Aortic Aneurysms Using National Surgical Quality Initiative Program Database (2012-2016). *Vasc Endovascular Surg*. 2019;53:189-198.

10. Michel M, Becquemin JP, Clément MC, Marzelle J, Quelen C and Durand-Zaleski I. Editor's choice - thirty day outcomes and costs of fenestrated and branched stent grafts versus open repair for complex aortic aneurysms. *Eur J Vasc Endovasc Surg*. 2015;50:189-96.

11. Bannazadeh M, Beckerman WE, Korayem AH and McKinsey JF. Two-year evaluation of fenestrated and parallel branch endografts for the treatment of juxtarenal, suprarenal, and thoracoabdominal aneurysms at a single institution. *J Vasc Surg*. 2020;71:15-22.

12. O'Donnell TFX, Patel VI, Deery SE, Li C, Swerdlow NJ, Liang P, Beck AW and Schermerhorn ML. The state of complex endovascular abdominal aortic aneurysm repairs in the Vascular Quality Initiative. *J Vasc Surg*. 2019;70:369-380.

13. Tsilimparis N, Perez S, Dayama A and Ricotta JJ, 2nd. Endovascular repair with fenestrated-branched stent grafts improves 30-day outcomes for complex aortic aneurysms compared with open repair. *Ann Vasc Surg*. 2013;27:267-73.

14. Sala-Almonacil VA, Zaragozá-García JM, Ramírez-Montoya M, Molina-Nácher V, Martínez-Perelló I and Gómez-Palonés FJ. Fenestrated and chimney endovascular aneurysm repair versus open surgery for complex abdominal aortic aneurysms. *J Cardiovasc Surg (Torino)*. 2017;58:801-813.

For peer review only

15. Nordon IM, Hinchliffe RJ, Holt PJ, Loftus IM and Thompson MM. Modern treatment of juxtarenal abdominal aortic aneurysms with fenestrated endografting and open repair--a systematic review. *Eur J Vasc Endovasc Surg*. 2009;38:35-41.
16. Rao R, Lane TR, Franklin IJ and Davies AH. Open repair versus fenestrated endovascular aneurysm repair of juxtarenal aneurysms. *J Vasc Surg*. 2015;61:242-55.
17. Antoniou GA, Georgiadis GS, Antoniou SA, Kuhan G and Murray D. A meta-analysis of outcomes of endovascular abdominal aortic aneurysm repair in patients with hostile and friendly neck anatomy. *J Vasc Surg*. 2013;57:527-38.
18. Grant SW, Sperrin M, Carlson E, Chinai N, Ntais D, Hamilton M, Dunn G, Buchan I, Davies L and McCollum CN. Calculating when elective abdominal aortic aneurysm repair improves survival for individual patients: development of the Aneurysm Repair Decision Aid and economic evaluation. *Health Technol Assess*. 2015;19:1-154, v-vi.
19. Herdman M, Gudex C, Lloyd A, Janssen M, Kind P, Parkin D, Bonsel G and Badia X. Development and preliminary testing of the new five-level version of EQ-5D (EQ-5D-5L). *Qual Life Res*. 2011;20:1727-36.
20. Romaine J, Peach G, Thompson M, Hinchliffe RJ and Bradley C. Psychometric validation of three new condition-specific questionnaires to assess quality of life, symptoms and treatment satisfaction of patients with aortic aneurysm. *J Patient Rep Outcomes*. 2019;3:29.
21. National Vascular Registry: 2015 Annual Report. 2015.
22. Early results of fenestrated endovascular repair of juxtarenal aortic aneurysms in the United Kingdom. *Circulation*. 2012;125:2707-15.
23. Grant SW, Hickey GL, Grayson AD, Mitchell DC and McCollum CN. National risk prediction model for elective abdominal aortic aneurysm repair. *Br J Surg*. 2013;100:645-53.
24. Cepeda MS, Boston R, Farrar JT and Strom BL. Comparison of logistic regression versus propensity score when the number of events is low and there are multiple confounders. *Am J Epidemiol*. 2003;158:280-7.
25. Béliveau A, Boyne DJ, Slater J, Brenner D and Arora P. BUGSnet: an R package to facilitate the conduct and reporting of Bayesian network Meta-analyses. *BMC Med Res Methodol*. 2019;19:196.

For peer review only

Online Supplementary Material for UK-COMPASS protocol

Contents

Section	Content	Pages
Section 1	The UK-COMPASS Corelab protocol – details on CT scan management, quality control, protocol development and clinical consensus decision-making.	2-3
Section 2	Definitions used in the Corelab protocol measurement process (Table)	4
Section 3	The UK-COMPASS Corelab protocol – the measurement process	5-13
Section 4	The UK-COMPASS Corelab protocol – internal validation of the measurement process.	14-16
Section 5	Microcosting exercise data collection template to be used in the site-recruited data stream.	17-23

For peer review only

Section 1 - The UK-COMPASS Corelab protocol – details on Computerised Tomography (CT) scan management, quality control and protocol development.

UK-COMPASS's Corelab is a fully digital and paper-free facility in keeping with NIHR's carbon reduction guidelines.

CT scan management

CT scan management involves the retrieval, pseudonymisation and upload of CT scans for Corelab analysis in line with regulatory approvals. It is performed by the 'Research PACS Clerk' who is a trained member of the UK-COMPASS team and an employee of the host NHS trust. The process is conducted in compliance with the principles of The General Data Protection Regulation (GDPR), tailored by the Data Protection Act 2018, with appropriate training for all members of the study team.

The date and location of the most recent CT scan of the abdominal aorta, prior to the date of the patient's operation, is identified from Hospital Episodes Statistics (administrative data warehouse containing details of all admissions, outpatient appointments and emergency department attendances at NHS hospitals in England). This CT scan Digital Imaging and Communications in Medicine (DICOM) data is retrieved via the NHS Picture Archiving Communication System (PACS) utilising NHS number, date of birth and the date of specific CT scan in question, as provided by NHS Digital under a data sharing agreement. Legal basis was secured to access patient data without consent through the Secretary of State's Section 251 approval with Confidentiality Advisory Group (CAG) recommendation. Upon receipt, the scan data is irreversibly pseudonymised. This process was initially carried out using Carestream Vue PACS v11.4.1.1011 software (Carestream Health, Rochester, NY, USA), but later Osirix MD (Osirix, Geneva, Switzerland) was used for faster processing. This results in removal of all identifiers from the DICOM dataset such as name, date of birth, NHS and local hospital ID numbers. These data are replaced with a unique Study ID number which acts as pseudonym. It is not possible for the study team to re-identify any patient from this point.

The pseudonymised scans are uploaded onto a research section of the local PACS server, entitled the "Vendor Neutral Archive" (VNA), accessible via a two-stage authentication process and only to members of the UK-COMPASS team approved for Corelab analysis. Analysis of these scans is therefore blinded. It is possible to see the Study ID and date of scan acquisition when a CT is loaded for analysis, but not possible to re-identify a patient.

Quality Control of Management process

The Research PACS Clerk removes identifiable data from a retrieved CT scan using the PACS computer programme. Successful completion of pseudonymisation is verified for each scan in a 3-step quality control process. **Step 1:** The PACS clerk will perform complete anonymisation at DICOM data level upon receipt of the CT scan. **Step 2**: CT image set placed into an upload portal and success of anonymisation is verified. **Step 3:** The PACS clerk will retrieve the CT image set from the VNA into the Carestream viewing client and conduct a final check.

As of June 2021, of the 8613 scans that had been measured in the Corelab, no scans were found to have failed anonymisation confirming robustness of the quality control process.

For peer review only - http://bmjopen.bmj.com/site/about/guidelines.xhtml

Development of measurement protocol

The Corelab protocol was initially devised by consensus between three researchers (Chief Investigator: SV and two clinical research fellows: SP and DO) following literature review regarding anatomical features of complexity with respect to the neck of abdominal aortic aneurysms. With paucity of information regarding Corelab set-up in the published literature, content was determined by consensus and subsequent approval by members of the UK-COMPASS Clinical Consensus Group.

Clinical consensus exercise

A Clinical Consensus Group made up of Vascular Surgeons and relevant multidisciplinary experts from the U.K. met (December 2019) to decide anatomical definitions and a priori grouping for analysis. Corelab procedures were provided in advance. Suggestions and discussion were undertaken in a focus group format with opportunity for all to express opinion before reaching consensus through voting.

It was decided that neck length is the morphological feature of most interest with regards to complex aneurysms. Grouping by neck length in mm would provide clinical relevance to the findings and limit anatomical heterogeneity. Furthermore, the degree to which an infrarenal neck is short will influence which specific endovascular techniques are likely to be employed as well as whether the aortic clamp during OSR can be placed below the renal arteries. Therefore, the following primary anatomical stratification was recommended:

Group 1: neck length 4mm or shorter
Group 2: neck length 5-9mm
Group 3: neck length ≥10mm AND unsuitable for standard EVAR within IFU

Within group 3, it is appreciated that there will be a variety of adverse neck features present other than neck length (conicality, angulation, thrombus burden and calcification). It may be possible to separate these depending on the numbers included but the a priori analysis plan will be to group these cases.

Large neck diameter is an adverse neck feature. It was felt that 30mm should be the distinction between normal aortic neck and aneurysmal aorta. Therefore, it is recognised that there may be cases of uniform necks with adequate length but large diameter (≥30mm) in Group 1. These can be removed and interrogated as a subgroup analysis. There will therefore be no large diameter necks in Group 3.

For peer review only - http://bmjopen.bmj.com/site/about/guidelines.xhtml

Section 2 - Definitions used in the Corelab protocol measurement process (Table)

Measurement terminology	Unit of measurement	Definition
**Total neck length**	Millimetres (mm)	Distance between:<br>a) Lowermost major renal artery and,<br>b) - Point of abrupt change in calibre *(if the neck is parallel sided and <30mm diameter)* OR,<br>- where the aorta reached 30mm in diameter
**Neck diameter**	Millimetres (mm)	Orthogonal diameter of infrarenal neck at specified distances (0mm, 5mm, 10mm and 15mm) below the origin of the lowermost major renal artery (outer wall to outer wall diameter)
**α neck angle**	Degrees (°)	Angle between axis of suprarenal aorta and axis of infrarenal neck
**β neck angle**	Degrees (°)	Angle between axis of infrarenal neck and axis of aneurysm
**Non-straight neck**	Binary	Presence of intra-neck angulation >60°
**Intra-neck angle** *(when non-straight neck present)*	Degrees (°)	Angle between juxtarenal neck and remainder of neck
**Juxtarenal neck length** *(when non-straight neck present)*	Millimetres (mm)	Distance between lowermost major renal artery and point of intra-neck angulation
**Aneurysm Diameter**	Millimetres (mm)	Maximum orthogonal diameter of aneurysm (outer-to-outer)
**Excessive thrombus**	Binary	Presence of thrombus lining >1/3 circumference of neck or filling >1/3 surface area of the axial slice, along a 3mm length of neck
**Excessive calcification**	Binary	Presence of calcium involving >1/3 circumference of neck in axial slices along a 3mm length of neck

For peer review only - http://bmjopen.bmj.com/site/about/guidelines.xhtml

Section 3 - The UK-COMPASS Corelab protocol – the measurement process

Image analysis was initially performed using the multi-planar reconstruction function of Carestream Vue PACS v11.4.1.1011 software (Carestream Health, Rochester, NY, USA). After completion of measurement on approximately 3000 scans, the host site installed an updated version of the same programme (v12.2.2.1025) for the measurement process of the remainder. h

CT series of the abdominal aorta with arterial phase contrast were loaded in double-oblique multiplanar reconstructed (MPR) format. This permitted visualisation of the aneurysm/aneurysm neck in axial, sagittal and coronal views. Contrast windowing and magnification were considered baseline adjustments prior to commencement of the measurement process, which is detailed below for specific measures:

1. *Aneurysm diameter*

Both the coronal and sagittal planes are aligned with the long axis of the aneurysm body (not the flow lumen) using double oblique adjustment. This allows orthogonal axial view of the aneurysm at its largest point. A ruler function is used to measure the maximal (outer to outer) diameter of the aneurysm in the axial plane (Figures 1a and 1b).

1a

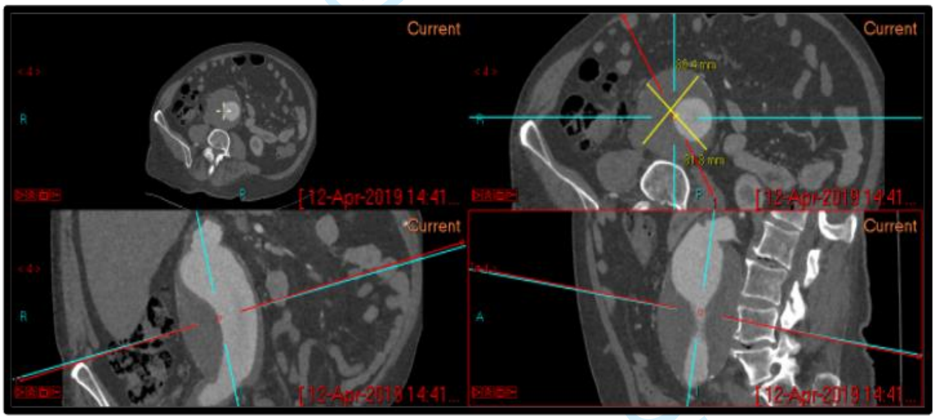

1b

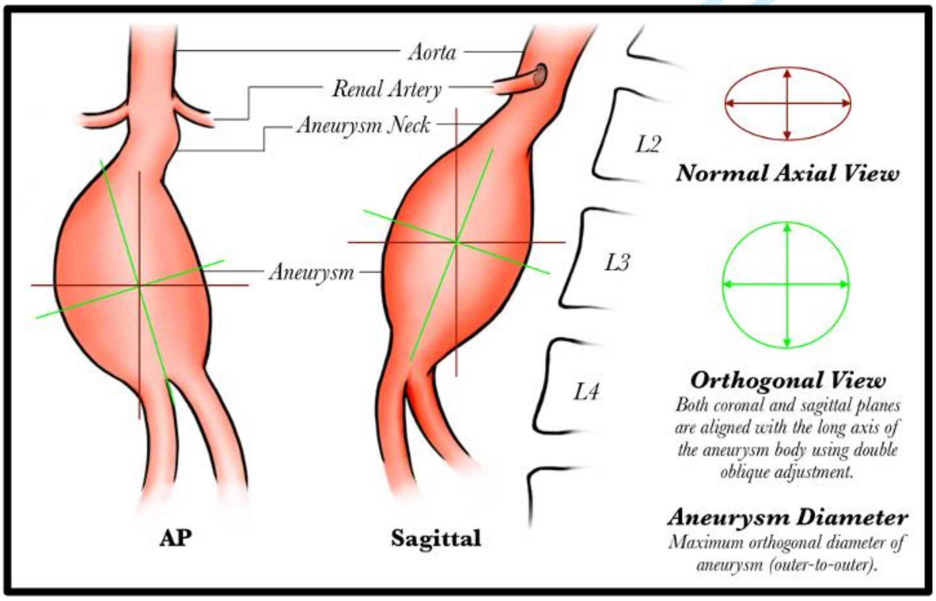

For peer review only - http://bmjopen.bmj.com/site/about/guidelines.xhtml

2. <u>Total neck length</u>

Total neck length is defined as the distance between the inferior aspect of the lowermost main renal artery and the superior aspect of the aneurysm.

The superior aspect of the aneurysm is defined as:

- The transition between normal aorta and aneurysm, where the aneurysm commences abruptly (if the neck is parallel sided and <30mm diam), OR
- the point at which the neck diameter becomes 30mm in cases where the transition to aneurysm is not abrupt.

Long axis of the aneurysm neck is aligned to both coronal and sagittal planes and the distal margin of the lower most renal artery is profiled by aligning double-oblique planes to the renal artery ostium in the axial view. A ruler function is used to measure the neck length in both coronal and sagittal views (Figures 2a and 2b).

2a

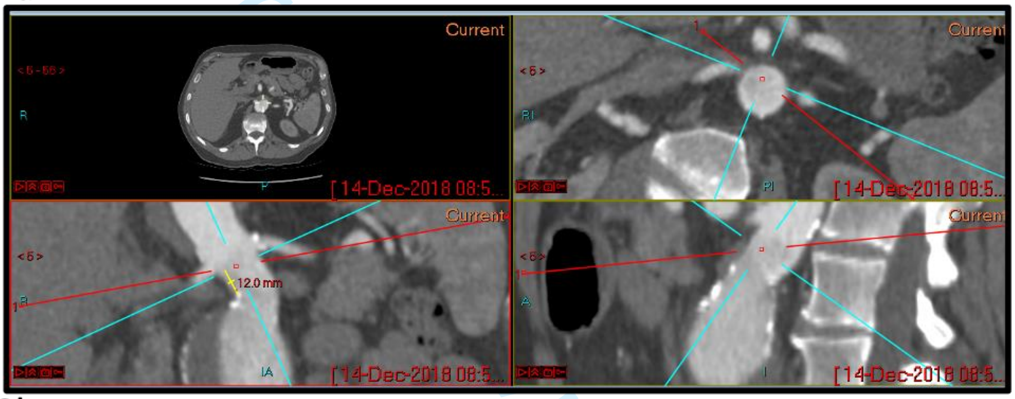

2b

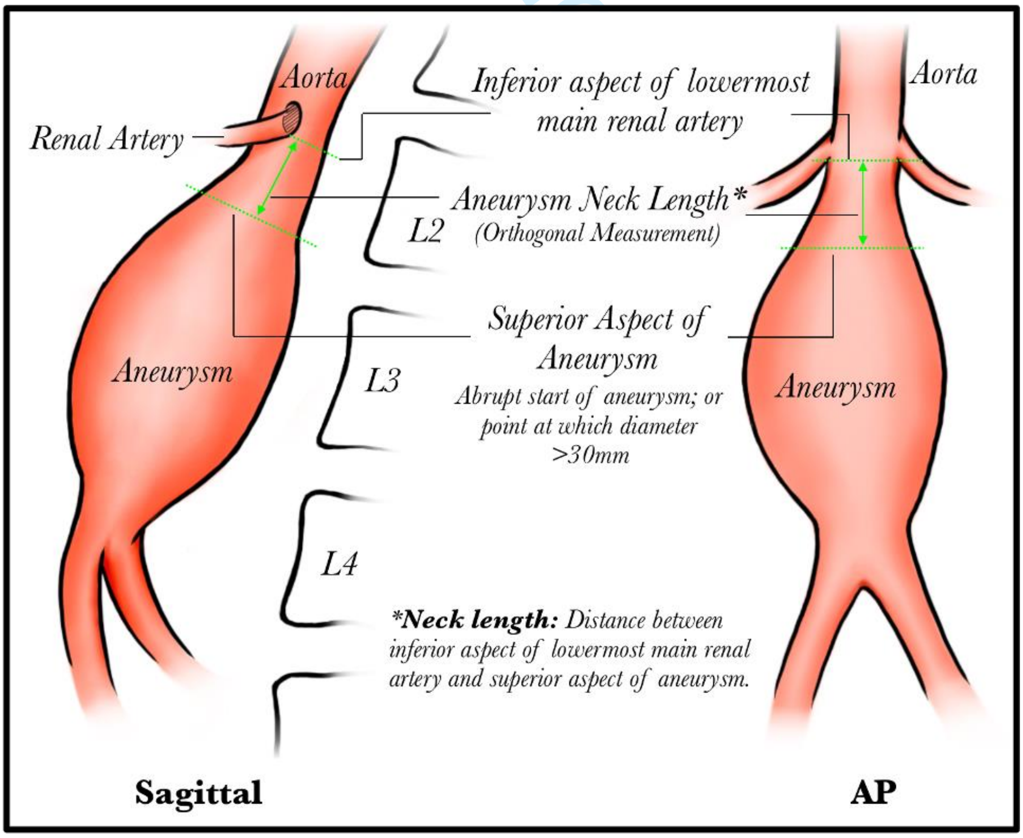

3. β neck angle

Beta (β) neck angle refers to the angle between the axis of the neck and the axis of the aneurysm, measured in degrees.

The first step is to align both coronal and sagittal planes to the axis of the aneurysm neck. Tilting the double-oblique marker lines in the axial view will permit visualisation of the aneurysm axis in the coronal or sagittal views. The largest possible angle between the axis of the neck and the axis of the aneurysm is measured and subtracted from 180 to calculate the β neck angle (Figures 3a and 3b).

3a

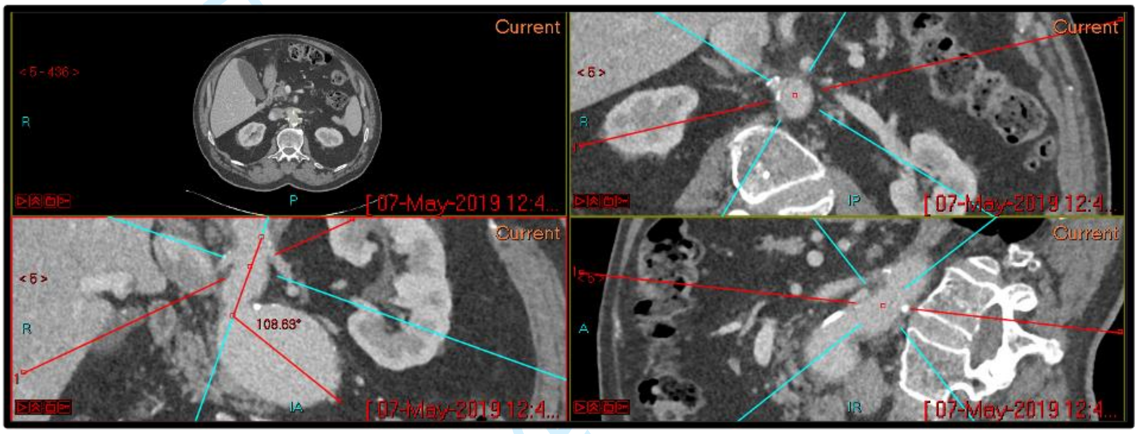

3b

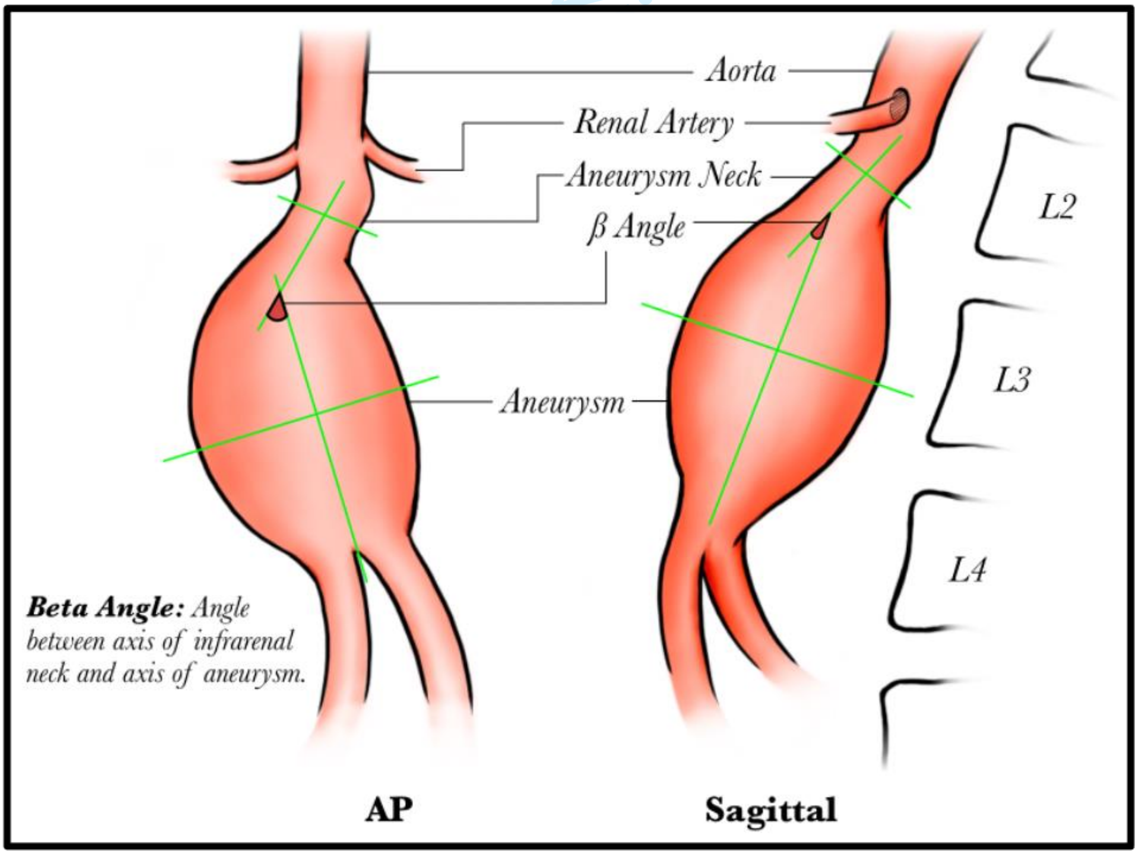

4. <u>*Neck Calcification*</u>

Excessive calcium load is defined as the presence of calcification affecting >1/3 circumference of the neck along a 3mm length of neck, anywhere in the first 15mm of neck (the "seal zone"), either as 1 continuous "plate" of calcium, multiple plaques or as speckled coverage.

Measurement involves profiling the neck in both coronal and sagittal views, with subsequent assessment in the orthogonal axial over 3mm lengths of neck (Figures 4a and 4b). Presence of excess calcification is determined based on a visual estimation of the 1/3 circumference threshold and recorded as binary outcome.

4a

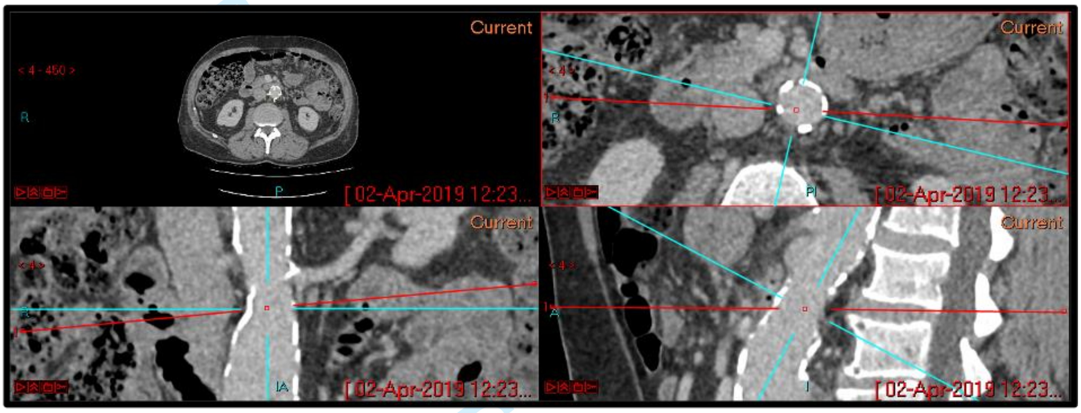

4b

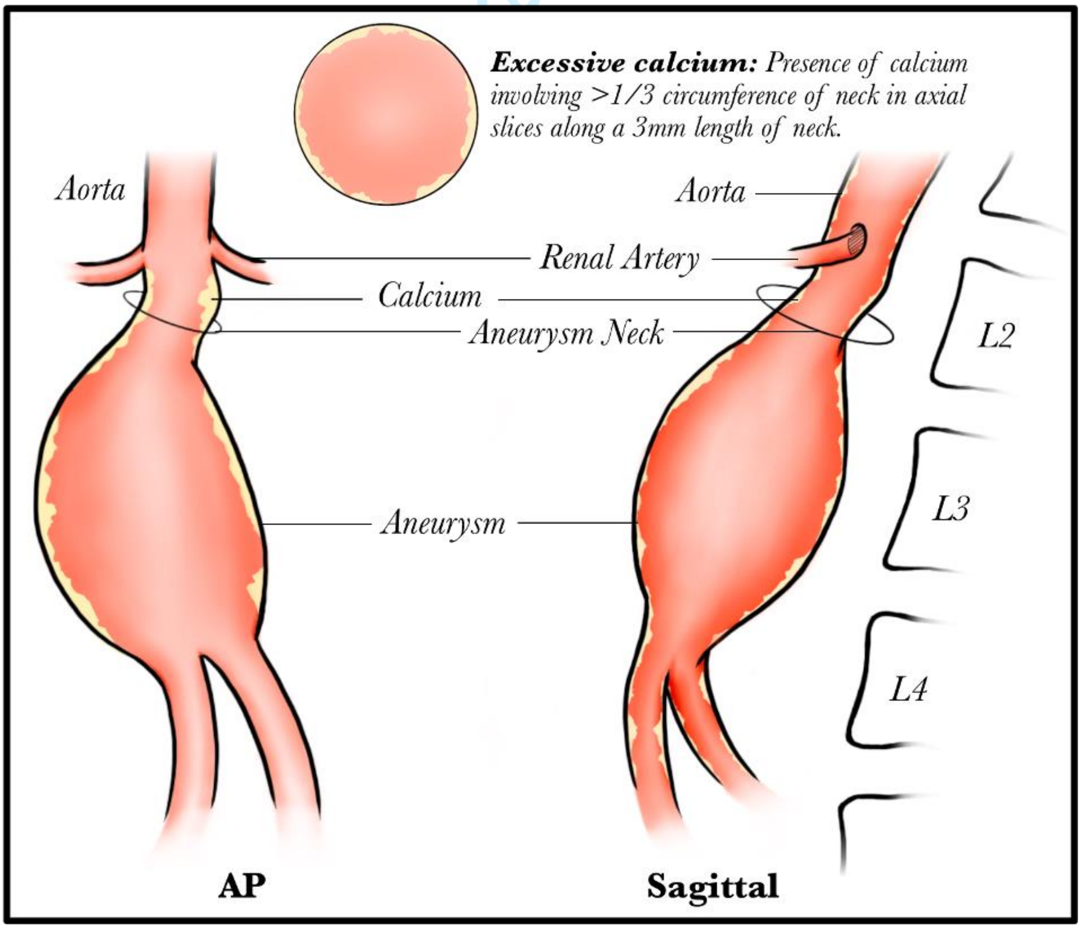

5. <u>*Neck Thrombus Load*</u>

Excessive thrombus load is defined as the presence of thrombus within the lumen of the aortic neck, lining >1/3 circumference of the neck OR filling 1/3 the surface area of the neck in axial slices along 3mm of neck length, anywhere in the first 15mm of neck (the "seal zone").

Assessment involves profiling the neck in both coronal and sagittal views, with subsequent assessment in the orthogonal axial view. Thrombus load is assessed on visual estimation of the 1/3 circumference threshold or 1/3 surface area threshold across multiple axial views of the neck (Figures 5a and 5b) and recorded as a binary outcome.

5a

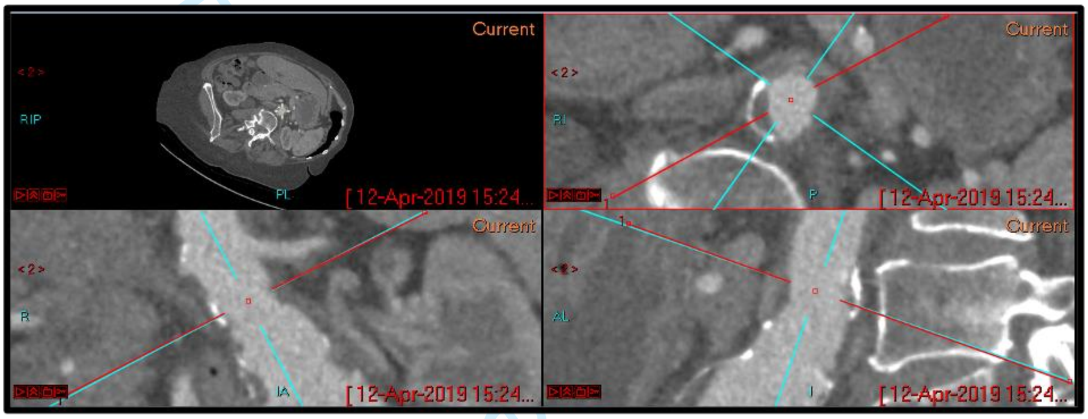

5b

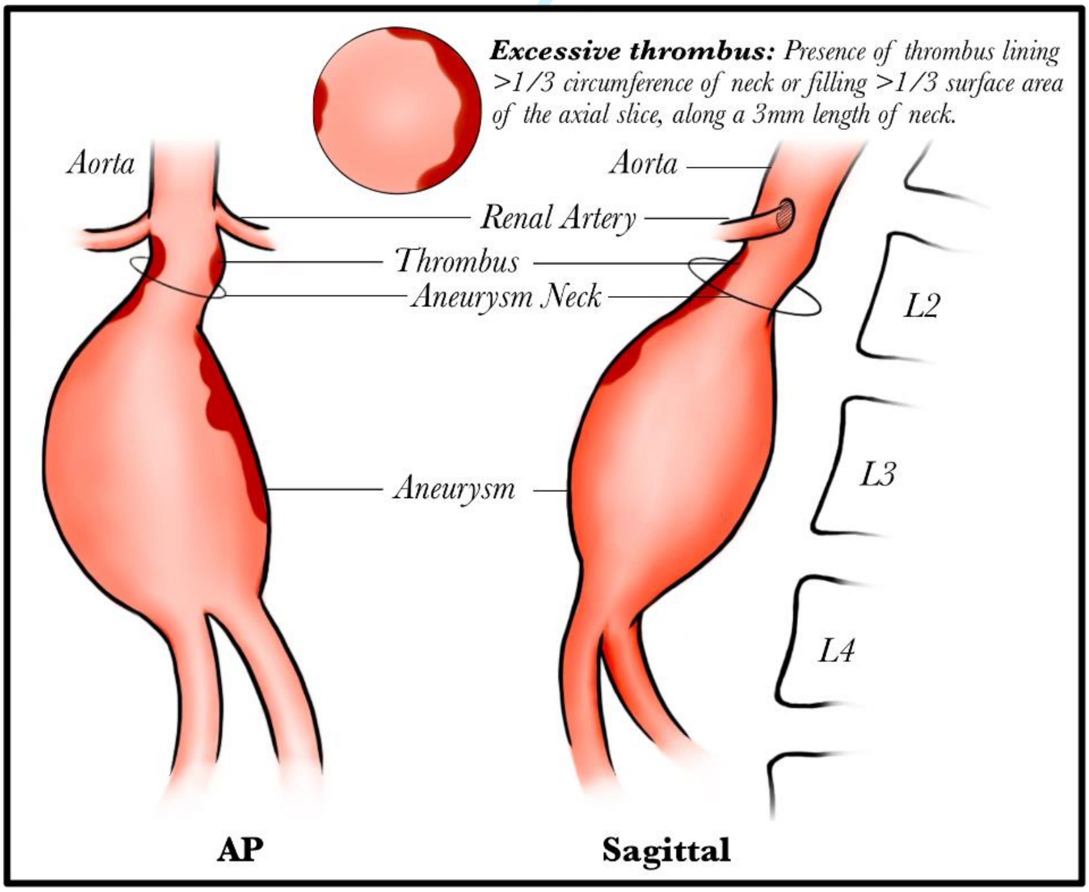

6. Neck diameters

Neck diameter is defined as the maximal outer to outer diameter (mm) in orthogonal cross section. The aneurysm neck is profiled in both coronal and sagittal views with subsequent assessment in the orthogonal axial view. Neck diameters are measured at the level of the lowermost renal artery (Figure 6a), at 5mm below this point (Figure 6b), at 10mm below this point (Figure 6c), and at 15mm below this point (Figure 6d). 15mm of neck length was chosen for diameter assessment as it is the most common length of seal zone stipulated in an EVAR stent graft's instructions for use document. The various positions along the length of the neck will be defined using the ruler function on the coronal view as seen in Figures 6a to 6e. Where the neck is <15mm in length, a neck diameter measurement will additionally be taken at the most distal point of the neck.

6a

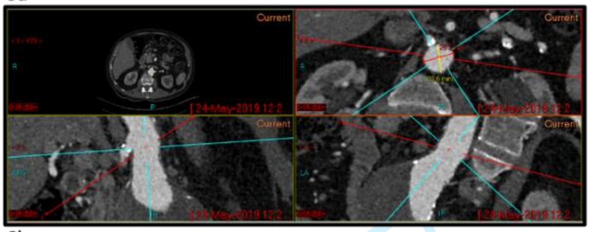

6c

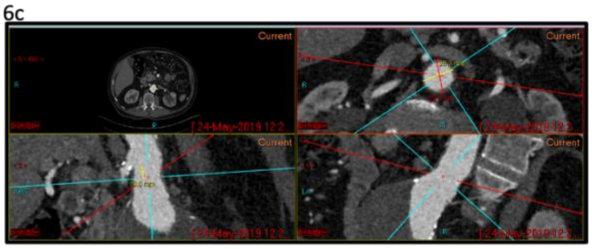

6b

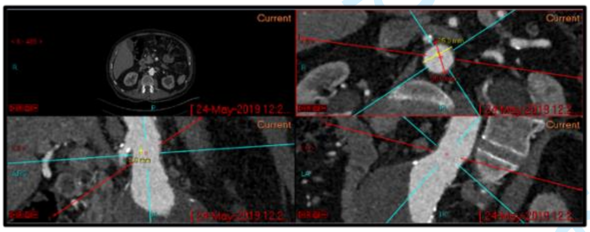

6d

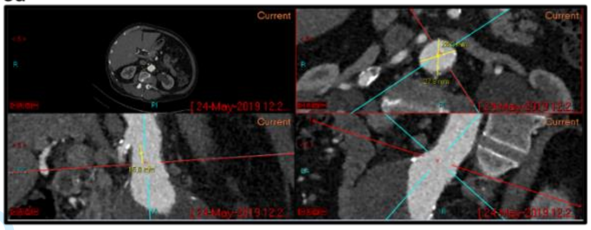

6e

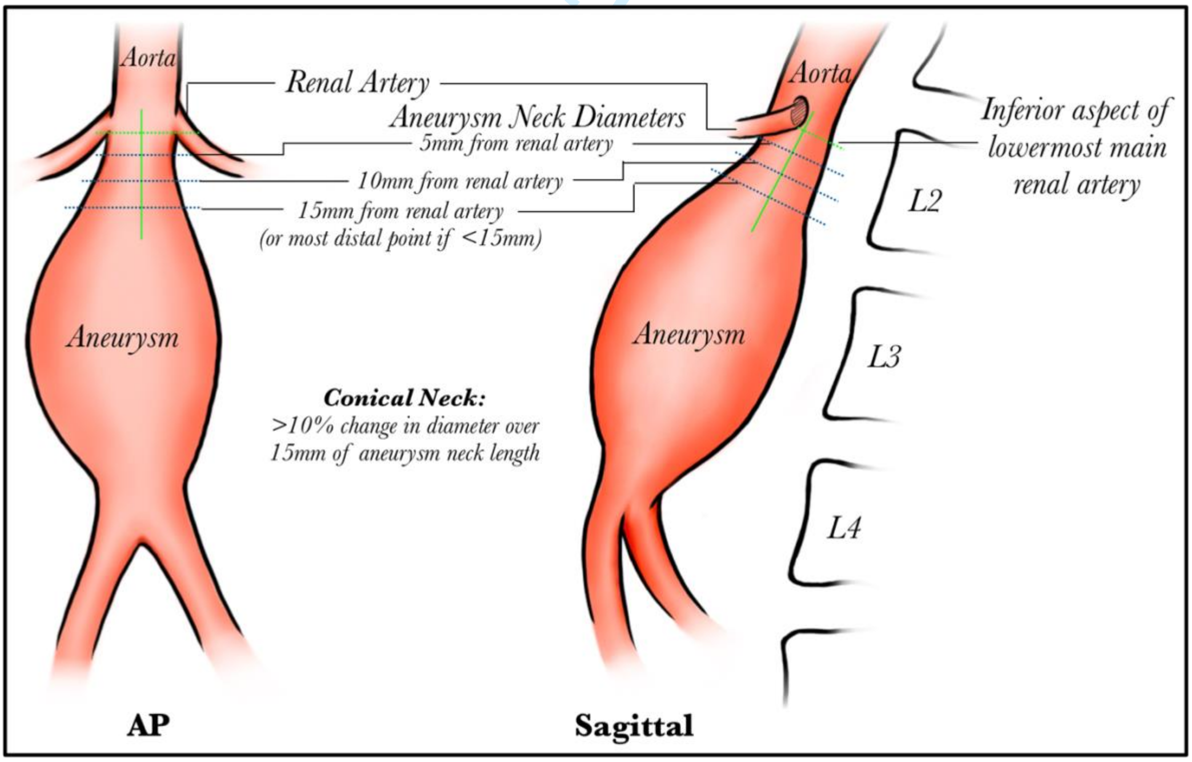

7. *α neck angle*

α neck angle refers to the angle, in degrees, between the axis of the suprarenal aorta and the axis of the infrarenal neck.

The first step of measurement is alignment of the axis of the neck in both coronal and sagittal planes. Tilting the double-oblique marker lines in the axial view in the next step will align the aorta in the coronal or sagittal views with the neck visible in the same field. The angle function is used to measure the angle between the neck and the suprarenal aorta and α neck angle is calculated by subtraction from 180 (Figures 7a and 7b).

7a

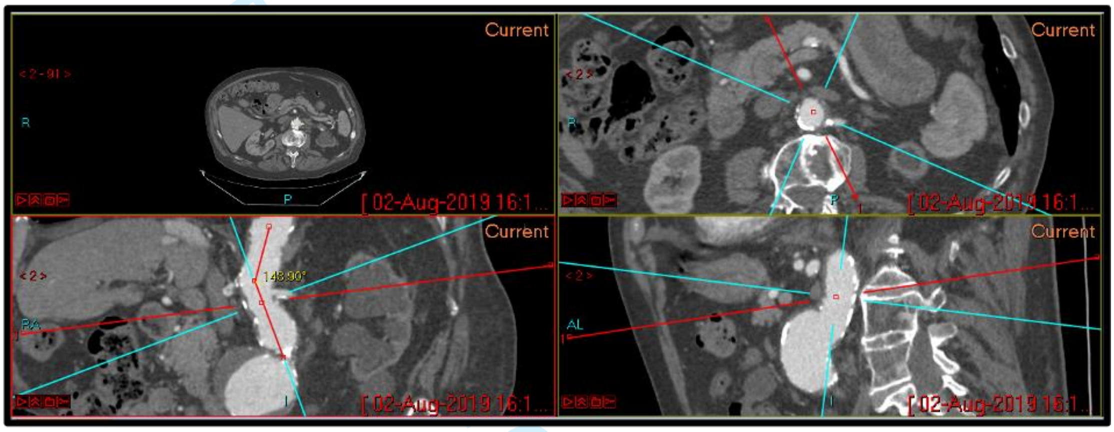

7b

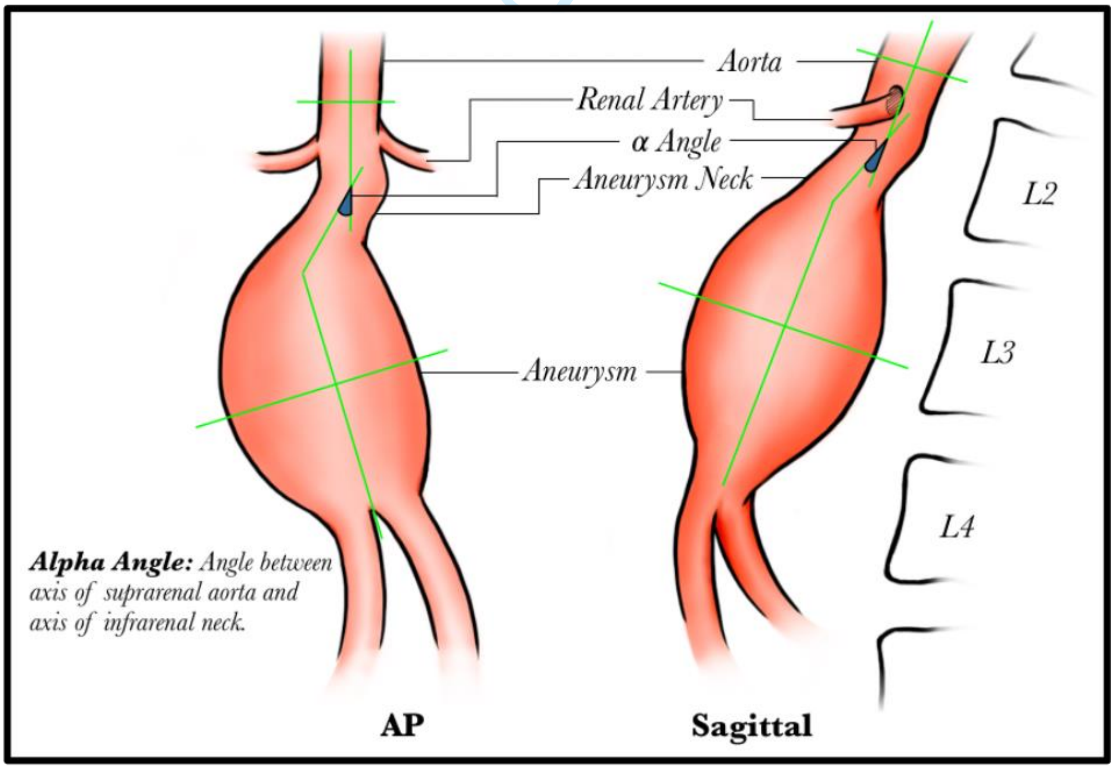

8. Non-straight necks

Non-straight neck refers to the presence of intra-neck angulation >60 degrees. This is recorded as binary outcome. If present, then a further "juxtarenal neck length" is measured, referring to the length of aneurysm neck proximal to the point of angulation but below the level of the lowermost major renal artery. This is demonstrated in Figures 8a and 8b.

Measurement of intra-neck angulation utilises the same tools and techniques as described for the aforementioned α and β neck angle measurements (Figures 3 and 7). Measurement of juxtarenal neck length utilises the same tools and techniques as described for the aforementioned total neck length measurement (Figure 2).

8a

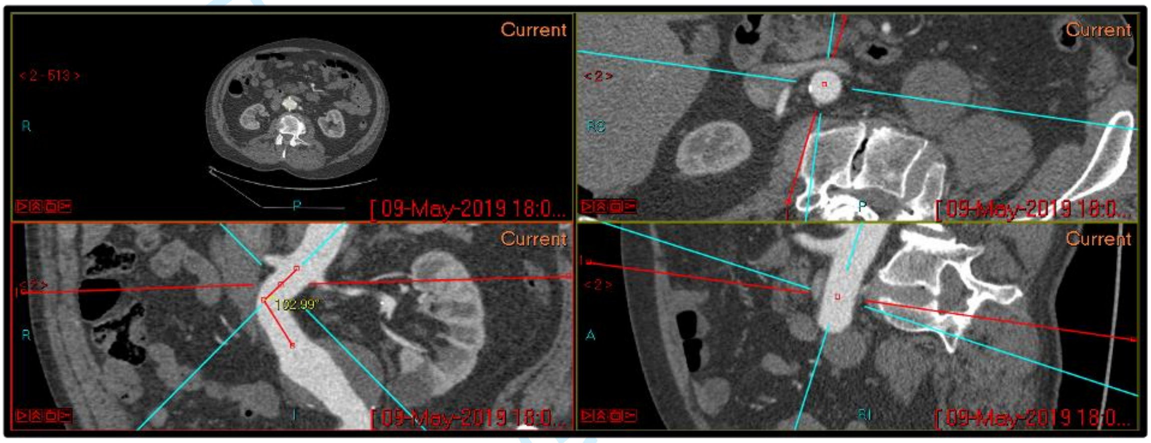

8b

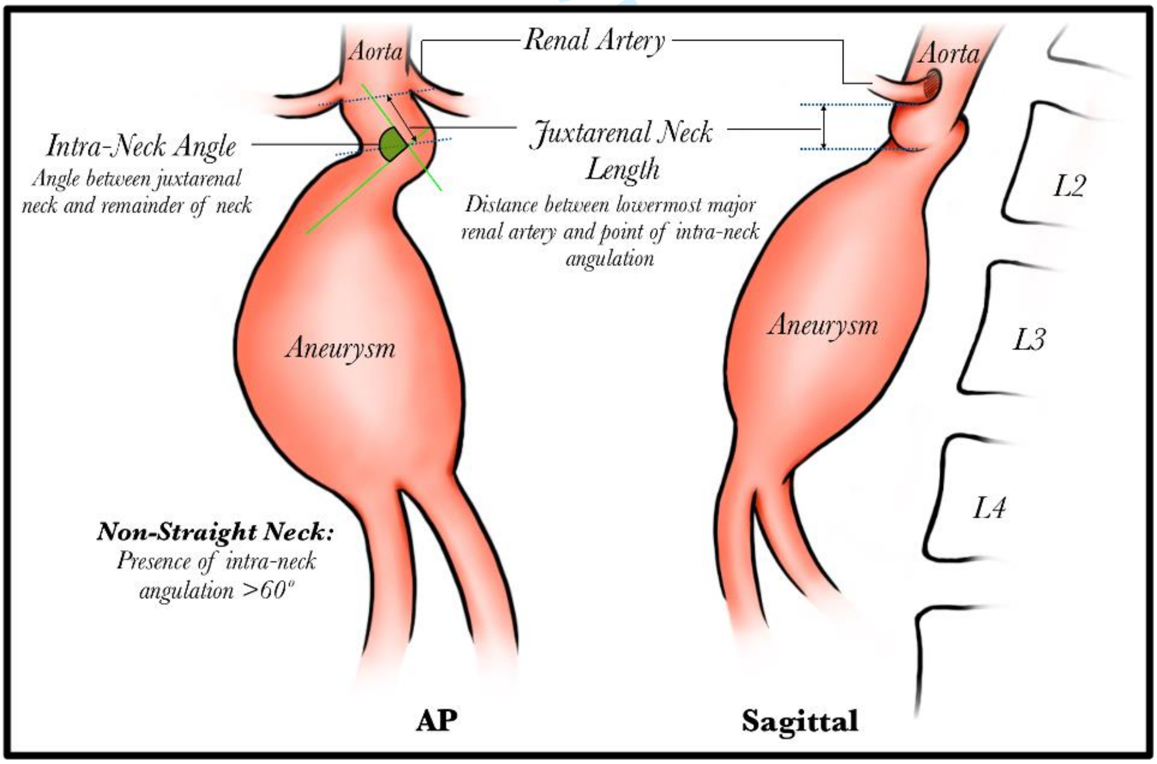

9. *Estimated Aortic Clamp Level*

This refers to the most likely position of an aortic cross clamp in the event of the aneurysm being repaired by open surgery. The levels are:

- **Infra-renal** (below the lowermost major renal artery)- if total neck length ≥5mm and free from excessive thrombus.
- **Inter-renal** (between the left and right renal arteries) - if total neck length <5mm and there is an aortic length of ≥5mm between the renal arteries.
- **Supra-renal** (between the uppermost major renal artery and the superior mesenteric artery) - if total neck length <5mm and there is no adequate inter-renal space and there is distance between uppermost renal artery and SMA ≥5mm.
- **Supra-coeliac** (above the coeliac axis) - if none of the aforementioned clamp positions are possible.

Estimating clamp level prediction requires 3 separate measurements:

1) Total neck length - this has been previously described.

2) Inter-vessel separation for renal arteries.
   This can be measured by profiling the main renal arteries on either side in the coronal view and using the ruler function to measure the distance between the two renal arteries.

3) Inter-vessel separation distance between uppermost main renal artery and SMA.
   This can be measured by profiling the renal arteries in the coronal view, marking the position of the pointer at that level so that the renal artery position can be correlated on the sagittal view. In the sagittal view, the SMA can be profiled enabling measurement of distance between the 2 with the ruler function.

For peer review only

Section 4 - The UK-COMPASS Corelab protocol – internal validation of the measurement process.

An internal validation study was performed on a subset of 70 CT scans, randomly selected from a pool of the first 500 CT scans received in the Corelab. 3 assessors of varying seniority (medical student, junior vascular trainee and senior vascular trainee) undertook independent and blinded assessment of the CT scans using the Corelab Protocol measurement process described in this manuscript. Seven measurements undergoing validity testing included maximal aneurysm diameter, total neck length, beta angle and neck diameters at 4 points along a 15mm length. Additionally, the results of the two trainees (of a seniority level reasonably expected to perform the Corelab analysis) underwent analysis to investigate potential discrepancies of patient grouping (by neck length) as a result of the measurement process.

Comparisons between Rater 1 vs Rater 2, Rater 3 vs Rater 1 and Rater 3 vs Rater 2 were performed in order to calculate and summarise the inter-rater variability. A two-way mixed effects model (each measurement done by the same set of raters, who are the only possible evaluators) was used to estimate the intra-class correlations (ICC) along with 95% confidence intervals for all 7 different measurements. Furthermore, Bland-Altman plots were generated. All statistical analysis was performed using STATAv15.1 (StataCorp. 2017. Stata Statistical Software: Release 15. College Station, TX: StataCorp LLC) An alpha level of 5% was assumed.

Inter-rater variability for the 3 raters across the 7 different measurement domains is demonstrated:

Statistic	Total neck length (units)	β Neck angle	Aneurysm diameter (units)	Diameter 0 (units)	Diameter 5 (units)	Diameter 10 (units)	Diameter 15 (units)
n	210	190	210	190	184	180	164
Mean	1.03	-0.38	0.56	0.03	0.05	0.10	-0.11
SD	6.65	10.14	2.47	1.10	1.27	1.18	2.43
Minimum	-18.0	-51.0	-4.0	-3.0	-6.0	-4.0	-19.0
Maximum	31.0	19.0	15.0	5.0	5.0	4.0	3.0

For peer review only - http://bmjopen.bmj.com/site/about/guidelines.xhtml

There was minimal inter-rater variability for all 7 domains as shown in these Bland-Altman plots:

Bland Altmann plots demonstrating excellent inter-rater agreement for measuring (a) neck length, (b) beta angle, (c) aneurysm diameter, (d) diameter of neck at level of renal artery, (e) diameter of neck 5mm below renal artery, (f) diameter of neck 10mm below renal artery, (g) diameter of neck 15mm below renal artery.
To generate the scatter plots below the mean value across the three observers (for each anatomical parameter) was calculated for each scan (x-axis). The average difference was calculated for each measurement for all three observers (y-axis).

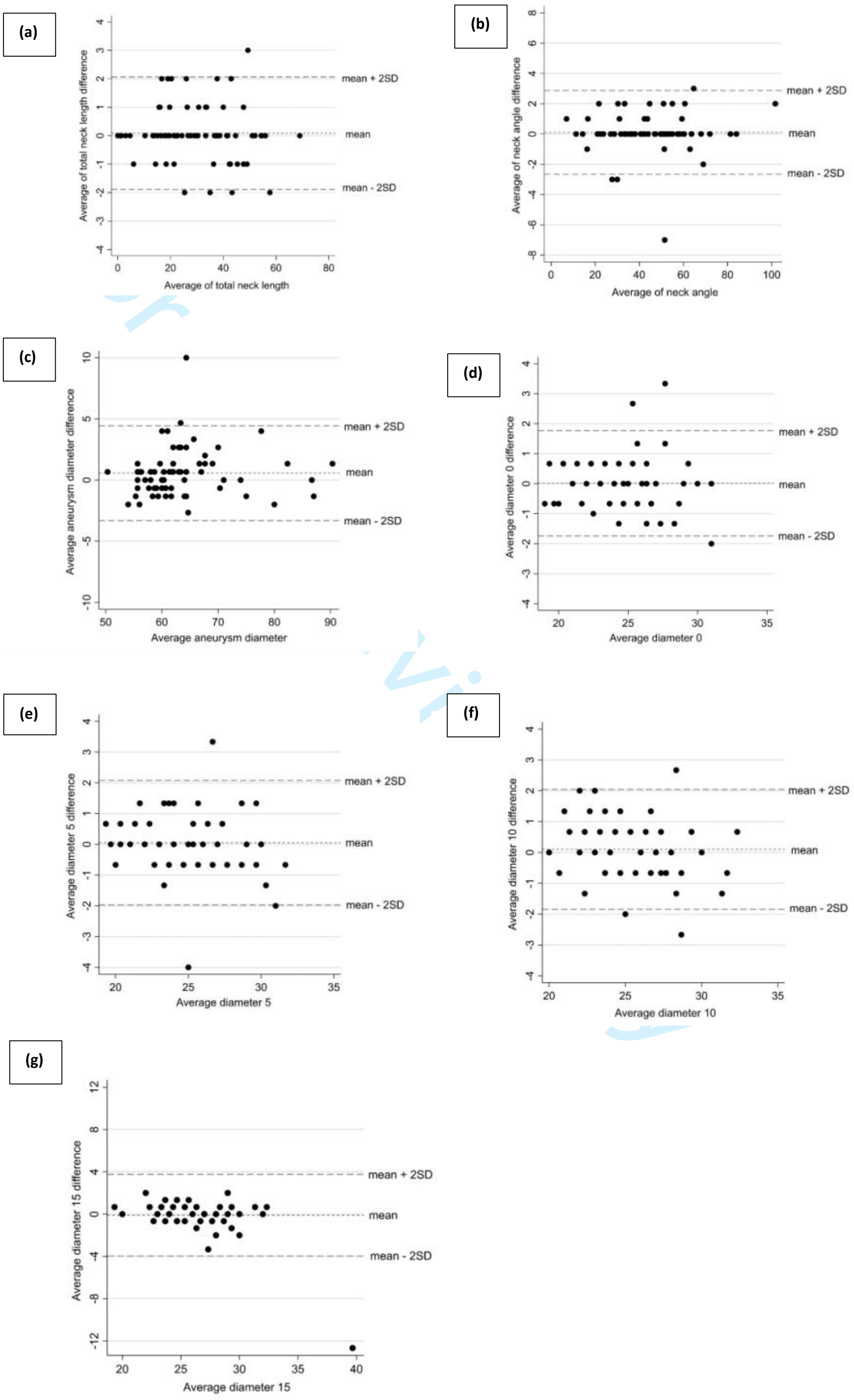

Inter-class correlation is demonstrated:

Statistic	Total neck length (units)	β Neck angle	Aneurysm diameter (units)	Diameter 0 (units)	Diameter 5 (units)	Diameter 10 (units)	Diameter 15 (units)
ICC	0.972	0.952	0.983	0.976	0.971	0.974	0.909
95% CI	0.959 – 0.982	0.927 – 0.969	0.974 – 0.989	0.964 – 0.985	0.956 – 0.982	0.960 – 0.983	0.857 – 0.945
F (df1, df2)	F(69, 138)=36.76	F(62, 124)=20.60	F(69, 138)=62.32	F(62, 124)=41.22	F(60, 120)=34.36	F(59, 118)=38.09	F(52, 104)=10.89
p-value	*<0.001*	*<0.001*	*<0.001*	*<0.001*	*<0.001*	*<0.001*	*<0.001*

A high degree of reliability was found between all measurements in all 7 domains across all 3 raters (ICC >90% for all 7 measures; p<0.001 for all).

For the grouping validation exercise of the 2 trainee measurements, a kappa statistic was calculated in order to examine inter-rater reliability. The distribution of grouping numbers for the two raters is shown:

		Rater 1				
		**1**	**2**	**3**	**4**	**Total**
**Rater 2**	**1**	*8*	*0*	*0*	*0*	8
	**2**	*0*	*2*	*0*	*0*	*2*
	**3**	*0*	*0*	*27*	*0*	*27*
	**4**	*0*	*0*	*0*	*33*	*33*
	**Total**	*8*	*2*	*27*	*33*	*70*

(Grp 1: length <5mm, Grp 2: length 4-9mm, Grp 3: length ≥10mm, Grp 4: not complex)

There was a perfect inter-rater agreement [k=1.00, se=0.0881], suggesting that any discrepancies in measurement did not correlate to discrepancies in subsequent grouping for analysis.

For peer review only - http://bmjopen.bmj.com/site/about/guidelines.xhtml

Section 5 - Microcosting exercise data collection template

Micro-costing Template for UK-COMPASS

Please complete the proforma below and provide quantities and further details in the free text boxes, in as much detail as possible e.g. brands/sizes/versions etc.

Date of procedure

1) **Operation for juxtarenal aneurysm. Please tick one:**

FEVAR	Open repair	Infrarenal EVAR	EVAR + Endoanchors	EVAR + Chimneys	Other (please specify)

2) **Pre-operative care** *(from day of diagnosis of complex aneurysm until the day of surgery)*

ITEM	Quantity	Further details
**Outpatient appt with operator**		
**Outpatient appt with other** *(Anaesthetics, another referral)*		
**Pre-assessment appt**		
**Blood tests**		
**Scans**		

For peer review only

**ECG**		
**Spirometry**		
**Transthoracic ECHO**		
**Dobutamine stress ECHO**		
**Exercise stress ECHO**		
**CPET**		
**Other tests (please specify)**		

3) **Surgery** *(from arrival to hospital until the day of discharge)*

- **Theatre utilisation**

ITEM	Quantity	Further details
**Hybrid suite (time in mins)**		
**Operating theatre (time in mins)**		

For peer review only - http://bmjopen.bmj.com/site/about/guidelines.xhtml

**IR suite (time in mins)**		

- **Personnel**

ITEM	Quantity	Further details
**Surgeon**		
**Radiologist**		
**Assistants**		
**Anaesthetist**		
**Anaesthetist Juniors**		
**ODP**		
**Scrub nurse**		
**Other theatre staff (please specify)**		

- **Medications**

ITEM	Quantity	Further details

For peer review only

**Anaesthetic Drugs**		
**Antibiotics**		
**Heparin**		
**Other intra-operative meds (please specify)**		

- **Standard Equipment**

ITEM	Quantity	Further details
**Anaesthetic equipment (endotracheal tube, cannulas, other lines etc.)**		
**Theatre trays**		
**Skin prep**		
**Drapes**		

For peer review only

**Other general theatre equipment (please specify)**		

- **Specialist Equipment**

ITEM	Quantity	Further details
**USS machine for puncture**		
**Puncture kit with standard J wire x2 (for both sides)**		
**Initial Sheaths x2 (with puncture, both sides)**		
**Large Sheaths (for device delivery and cannulation, both sides)**		
**Pigtail catheter**		
**Stiff guidewire x2 (for both sides)**		
**PROXIMAL MAIN BODY (fenestrated piece if FEVAR)**		
**Catheters for target vessels (if appropriate)**		

For peer review only

**Guidewires for target vessels (if appropriate)**		
**Sheaths for target vessels (if appropriate)**		
**Target vessel stents (for FEVAR and Chimney cases)**		
**DISTAL MAIN BODY (for FEVAR)**		
**CONTRALATERAL LIMB + ADDITIONAL LIMBS ON EITHER SIDE**		
**Coda Balloon**		
**Contrast Agent**		
**Other specialist equipment (please specify)**		

For peer review only

4) Post-operative care *(from day 1 post-op to discharge from service/death)*

- **Inpatient stay**

ITEM	Quantity	Further details
**Length of stay in bed on ward (level 1)**		

**Length of stay in Level 2 bed**		
**Length of stay in Level 3 bed**		
**Nursing care**		
**Physiotherapy care**		
**OT care**		
**Intermediate care if applicable**		

- **Outpatient care**

ITEM	Quantity	Further details
**Follow-up clinic appointments**		
**Scans**		
**Community Nursing input**		
**GP visits**		

For peer review only

BMJ Open

A risk-adjusted and anatomically stratified cohort comparison study of open surgery, endovascular techniques and medical management for juxtarenal aortic aneurysms: The UK COMPlex AneurySm Study (UK-COMPASS) - A Study Protocol.

Journal:	*BMJ Open*
Manuscript ID	bmjopen-2021-054493.R1
Article Type:	Protocol
Date Submitted by the Author:	29-Sep-2021

Complete List of Authors:	Patel, Shaneel; Liverpool University Hospitals NHS Foundation Trust, Vascular Surgery; University of Liverpool, Liverpool Centre for Cardiovascular Science<br>Ormesher, David; Liverpool University Hospitals NHS Foundation Trust, Vascular Surgery<br>Smith, Samuel; University of Liverpool, School of Medicine<br>Wong, Kitty; North Bristol NHS Trust, Vascular Surgery<br>Bevis, Paul; North Bristol NHS Trust, Vascular Surgery<br>Bicknell, Colin; Imperial College London, Department of Surgery and Cancer<br>Boyle, Jonathan ; Cambridge University Hospitals NHS Foundation Trust, Vascular Surgery<br>Brennan, John; Liverpool University Hospitals NHS Foundation Trust, Vascular Surgery<br>Campbell, Bruce; Royal Devon and Exeter Hospital, Vascular Surgery<br>Cook, Andrew; University of Southampton, Wessex Institute<br>Crosher, Alastair; Liverpool University Hospitals NHS Foundation Trust, Radiology<br>Duarte, Rui; University of Liverpool, Liverpool Reviews and Implementation Group<br>Flett, Murray; Ninewells Hospital, Vascular Surgery<br>Gamble, Carrol; University of Liverpool, Liverpool Clinical Trials Centre<br>Jackson, Richard; University of Liverpool, Liverpool Clinical Trials Centre<br>Juszczak, Maciej; University Hospitals Birmingham NHS Foundation Trust, Vascular Surgery<br>Loftus, Ian ; St George's Healthcare NHS Trust, Vascular Surgery<br>Nordon, Ian; University Hospital Southampton NHS Foundation Trust, Vascular Surgery<br>Patel, Jai; Leeds Teaching Hospitals NHS Trust, Interventional Radiology<br>Platt, Kellie; University of Liverpool, Liverpool Clinical Trials Centre<br>Psarelli, Eftychia; University of Liverpool, Liverpool Clinical Trials Centre<br>Rowlands, Peter; Liverpool University Hospitals NHS Foundation Trust, Radiology<br>Smyth, John; Manchester University NHS Foundation Trust, Vascular Surgery<br>Spachos, Theodoros ; Lancashire Teaching Hospitals NHS Foundation Trust, Vascular Surgery

	Taggart, Leigh; University of Liverpool, Liverpool Clinical Trials Centre<br>Taylor, Claire; University of Liverpool, Liverpool Clinical Trials Centre<br>Vallabhaneni, Srinivasa; Liverpool University Hospitals NHS Foundation Trust, Vascular Surgery; University of Liverpool, Liverpool Centre for Cardiovascular Science
**Primary Subject Heading**:	Surgery
Secondary Subject Heading:	Surgery
Keywords:	Vascular surgery < SURGERY, Clinical trials < THERAPEUTICS, Protocols & guidelines < HEALTH SERVICES ADMINISTRATION & MANAGEMENT, VASCULAR SURGERY

SCHOLARONE™ Manuscripts

I, the Submitting Author has the right to grant and does grant on behalf of all authors of the Work (as defined in the below author licence), an exclusive licence and/or a non-exclusive licence for contributions from authors who are: i) UK Crown employees; ii) where BMJ has agreed a CC-BY licence shall apply, and/or iii) in accordance with the terms applicable for US Federal Government officers or employees acting as part of their official duties; on a worldwide, perpetual, irrevocable, royalty-free basis to BMJ Publishing Group Ltd ("BMJ") its licensees and where the relevant Journal is co-owned by BMJ to the co-owners of the Journal, to publish the Work in this journal and any other BMJ products and to exploit all rights, as set out in our licence.

The Submitting Author accepts and understands that any supply made under these terms is made by BMJ to the Submitting Author unless you are acting as an employee on behalf of your employer or a postgraduate student of an affiliated institution which is paying any applicable article publishing charge ("APC") for Open Access articles. Where the Submitting Author wishes to make the Work available on an Open Access basis (and intends to pay the relevant APC), the terms of reuse of such Open Access shall be governed by a Creative Commons licence – details of these licences and which Creative Commons licence will apply to this Work are set out in our licence referred to above.

Other than as permitted in any relevant BMJ Author's Self Archiving Policies, I confirm this Work has not been accepted for publication elsewhere, is not being considered for publication elsewhere and does not duplicate material already published. I confirm all authors consent to publication of this Work and authorise the granting of this licence.

For peer review only

BMJ Open - Protocol submission

Title

A risk-adjusted and anatomically stratified cohort comparison study of open surgery, endovascular techniques and medical management for juxtarenal aortic aneurysms: The UK COMPlex AneurySm Study (UK-COMPASS) - A Study Protocol.

Authors

Shaneel R Patel[1,2], David C Ormesher[1], Samuel R Smith[3], Kitty H F Wong[4], Paul Bevis[4], Colin D Bicknell[5], Jonathan R Boyle[6], John A Brennan[1], Bruce Campbell[7], Andrew Cook[8], Alastair P Crosher[1], Rui V Duarte[9], Murray M Flett[10], Carrol Gamble[11], Richard J Jackson[11], Maciej T Juszczak[12], Ian M Loftus[13], Ian M Nordon[14], Jai V Patel[15], Kellie Platt[11], Eftychia-Eirini Psarelli[11], Peter C Rowlands[1], John V Smyth[16], Theodoros Spachos[17], Leigh Taggart[11], Claire Taylor[11], Srinivasa R Vallabhaneni[1,2]

1 Liverpool University Hospitals NHS Foundation Trust, United Kingdom
2 Liverpool Centre for Cardiovascular Science, University of Liverpool, United Kingdom
3 School of Medicine, University of Liverpool, United Kingdom
4 North Bristol NHS Trust, United Kingdom
5 Department of Surgery and Cancer, Imperial College London, United Kingdom
6 Cambridge University Hospitals NHS Foundation Trust, United Kingdom
7 Royal Devon and Exeter Hospital, United Kingdom
8 Wessex Institute, University of Southampton, United Kingdom
9 Department of Health Services Research, University of Liverpool, United Kingdom
10 Ninewells Hospital, Dundee, Scotland, United Kingdom
11 Liverpool Clinical Trials Centre, University of Liverpool, United Kingdom
12 University Hospitals Birmingham NHS Foundation Trust, United Kingdom
13 St George's University Hospitals NHS Foundation Trust, United Kingdom
14 University Hospital Southampton NHS Foundation Trust, United Kingdom
15 The Leeds Teaching Hospitals NHS Trust, United Kingdom
16 Manchester University NHS Foundation Trust, United Kingdom
17 Lancashire Teaching Hospitals NHS Foundation Trust, United Kingdom

Corresponding Author (and reprint requests to):
Shaneel Patel
8c Link, Royal Liverpool Hospital
Prescot Street
Liverpool
L7 8XP
shaneel.patel@nhs.net
+447824396578

For peer review only

Abstract

Introduction

In a third of all abdominal aortic aneurysms (AAA) the aneurysm neck is short (juxtarenal) or shows other adverse anatomical features rendering operations more complex, hazardous and expensive. Surgical options include open surgical repair and endovascular aneurysm repair (EVAR) techniques including Fenestrated EVAR, EVAR with adjuncts (chimneys/endoanchors) and off-label standard EVAR. The aim of The UK Complex Aneurysm Study (UK-COMPASS) is to answer the research question identified by the National Institute for Health Research Health Technology Assessment (NIHR HTA) Programme: '*What is the clinical and cost-effectiveness of strategies for the management of juxtarenal abdominal aortic aneurysm, including fenestrated endovascular repair?*'.

Methods and Analysis

UK-COMPASS is a cohort study comparing clinical and cost-effectiveness of different strategies used to manage complex AAAs with stratification of physiological fitness and anatomical complexity, with statistical correction for baseline risk and indication biases. There are two data streams. First, a stream of routinely collected data from Hospital Episode Statistics and National Vascular Registry (NVR). Preoperative computerised tomography scans of all patients who underwent elective AAA repair in England between 1st November 2017 and 31st October 2019 are subjected to Corelab analysis to accurately identify and include every complex aneurysm treated. Secondly, a site-reported data stream regarding quality-of-life and treatment costs from prospectively recruited patients across England. Site recruitment also includes patients with complex aneurysms larger than 55 mm diameter in whom an operation is deferred (Medical management). The primary outcome measure is perioperative all-cause mortality. Follow-up will be to a median of 5 years.

Ethics and Dissemination

The study has received full regulatory approvals from a Research Ethics Committee, the Confidentiality Advisory Group and the Health Research Authority. Data sharing agreements are in place with NHS Digital and the NVR. Dissemination will be via NIHR HTA reporting, peer-reviewed journals and conferences.

Trial Registration Number ISRCTN85731188

For peer review only - http://bmjopen.bmj.com/site/about/guidelines.xhtml

Strengths and Limitations of this study

- UK-COMPASS will include all patients undergoing repair of a complex AAA in England over a 24-month period in a comparative effectiveness analysis of high external validity.
- This study will involve the largest Corelab analysis of CT aortograms ever conducted to identify complex aneurysms according to predefined objective criteria pertaining to aneurysm neck anatomy.
- This study will capture every treatment method used to repair complex aneurysms.
- This study will incorporate methods of accounting for baseline risk and indication biases arising from physiological status and anatomical complexity, which will improve internal validity of comparative effectiveness analyses.
- The main limitation of the study design is a lack of random allocation, which leaves potential for unknown biases remaining uncorrected.

For peer review only

Introduction

Approximately 4000 individuals undergo elective repair of an abdominal aortic aneurysm (AAA) each year in the United Kingdom[1] with the intention of preventing premature death from aneurysm rupture. The two distinct repair methods used, open surgical repair (OSR) and endovascular aneurysm repair (EVAR), each have unique advantages and disadvantages. EVAR is safer within the perioperative period, but less durable in the years to follow compared to OSR. Treatment costs also differ between the two methods.

The complexity of aneurysm repair depends mainly upon anatomical detail relating to the segment of un-dilated aorta between renal arteries and the aneurysm, referred to as the aneurysm “neck”. Ideal aneurysm neck has normal diameter in cross section which is uniform along its length with little angulation, thrombus or calcification. Infrarenal aneurysms with at least 10-15mm length of ideal aneurysm neck are amenable to OSR with aortic occlusion clamp below the renal arteries. Such anatomy also allows standard EVAR within ‘Instructions for Use’ (IFU) defined by manufacturers of stent-grafts implanted during EVAR. Some 40-60% of aneurysms fall within this category[2-4] and there is a wealth of comparative effectiveness evidence relating to such patients[5, 6]. The remainder, whose aneurysm neck is too short (commonly called “juxtarenal”) or otherwise unsuitable for standard EVAR within IFU (on-label device use), are pragmatically referred to as having a “complex aneurysm”.

OSR of complex aneurysms involve aortic occlusion clamps at a variety of levels; below the renal arteries (infrarenal), below the superior mesenteric artery (suprarenal) or above the coeliac artery (supravisceral or supracoeliac) with increasing levels of physiological burden and technical difficulty as the clamp level moves higher. Complex aneurysms also call for advanced EVAR techniques such as fenestrated endovascular aneurysm repair (FEVAR), and EVAR with adjuncts (including chimneys and endoanchors)[7], all of which are more complex than standard EVAR. Standard EVAR outside IFU (off-label standard EVAR) is also used in routine clinical practice to treat complex aneurysms.

Repair of complex aneurysms by any technique is associated with greater risk than repair of standard aneurysms. Therefore, in patients with aneurysms larger than the conventional treatment threshold of 55mm diameter and poor physiological status, a shared decision may be made to defer operation until the risk versus benefit turns favourable towards intervention. Such ‘Medical management’ of complex aneurysms with continued surveillance beyond the 55mm size threshold has never been compared to immediate operation.

Existing evidence for different methods of repair of complex aortic aneurysms is largely in the form of case series. Most published analyses are limited by selection and indication biases. The practice of offering OSR to physiologically fitter patients and reserving EVAR techniques for the less fit patients is seldom accounted for in analyses comparing outcomes [8-10]. Furthermore, in most analyses patients are grouped by the operation techniques used rather than the detail of the anatomy being treated. A lack of precise and objective information regarding aneurysm neck anatomy, pooling of short-neck aneurysms with aneurysms without a neck as well as aneurysms of the visceral aortic segment and thoracoabdominal aortic aneurysms seriously limits the value of most published evidence[11-14]. These sources of bias,

For peer review only - http://bmjopen.bmj.com/site/about/guidelines.xhtml

confounding and heterogeneity have been consistently recognised as weaknesses in the evidence base by authors of systematic reviews[15, 16].

There is therefore a pressing need to generate comparative effectiveness evidence in this area with adequate internal and external validity. Expert opinion sought by the study funder, as well as an independently conducted workshop revealed that in the United Kingdom, a randomised controlled trial comparing OSR against FEVAR (the two principal repair methods) would be not be feasible due mainly to a lack of sufficient equipoise. Additionally, such a trial would neglect other methods of complex aneurysm repair that are widely used, including off-label EVAR and EVAR with adjuncts (such as endoanchors).

Endovascular treatments are associated with greater cost than OSR due to the cumulative costs of the stent-grafts, ancillary devices, postoperative surveillance, and reintervention. National Health Service (NHS) clinical service commissioners have noted an increasing demand for FEVAR in the absence of evidence regarding clinical effectiveness identifying which patients would benefit from this procedure and whether the substantial cost associated with the technique is an appropriate use of NHS resources. Prohibitive cost and lack of experience with FEVAR in many arterial centres has concomitantly led to a substantial use of off-label standard EVAR in complex neck anatomy, an intervention that is known to have poorer long-term results compared to on-label EVAR [17]. The National Institute for Health Research Health Technology Assessment (NIHR HTA) programme have commissioned this comparative research study in order to resolve this NHS decision problem.

The primary aim of UK-COMPASS is to answer the research question identified by the NIHR HTA Programme: '*What is the clinical and cost-effectiveness of strategies for the management of juxtarenal abdominal aortic aneurysm, including fenestrated endovascular repair?'.* The five objectives of this study are shown in Table 1.

For peer review only

Methods and Analysis

Overview

UK-COMPASS is an empirical cohort study comparing the clinical and cost-effectiveness of different management strategies used to repair complex aneurysms, incorporating statistical methods of correcting for confounding by physiological risk and anatomical complexity.

The study incorporates aspects which has been referred to by NIHR as "efficient study design", where a substantial proportion of data required for the analysis is retrieved from routinely collected sources. This reduces the cost of conducting research, facilitates inclusion of a large number of patients within a short time frame, and reflects "real world" practice. The study has two streams of data collection:

1. Routinely collected data:

This stream pertains to patients who have undergone repair of a complex aneurysm in England, over a 2-year period between 1st November 2017 and 31st October 2019. Duration of inclusion period was estimated to be 2 years commencing as soon as regulatory approvals are in place, anticipating at least 30% of aneurysms to fulfil inclusion criteria in order to provide adequate statistical precision. Health data, demographic data and health care resource use data relating to operated patients will be retrieved from Hospital Episode Statistics datasets (NHS Digital), the National Vascular Registry (HQIP), and the NHS Picture Archiving and Communication System (PACS). Median follow-up will be 5-years, until 31st October 2023.

The National Vascular Registry (NVR) is run by the Clinical Effectiveness Unit of the Royal College of Surgeons of England as a national clinical audit. Audit of NVR data revealed 95% case ascertainment and data completeness between 85.5% and 100% depending upon the data point, with a median completeness of 93.3%. Data completeness Data capture system incorporates a number of validations ensuring high level of accuracy[1]. The NVR includes a wide range of data relevant to this study, including demographics, comorbidity, preoperative assessment, intraoperative detail, postoperative complications, duration of hospital stay and critical care use for all types of AAA repair.

Hospital Episode Statistics (HES) is a data warehouse containing details of all admissions, outpatient appointments, investigations, and emergency department attendances at National Health Service (NHS) hospitals in England. These data are collected during a patient's time at hospital and is designed to enable administration as well as medical research. In addition to health and resource use data during the primary admission for aneurysm repair, follow-up data will be collected for five years after the operation to identify late complications, treatment of complications and re-interventions. Survival data is retrieved via Office for National Statistics (ONS) data linked to HES. The quality of HES data is recognised to be adequate for research and is regularly used for research purposes [18].

2. Site-reported data:

For peer review only - http://bmjopen.bmj.com/site/about/guidelines.xhtml

a. Quality of Life (QoL) data prospectively reported by consenting patients (both Operated and Medical Management) from participating arterial centres in England will supplement the routinely collected data. Recruitment will be undertaken between 1st November 2017 and 31st December 2022, with follow-up until 31st December 2023.
b. Baseline Clinical Data: Participating centres will report baseline health and demographic data for patients recruited to the QoL aspect of the study.

Primary outcome measures are perioperative mortality, late all-cause mortality and late aneurysm-related mortality.

Secondary outcome measures are perioperative complication rates (including organ system complications, infections, and complications specific to stent-grafts), perioperative secondary interventions, length of stay, intensive care usage, late complication rates (including complications specific to stent-grafts, renal failure/dialysis, aneurysm complications, infection, incisional hernia, late secondary intervention and health economic measures including incremental cost effectiveness ratio in terms of cost per incremental gain in quality adjusted life years.

All primary and secondary outcomes measures except the health economic measures will derive solely from the routinely collected data sources (HES, NVR and Imaging). The health economic outcomes will derive from a combination of routinely collected data sources (HES) and site-reported data sources (QOL collection).

All interventions that have been used in the study population will be compared as numbers allow. We anticipate methods of repair to include open surgery (OSR) with a variety of clamp levels (infrarenal, suprarenal or supravisceral), fenestrated endovascular aneurysm repair (FEVAR), off-label use of standard EVAR and EVAR with adjuncts (including chimneys and endoanchors).

Methods

1. Routinely collected data

The study population of complex aneurysms for the routinely collected data stream will be identified through anonymised Corelab analysis of pre-operative computerised tomography (CT) scans; a Corelab refers to a combination of infrastructure and methodology of image analysis according to predetermined reporting standards and definitions. Corelab CT analysis will be undertaken for all abdominal aortic aneurysms repaired in England between 1st November 2017 and 31st October 2019. These patients and details regarding their pre-operative CT scans will be identified from the Hospital Episode Statistics (HES) dataset and Diagnostic Imaging Dataset (DID) of NHS Digital. This will permit retrieval of said CT scans from the provider site to the Corelab based at the Royal Liverpool University Hospital using the Internet Exchange Portal (IEP) and PACS.

The CT scans will be analysed in the Corelab using a pre-specified protocol that was devised using a clinical consensus exercise conducted with experts from throughout the United

For peer review only - http://bmjopen.bmj.com/site/about/guidelines.xhtml

Kingdom in December 2019. The Corelab protocol is provided in full in the Online Supplementary Material file and includes information on anatomical definitions, in-depth information regarding the measurement process, as well as the results of a validation exercise. The anatomical CT-based inclusion and exclusion criteria for the routinely collected data stream are summarised in Table 2.

It is anticipated that approximately 8000 pre-operative CT scans will be analysed in the Corelab and that of these, approximately 3000 patients will meet the anatomical inclusion criteria for a complex neck. These patients are included in the analysis without further selection or randomisation. Pseudonymised baseline and postoperative follow-up data (up to 5 years) will be provided for this population of patients with complex necks, using datasets from NHS Digital and the National Vascular Registry. Additionally, intra-operative images and postoperative surveillance for the patients treated by endovascular techniques will be retrieved to the study Corelab.

Site-reported data

All arterial vascular centres in England are eligible to participate as recruitment centres for the site-reported data stream. Recruitment will be undertaken between 1st November 2017 and 31st December 2022, with follow up QoL data collected until 31st December 2023.

Patients will be prospectively identified at recruiting sites and approached for QoL follow-up using Good Clinical Practice (GCP) compliant processes. The anatomical inclusion criteria used to identify the patients mirror those of routinely collected data (see Table 2). Patients will be eligible for contribution to QoL data collection if they are due to have a complex aneurysm managed with open or endovascular surgery, as well as cases that have reached 55mm in maximal diameter and are selected to remain on extended surveillance ('Medical Management') with decision for treatment made at a later date once the aneurysm has reached a larger threshold diameter.

In addition to the anatomical exclusion criteria listed in Table 2, cases utilising surgeon-modified stent-grafts and cases in which patients are outright turned down for surgery due to co-morbidity or patient choice ('Operation Declined') will be ineligible for recruitment to QoL follow-up.

Both generic (EQ-5D-5L[19]) and aneurysm-specific (Aneurysm-DQoL[20]) QoL questionnaires will be used to collect data according to pre-specified time points for both Operated and Medically Managed patients as shown in Table 3. The data will be collected in the form of written completed questionnaires or telephone interviews, in accordance with patient preference with raw data entered and stored onto secure database by trained staff at the Liverpool Clinical Trials Centre. Change in overall QoL between baseline and subsequent intervals will be analysed for statistical significance. This information will also inform health economic analyses. A patient diary will also be completed by patients annually to provide data on health resource use following their aneurysm treatment.

Recruiting sites will also be asked to conduct a microcosting exercise in order to assess variations in endovascular consumables and local practice. A detailed cost profile will be

For peer review only - http://bmjopen.bmj.com/site/about/guidelines.xhtml

obtained for at least ten patients treated by each treatment modality: OSR, FEVAR and EVAR (+/-adjuncts). A proforma for microcosting data collection is included in the Online Supplementary Material file.

Sample size calculations

Perioperative mortality:

The primary outcome measure is perioperative death. The primary efficacy parameter is the odds ratio comparing FEVAR to both open repair and off label EVAR. For OSA, conservative estimate of 8% peri-operative death might be expected, and a reduction to 4 % could be considered clinically relevant, which is equivalent to an odds ratio of 0.48 (based on NVR 2015 report[21] and GLOBALSTAR registry[22]). This effect is slightly larger than that noted in randomised controlled trials of standard infrarenal aneurysm repair[5]. A Bonferroni adjusted two-sided alpha level of 0.025 is used. Based on these assumptions, collecting data on 2000 patients will ensure a power in excess of 80% if the allocation between treatments is relatively equal and should be preserved above 70% even if one treatment strategy is used twice as much as the other two.

Stratified comparative analysis for perioperative mortality and identification of risk factors:

Based on the best available estimates, it is anticipated that 120 events (perioperative deaths) will be available if 2000 patients are included in the study. The aim is to identify anatomical/physiological characteristics for which there is a differential effect on patient outcome depending on the treatment strategy employed. We plan to use multivariable models which include propensity scores as continuous covariates, adjusted for appropriate confounding variables and including interaction effects with treatment effect. Using the statistical rule of thumb of 10 events per variable, approximately 12 variables can be included in any single model. If, for example, a two-level factor is to be investigated, main effects and interaction effects and a covariate of propensity scores will account for 6 variables, allowing for a further inclusion of other confounding variables as required.

Long term survival; all-cause mortality:

Estimated survival rates at 1, 2 and 4 years are 90%, 80% and 70% respectively. All patients will have a median of 5 years follow-up after entering the study. It is approximated that 2000 patients should then contribute approximately 550 events (all-cause mortality after 30 days/discharge). As the study is not designed to identify a difference in long-term overall survival, no Power calculation is provided to detect some minimum clinically relevant difference. Instead, assuming the difference between two treatment arms to be measured using a log hazard ratio it is estimated that from 550 events, a standard error of approximately 0.085 will be observed. This translates to a 95% confidence interval of length of approximately 0.34. Here, a hazard ratio smaller than 0.71 or larger than 1.40 will be shown to be significant at a 5% level.

Quality of life:

We will obtain QOL data from a representative sample of patients undergoing AAA surgery during the study period. This will equate to 800 patients. We expect a 50% loss to follow-up, through noncompliance and natural attrition from death during the follow-up period. We

For peer review only - http://bmjopen.bmj.com/site/about/guidelines.xhtml

plan to obtain QOL from 300 medically managed patients. 300 patients will give a 95% confidence interval of maximum width 12.5 for the difference in mean EQ-5D-5L visual analogue scale (EQ-VAS) values between baseline and later time points based on a standard deviation equal to 20. The standard deviation was calculated after an evaluation of EQ-VAS scores across a wide range of trials.

Data analysis plan

Analyses will be carried out on an intention-to-treat basis, retaining all patients in the surgery group to which they are assigned irrespective of any procedural issues. The intervention groups will be OSR, FEVAR, EVAR +/- adjuncts (including chimneys and endoanchors). The exact treatment received will be determined by triangulation of NVR data and HES data for OSR. Patients receiving endovascular treatment will be identified from NVR data and HES data, and exact technical details of each procedure (including the use of adjuncts) will be determined through analysis of procedural images in the Corelab.

As an analysis of observational cohort data, all attempts to present a measure of efficacy free from confounding bias will be undertaken. Patients will be stratified by infrarenal neck length for analysis into 3 groups: <5mm (juxtarenal), 5-9mm (juxtarenal) and ≥10mm neck length (not juxtarenal but complex based on device-specific IFU criteria). This will serve to limit anatomical heterogeneity and bias within comparisons. Additionally, patients will be stratified by physiological risk. The British Aneurysm Repair score[23] will be used (11 clinical parameters available from National Vascular Registry data) to divide each comparison group into those that are high-risk (the highest risk quartile) and the remainder as standard risk.

Statistical analyses of the primary outcome shall follow the principle of propensity score analysis. Propensity score estimation is chosen for the analysis of the primary endpoint instead of multivariable regression techniques because it is considered the most appropriate means of accounting for selection bias at the low level of peri-operative death rates anticipated[24]. Propensity scores estimate the probability of a patient being given each treatment strategy based on baseline clinical and demographic characteristics. Patients can then be stratified based on their propensity to receive each treatment strategy and an estimate of treatment effect is then obtained, which adjusts for potential selection bias in the data.

Models to estimate the propensity for each patient shall be generated using multi-variable logistic modelling techniques using as candidate covariates only variables that influence simultaneously the treatment assignment and the outcome variable. Covariates shall be retracted to demographic/clinical data available at the point of treatment decision with the aim of producing a propensity model that satisfies the assumptions of conditional independence. The final analysis shall be carried out using stratified logistic where the strata are defined by the quintiles of the fitted propensity score values. Prior to analyses it shall be confirmed that each stratum contains at least 20 observed perioperative deaths. If not, the strata will be combined with an adjacent strata level.

For peer review only - http://bmjopen.bmj.com/site/about/guidelines.xhtml

The key efficacy parameter of interest will be an odds ratio presented with a 97.5% confidence interval. Results will be presented as a forest plot with the odds ratio within each stratum presented along with the overall effect.

Reports of HES data validation confirm high levels of completeness and accuracy. Therefore, missing data or inaccurate data is unlikely to be a significant problem. It is proposed that covariates with small amounts of missing data (≤10%) will be included on a complete case basis and covariates with a large amount of missing data (>10%) will be excluded from the analysis.

Sensitivity analyses are carried out to ensure the results are not sensitive to either the propensity model or the method of matching. With respect to the propensity model, sensitivity analyses shall be carried out by removing each covariate included in calculating the propensity score and repeating the analysis of the primary endpoints. The results of all sensitivity analyses shall be presented in a forest plot along with the overall effect from the primary analysis.

It is recognised that recruitment for site-reported quality of life data will occur either side of a cessation of elective services in the United Kingdom due to the COVID-19 pandemic in 2020/2021. There may be differences in patient behaviour and clinical practice before and after this period and we aim to investigate this further in the analysis phase. Firstly, outcome data before and after the dates of interest will be compared. Secondly, clinical practice before and after the dates of interest will be compared using data from the National Vascular Registry.

Health Economic (HE) Analysis

The UK-COMPASS study will adapt the recent NICE Health Economic Analysis model[25] which will facilitate updates of NICE Clinical Guideline 'Abdominal Aortic Aneurysm – Diagnosis and Management (NG156)'.

Our aim is to inform NICE decision-making in a timely manner. The NICE HE model has been developed from a UK NHS and Personal Social Services perspective, appraised by a NICE Committee and scrutinised during consultation, and we see this fit to be adapted without developing a de novo model.

The purpose of the health economic analysis in this study is defined in the original objectives 4 & 5. The main outputs therefore will remain A) estimation of incremental cost effectiveness ratios comparing different treatment strategies in terms of life years and quality adjusted life years gained, and B) to identify whether particular level of physiological fitness (stratified into two groups) and / or anatomical baseline characteristics (stratified into three groups) are associated with better health economic efficiency using one or other treatment strategy.

At least five-year follow-up is considered the essential minimum to capture attrition of treatment utility and accrual of treatment costs that occur from late failure/secondary intervention and postoperative surveillance.

Patient and Public Involvement (PPI)

For peer review only - http://bmjopen.bmj.com/site/about/guidelines.xhtml

The Liverpool Aneurysm Research PPI group have been actively involved in the study. The study proposal was initially presented to the group on 2nd November 2015 with subsequent follow-up.

Our PPI representatives strongly welcomed the proposed research and supported the team throughout the process of grant application and subsequently in the oversight of the study. The PPI group considered the dilemmas arising out of access to routinely collected data for this research project, including NHS number and date of birth, without explicit and specific consent from each patient. They scrutinised and approved the data management plan.

With appropriate safeguards in place, they felt that research using routinely collected data in the manner proposed here may have some advantages, by potentially reducing anxiety associated with being approached to participate in a prospective clinical study. They have also pointed out the benefits of data collected independent of physician or patient preference for a research study, thereby reducing bias. They welcomed the benefits of improved efficiency and improved generalisability of results across wider NHS.

The PPI have helped us review the lay summary and have also reviewed the grant application at all stages. Once the study has commenced, the PPI group has been expended and annual meetings are expected to monitor progress. The Trial Steering Committee has one PPI member as an independent member. The PPI group is also expected to help with dissemination of research findings by reviewing patient education material as well as supporting commissioning decisions as appropriate.

For peer review only

Ethics and Dissemination

The study protocol, informed consent form, patient information sheet and all other patient facing study documentation has received the favourable opinion of the North West – Preston Research Ethics Committee (REC) and Health Research Authority (HRA) approval.

Access to patient data in NHS Digital datasets, the NVR dataset as well as to imaging, without the need for patient consent, has been granted by the Confidentiality Advisory Group (CAG); CAG is an independent body which provides expert advice to the HRA and the Secretary of State for Health on whether applications to access confidential patient or service user information without consent should or should not be approved.

The study is managed by the Liverpool Clinical Trials Centre. The sponsor for the study is Liverpool University Hospitals NHS Foundation Trust.

Dissemination plan

The results of this study will be written up and published as an official report for the NIHR HTA. It will also be published in a peer-reviewed journal and presented at international conferences.

Authors' contributions

Roles of each author are as follows:

Shaneel Patel: Study design, planning, conduct, data acquisition, interpretation of data, writing of manuscript, editing of manuscript.
David Ormesher: Study design, planning, conduct, data acquisition, interpretation of data, writing of manuscript, editing of manuscript.
Samuel Smith: Study conduct, data acquisition, interpretation of data, editing of manuscript.
Kitty Wong: Study planning, writing of manuscript, editing of manuscript.
Paul Bevis: Study design, planning, editing of manuscript.
Colin Bicknell: Study design, planning, editing of manuscript.
Jonathan Boyle: Study design, planning, editing of manuscript.
John Brennan: Study design, planning, editing of manuscript.
Bruce Campbell: Study design, planning, editing of manuscript.
Andrew Cook: Study design, planning, editing of manuscript.
Alastair Crosher: Study conduct, data acquisition, interpretation of data, editing of manuscript.
Rui Duarte: Study design, planning, editing of manuscript.
Murray Flett: Study design, planning, interpretation of data, editing of manuscript.
Carrol Gamble: Study design, planning, interpretation of data, editing of manuscript.
Richard Jackson: Study design, planning, acquisition of data, interpretation of data, editing of manuscript.
Maciej Juszczak: Study design, planning, editing of manuscript.
Ian Loftus: Study design, planning, editing of manuscript.
Ian Nordon: Study design, planning, editing of manuscript.
Jai Patel: Study design, planning, editing of manuscript.
Kellie Platt: Study conduct, planning, editing of manuscript.

For peer review only - http://bmjopen.bmj.com/site/about/guidelines.xhtml

Eftychia-Eirini Psarelli: Study design, planning, acquisition of data, interpretation of data, editing of manuscript.
Peter Rowlands: Study design, planning, acquisition of data, editing of manuscript.
John Smyth: Study design, planning, editing of manuscript.
Theodoros Spachos: Study design, planning, editing of manuscript.
Leigh Taggart: Study conduct, planning, editing of manuscript.
Claire Taylor: Study conduct, planning, editing of manuscript.
Srinivasa Vallabhaneni: Study design, planning, conduct, data acquisition, interpretation of data, writing of manuscript, editing of manuscript.

Funding statement

This work was solely funded by the National Institute for Health Research Health Technology Assessment (NIHR HTA) programme. Grant Award ID: 15/153/02.

Competing interests statement

There are no competing interests to declare.

Registration details

ISRCTN registration: ISRCTN85731188

We would like to acknowledge the following UK-COMPASS committees and advisors:

- ***Trial Steering Committee (TSC)***
  Janet Powell (Independent chair), Florian Dick (Independent specialist), Jonathan Michaels (Independent specialist, Health Economics), Andrew Tambyraja (Independent specialist) and Paul Massey (Public and Patient Involvement representative)

- ***Independent Data Monitoring and Safety Committee (IDMSC)***
  Mike Roberts (Chair), Viktoria McMillan, Ulrike Naumann and Murray Flett

- ***Trial Management Group (TMG)***
  Barbara Arch, Colin Bicknell, James Boateng, Jonathan Boyle, Bruce Campbell, Andrew Cook, Alastair Crosher, Rui Duarte, Carol Gamble, Richard Jackson, David Ormesher, Shaneel Patel, Kellie Platt, Heather Rogers, Leigh Taggart, Claire Taylor and Syed Yusuf

- ***Patient advisers***
  Paul Massey as a Public and Patient Involvement representative for the study

Word Count *(Introduction to Dissemination Plan)*

3990

For peer review only - http://bmjopen.bmj.com/site/about/guidelines.xhtml

Table 1 – The UK-COMPASS study objectives

**Objective 1**	To compare different treatment strategies for their perioperative mortality and morbidity, corrected for confounding physiological and anatomical characteristics, in order to account for baseline risk and indication biases.
**Objective 2**	To identify whether particular physiological and/ or anatomical baseline characteristics are associated with better clinical outcomes or better health economic efficiency using one or other treatment strategy.
**Objective 3**	To compare different treatment strategies in terms of overall survival and in terms of treatment failure in the long-term follow-up (stent-graft related complications, secondary interventions, aneurysm-related mortality).
**Objective 4**	To perform cost effectiveness analyses from NHS and personal social care perspective to establish incremental cost effectiveness ratios, comparing different treatment strategies in terms of cost per incremental gain in and quality adjusted life years.
**Objective 5**	To establish the clinical and cost utility of FEVAR and alternatives, in patients who are considered physiologically unfit for OSR, and to compare these against medical managements.

For peer review only

Table 2 – CT-based anatomical inclusion and exclusion criteria for the routinely collected data stream

<table>
<tr><th>Inclusion criteria</th><th>Exclusion criteria</th></tr>
<tr><td>Aneurysm diameter ≥55mm AND neck length <10mm</td><td>Ruptured aneurysm</td></tr>
<tr><td rowspan="5">Aneurysm diameter ≥55mm AND neck length ≥10mm AND the presence of ≥1 of the following adverse neck features:
- Beta (β) angle >60°
- Conicality (>10% change in diameter along 15mm length of neck)
- Thrombus lining >1/3 circumference of the neck OR filling 1/3 the surface area of the neck along a 3mm length of neck
- Calcium load in the wall of the neck affecting >1/3 circumference of the neck along a 3mm length of neck
- Non-straight neck, defined by the presence of intra-neck angulation of >60 degrees with a juxtarenal neck length of <15mm
- Alpha (α) angle >45°</td><td>Subthreshold aneurysm (<55m)</td></tr>
<tr><td>Thoracic aortic aneurysm</td></tr>
<tr><td>Thoracoabdominal aortic aneurysm</td></tr>
<tr><td>Visceral aortic aneurysm (where aorta at level of SMA is ≥30mm)</td></tr>
<tr><td></td></tr>
</table>

For peer review only

Table 3 – Quality of Life data collection schedule

QoL tool	Pre-op	1-month post-op	3-months post-op	6-months post-op	1-year post-op	Annually up to 5 years post-op
**Operation Patients**						
EQ-5D-5L	✓	✓		✓	✓	✓
Aneurysm-DQoL	✓		✓		✓	✓
Health Diary					✓	✓
**Medically Managed Patients**						
EQ-5D-5L	✓			✓	✓	✓
Aneurysm-DQoL	✓				✓	✓
Health Diary					✓	✓

For peer review only

References

1. Waton S, Johal A, Birmpili P, Li Q, Cromwell D, Pherwani A, O'Neill R and Boyle J. National Vascular Registry: 2020 Annual Report. 2020.

2. Giménez-Gaibar A, González-Cañas E, Solanich-Valldaura T, Herranz-Pinilla C, Rioja-Artal S and Ferraz-Huguet E. Could Preoperative Neck Anatomy Influence Follow-up of EVAR? *Ann Vasc Surg*. 2017;43:127-133.

3. Marone EM, Freyrie A, Ruotolo C, Michelagnoli S, Antonello M, Speziale F, Veroux P, Gargiulo M and Gaggiano A. Expert Opinion on Hostile Neck Definition in Endovascular Treatment of Abdominal Aortic Aneurysms (a Delphi Consensus). *Ann Vasc Surg*. 2020;62:173-182.

4. Patelis ND, Malli A, Mylonas KS, Schizas D, Papa N, Economopoulos KP, Damaskos C, Moulakakis K, Katsargyris A, Georgopoulos S, Klonaris C and Liakakos T. Suitability study of current endovascular aortic repair devices based on real-life anatomic data. *Expert Rev Med Devices*. 2019;16:165-171.

5. Stather PW, Sidloff D, Dattani N, Choke E, Bown MJ and Sayers RD. Systematic review and meta-analysis of the early and late outcomes of open and endovascular repair of abdominal aortic aneurysm. *Br J Surg*. 2013;100:863-72.

6. Powell JT, Sweeting MJ, Ulug P, Blankensteijn JD, Lederle FA, Becquemin JP and Greenhalgh RM. Meta-analysis of individual-patient data from EVAR-1, DREAM, OVER and ACE trials comparing outcomes of endovascular or open repair for abdominal aortic aneurysm over 5 years. *Br J Surg*. 2017;104:166-178.

7. Buck DB, van Herwaarden JA, Schermerhorn ML and Moll FL. Endovascular treatment of abdominal aortic aneurysms. *Nat Rev Cardiol*. 2014;11:112-23.

8. O'Donnell TFX, Boitano LT, Deery SE, Schermerhorn ML, Schanzer A, Beck AW, Green RM, Takayama H and Patel VI. Open Versus Fenestrated Endovascular Repair of Complex Abdominal Aortic Aneurysms. *Ann Surg*. 2020;271:969-977.

9. Locham S, Dakour-Aridi H, Bhela J, Nejim B, Bhavana Challa A and Malas M. Thirty-Day Outcomes of Fenestrated and Chimney Endovascular Repair and Open Repair of Juxtarenal, Pararenal, and Suprarenal Abdominal Aortic Aneurysms Using National Surgical Quality Initiative Program Database (2012-2016). *Vasc Endovascular Surg*. 2019;53:189-198.

10. Michel M, Becquemin JP, Clément MC, Marzelle J, Quelen C and Durand-Zaleski I. Editor's choice - thirty day outcomes and costs of fenestrated and branched stent grafts versus open repair for complex aortic aneurysms. *Eur J Vasc Endovasc Surg*. 2015;50:189-96.

11. Bannazadeh M, Beckerman WE, Korayem AH and McKinsey JF. Two-year evaluation of fenestrated and parallel branch endografts for the treatment of juxtarenal, suprarenal, and thoracoabdominal aneurysms at a single institution. *J Vasc Surg*. 2020;71:15-22.

12. O'Donnell TFX, Patel VI, Deery SE, Li C, Swerdlow NJ, Liang P, Beck AW and Schermerhorn ML. The state of complex endovascular abdominal aortic aneurysm repairs in the Vascular Quality Initiative. *J Vasc Surg*. 2019;70:369-380.

13. Tsilimparis N, Perez S, Dayama A and Ricotta JJ, 2nd. Endovascular repair with fenestrated-branched stent grafts improves 30-day outcomes for complex aortic aneurysms compared with open repair. *Ann Vasc Surg*. 2013;27:267-73.

14. Sala-Almonacil VA, Zaragozá-García JM, Ramírez-Montoya M, Molina-Nácher V, Martínez-Perelló I and Gómez-Palonés FJ. Fenestrated and chimney endovascular aneurysm repair versus open surgery for complex abdominal aortic aneurysms. *J Cardiovasc Surg (Torino)*. 2017;58:801-813.

For peer review only - http://bmjopen.bmj.com/site/about/guidelines.xhtml

15. Nordon IM, Hinchliffe RJ, Holt PJ, Loftus IM and Thompson MM. Modern treatment of juxtarenal abdominal aortic aneurysms with fenestrated endografting and open repair--a systematic review. *Eur J Vasc Endovasc Surg*. 2009;38:35-41.
16. Rao R, Lane TR, Franklin IJ and Davies AH. Open repair versus fenestrated endovascular aneurysm repair of juxtarenal aneurysms. *J Vasc Surg*. 2015;61:242-55.
17. Antoniou GA, Georgiadis GS, Antoniou SA, Kuhan G and Murray D. A meta-analysis of outcomes of endovascular abdominal aortic aneurysm repair in patients with hostile and friendly neck anatomy. *J Vasc Surg*. 2013;57:527-38.
18. Herbert A, Wijlaars L, Zylbersztejn A, Cromwell D and Hardelid P. Data Resource Profile: Hospital Episode Statistics Admitted Patient Care (HES APC). *Int J Epidemiol*. 2017;46:1093-1093i.
19. Herdman M, Gudex C, Lloyd A, Janssen M, Kind P, Parkin D, Bonsel G and Badia X. Development and preliminary testing of the new five-level version of EQ-5D (EQ-5D-5L). *Qual Life Res*. 2011;20:1727-36.
20. Romaine J, Peach G, Thompson M, Hinchliffe RJ and Bradley C. Psychometric validation of three new condition-specific questionnaires to assess quality of life, symptoms and treatment satisfaction of patients with aortic aneurysm. *J Patient Rep Outcomes*. 2019;3:29.
21. National Vascular Registry: 2015 Annual Report. 2015.
22. Early results of fenestrated endovascular repair of juxtarenal aortic aneurysms in the United Kingdom. *Circulation*. 2012;125:2707-15.
23. Grant SW, Hickey GL, Grayson AD, Mitchell DC and McCollum CN. National risk prediction model for elective abdominal aortic aneurysm repair. *Br J Surg*. 2013;100:645-53.
24. Cepeda MS, Boston R, Farrar JT and Strom BL. Comparison of logistic regression versus propensity score when the number of events is low and there are multiple confounders. *Am J Epidemiol*. 2003;158:280-7.
25. Béliveau A, Boyne DJ, Slater J, Brenner D and Arora P. BUGSnet: an R package to facilitate the conduct and reporting of Bayesian network Meta-analyses. *BMC Med Res Methodol*. 2019;19:196.

For peer review only

Online Supplementary Material for UK-COMPASS protocol

Contents

Section	Content	Pages
Section 1	The UK-COMPASS Corelab protocol – details on CT scan management, quality control, protocol development and clinical consensus decision-making.	2-3
Section 2	Definitions used in the Corelab protocol measurement process (Table)	4
Section 3	The UK-COMPASS Corelab protocol – the measurement process	5-13
Section 4	The UK-COMPASS Corelab protocol – internal validation of the measurement process.	14-16
Section 5	Microcosting exercise data collection template to be used in the site-recruited data stream.	17-23

For peer review only

Section 1 - The UK-COMPASS Corelab protocol – details on Computerised Tomography (CT) scan management, quality control and protocol development.

UK-COMPASS's Corelab is a fully digital and paper-free facility in keeping with NIHR's carbon reduction guidelines.

CT scan management

CT scan management involves the retrieval, pseudonymisation and upload of CT scans for Corelab analysis in line with regulatory approvals. It is performed by the 'Research PACS Clerk' who is a trained member of the UK-COMPASS team and an employee of the host NHS trust. The process is conducted in compliance with the principles of The General Data Protection Regulation (GDPR), tailored by the Data Protection Act 2018, with appropriate training for all members of the study team.

The date and location of the most recent CT scan of the abdominal aorta, prior to the date of the patient's operation, is identified from Hospital Episodes Statistics (administrative data warehouse containing details of all admissions, outpatient appointments and emergency department attendances at NHS hospitals in England). This CT scan Digital Imaging and Communications in Medicine (DICOM) data is retrieved via the NHS Picture Archiving Communication System (PACS) utilising NHS number, date of birth and the date of specific CT scan in question, as provided by NHS Digital under a data sharing agreement. Legal basis was secured to access patient data without consent through the Secretary of State's Section 251 approval with Confidentiality Advisory Group (CAG) recommendation. Upon receipt, the scan data is irreversibly pseudonymised. This process was initially carried out using Carestream Vue PACS v11.4.1.1011 software (Carestream Health, Rochester, NY, USA), but later Osirix MD (Osirix, Geneva, Switzerland) was used for faster processing. This results in removal of all identifiers from the DICOM dataset such as name, date of birth, NHS and local hospital ID numbers. These data are replaced with a unique Study ID number which acts as pseudonym. It is not possible for the study team to re-identify any patient from this point.

The pseudonymised scans are uploaded onto a research section of the local PACS server, entitled the "Vendor Neutral Archive" (VNA), accessible via a two-stage authentication process and only to members of the UK-COMPASS team approved for Corelab analysis. Analysis of these scans is therefore blinded. It is possible to see the Study ID and date of scan acquisition when a CT is loaded for analysis, but not possible to re-identify a patient.

Quality Control of Management process

The Research PACS Clerk removes identifiable data from a retrieved CT scan using the PACS computer programme. Successful completion of pseudonymisation is verified for each scan in a 3-step quality control process. **Step 1:** The PACS clerk will perform complete anonymisation at DICOM data level upon receipt of the CT scan. **Step 2**: CT image set placed into an upload portal and success of anonymisation is verified. **Step 3:** The PACS clerk will retrieve the CT image set from the VNA into the Carestream viewing client and conduct a final check.

As of June 2021, of the 8613 scans that had been measured in the Corelab, no scans were found to have failed anonymisation confirming robustness of the quality control process.

For peer review only - http://bmjopen.bmj.com/site/about/guidelines.xhtml

Development of measurement protocol

The Corelab protocol was initially devised by consensus between three researchers (Chief Investigator: SV and two clinical research fellows: SP and DO) following literature review regarding anatomical features of complexity with respect to the neck of abdominal aortic aneurysms. With paucity of information regarding Corelab set-up in the published literature, content was determined by consensus and subsequent approval by members of the UK-COMPASS Clinical Consensus Group.

Clinical consensus exercise

A Clinical Consensus Group made up of Vascular Surgeons and relevant multidisciplinary experts from the U.K. met (December 2019) to decide anatomical definitions and a priori grouping for analysis. Corelab procedures were provided in advance. Suggestions and discussion were undertaken in a focus group format with opportunity for all to express opinion before reaching consensus through voting.

It was decided that neck length is the morphological feature of most interest with regards to complex aneurysms. Grouping by neck length in mm would provide clinical relevance to the findings and limit anatomical heterogeneity. Furthermore, the degree to which an infrarenal neck is short will influence which specific endovascular techniques are likely to be employed as well as whether the aortic clamp during OSR can be placed below the renal arteries. Therefore, the following primary anatomical stratification was recommended:

Group 1: neck length 4mm or shorter
Group 2: neck length 5-9mm
Group 3: neck length ≥10mm AND unsuitable for standard EVAR within IFU

Within group 3, it is appreciated that there will be a variety of adverse neck features present other than neck length (conicality, angulation, thrombus burden and calcification). It may be possible to separate these depending on the numbers included but the a priori analysis plan will be to group these cases.

Large neck diameter is an adverse neck feature. It was felt that 30mm should be the distinction between normal aortic neck and aneurysmal aorta. Therefore, it is recognised that there may be cases of uniform necks with adequate length but large diameter (≥30mm) in Group 1. These can be removed and interrogated as a subgroup analysis. There will therefore be no large diameter necks in Group 3.

For peer review only

Section 2 - Definitions used in the Corelab protocol measurement process (Table)

Measurement terminology	Unit of measurement	Definition
**Total neck length**	Millimetres (mm)	Distance between:<br>a) Lowermost major renal artery and,<br><br>b) - Point of abrupt change in calibre *(if the neck is parallel sided and <30mm diameter)* OR,<br>- where the aorta reached 30mm in diameter
**Neck diameter**	Millimetres (mm)	Orthogonal diameter of infrarenal neck at specified distances (0mm, 5mm, 10mm and 15mm) below the origin of the lowermost major renal artery (outer wall to outer wall diameter)
**α neck angle**	Degrees (°)	Angle between axis of suprarenal aorta and axis of infrarenal neck
**β neck angle**	Degrees (°)	Angle between axis of infrarenal neck and axis of aneurysm
**Non-straight neck**	Binary	Presence of intra-neck angulation >60°
**Intra-neck angle** *(when non-straight neck present)*	Degrees (°)	Angle between juxtarenal neck and remainder of neck
**Juxtarenal neck length** *(when non-straight neck present)*	Millimetres (mm)	Distance between lowermost major renal artery and point of intra-neck angulation
**Aneurysm Diameter**	Millimetres (mm)	Maximum orthogonal diameter of aneurysm (outer-to-outer)
**Excessive thrombus**	Binary	Presence of thrombus lining >1/3 circumference of neck or filling >1/3 surface area of the axial slice, along a 3mm length of neck
**Excessive calcification**	Binary	Presence of calcium involving >1/3 circumference of neck in axial slices along a 3mm length of neck

For peer review only - http://bmjopen.bmj.com/site/about/guidelines.xhtml

Section 3 - The UK-COMPASS Corelab protocol – the measurement process

Image analysis was initially performed using the multi-planar reconstruction function of Carestream Vue PACS v11.4.1.1011 software (Carestream Health, Rochester, NY, USA). After completion of measurement on approximately 3000 scans, the host site installed an updated version of the same programme (v12.2.2.1025) for the measurement process of the remainder. h

CT series of the abdominal aorta with arterial phase contrast were loaded in double-oblique multiplanar reconstructed (MPR) format. This permitted visualisation of the aneurysm/aneurysm neck in axial, sagittal and coronal views. Contrast windowing and magnification were considered baseline adjustments prior to commencement of the measurement process, which is detailed below for specific measures:

1. *Aneurysm diameter*

Both the coronal and sagittal planes are aligned with the long axis of the aneurysm body (not the flow lumen) using double oblique adjustment. This allows orthogonal axial view of the aneurysm at its largest point. A ruler function is used to measure the maximal (outer to outer) diameter of the aneurysm in the axial plane (Figures 1a and 1b).

1a

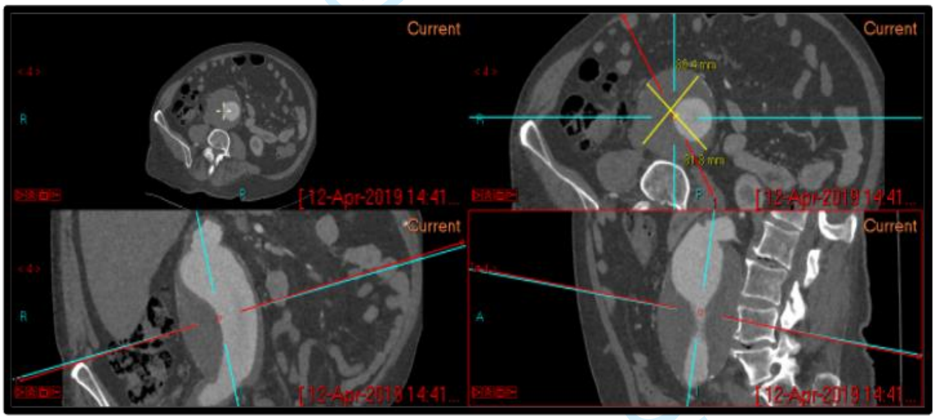

1b

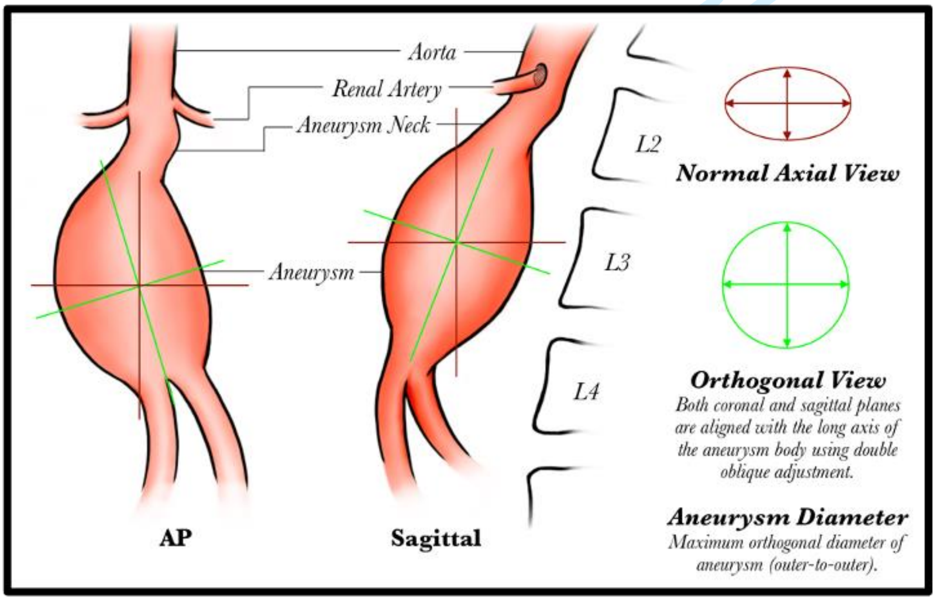

2. <u>Total neck length</u>

Total neck length is defined as the distance between the inferior aspect of the lowermost main renal artery and the superior aspect of the aneurysm.
The superior aspect of the aneurysm is defined as:

- The transition between normal aorta and aneurysm, where the aneurysm commences abruptly (if the neck is parallel sided and <30mm diam), OR
- the point at which the neck diameter becomes 30mm in cases where the transition to aneurysm is not abrupt.

Long axis of the aneurysm neck is aligned to both coronal and sagittal planes and the distal margin of the lower most renal artery is profiled by aligning double-oblique planes to the renal artery ostium in the axial view. A ruler function is used to measure the neck length in both coronal and sagittal views (Figures 2a and 2b).

2a

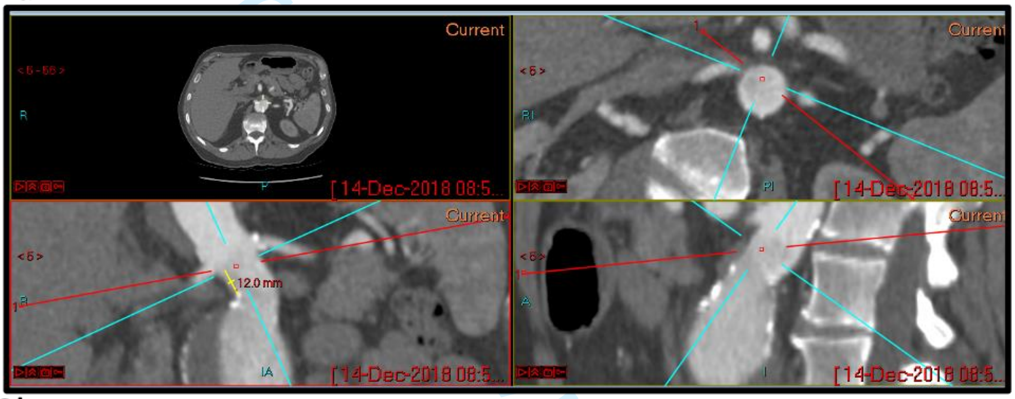

2b

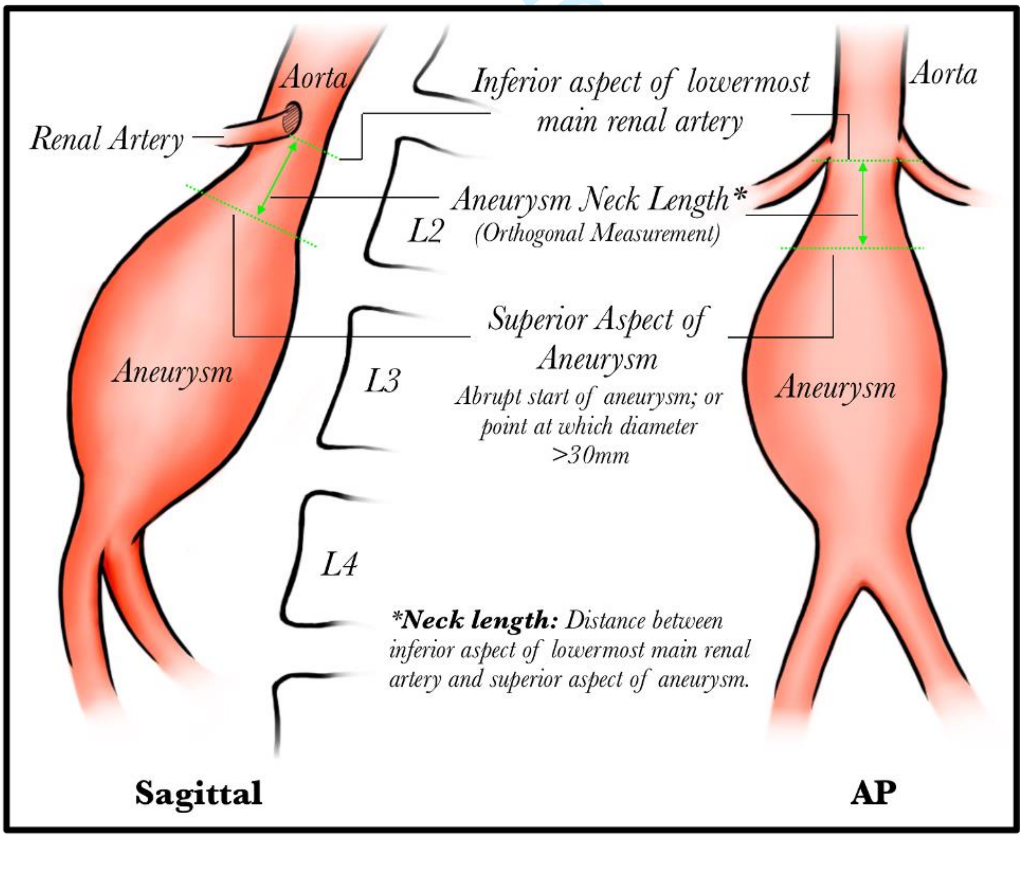

3. β neck angle

Beta (β) neck angle refers to the angle between the axis of the neck and the axis of the aneurysm, measured in degrees.

The first step is to align both coronal and sagittal planes to the axis of the aneurysm neck. Tilting the double-oblique marker lines in the axial view will permit visualisation of the aneurysm axis in the coronal or sagittal views. The largest possible angle between the axis of the neck and the axis of the aneurysm is measured and subtracted from 180 to calculate the β neck angle (Figures 3a and 3b).

3a

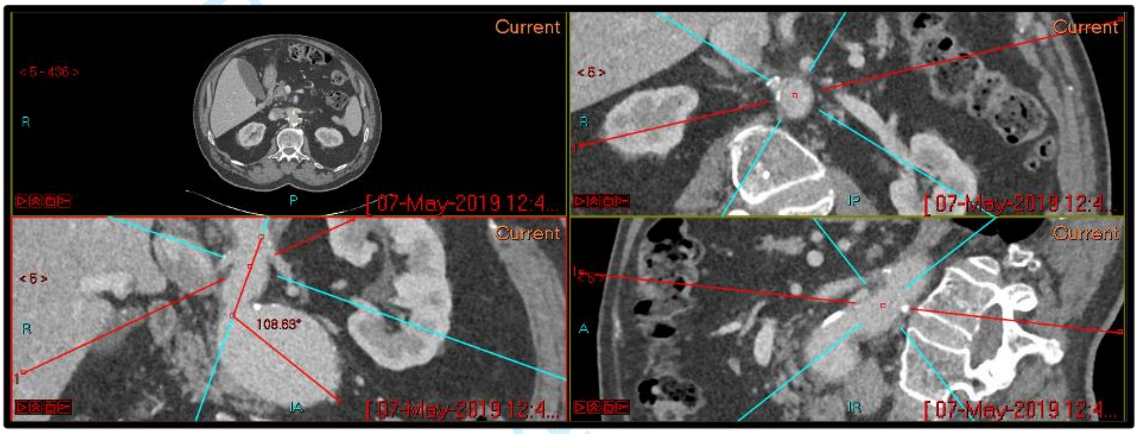

3b

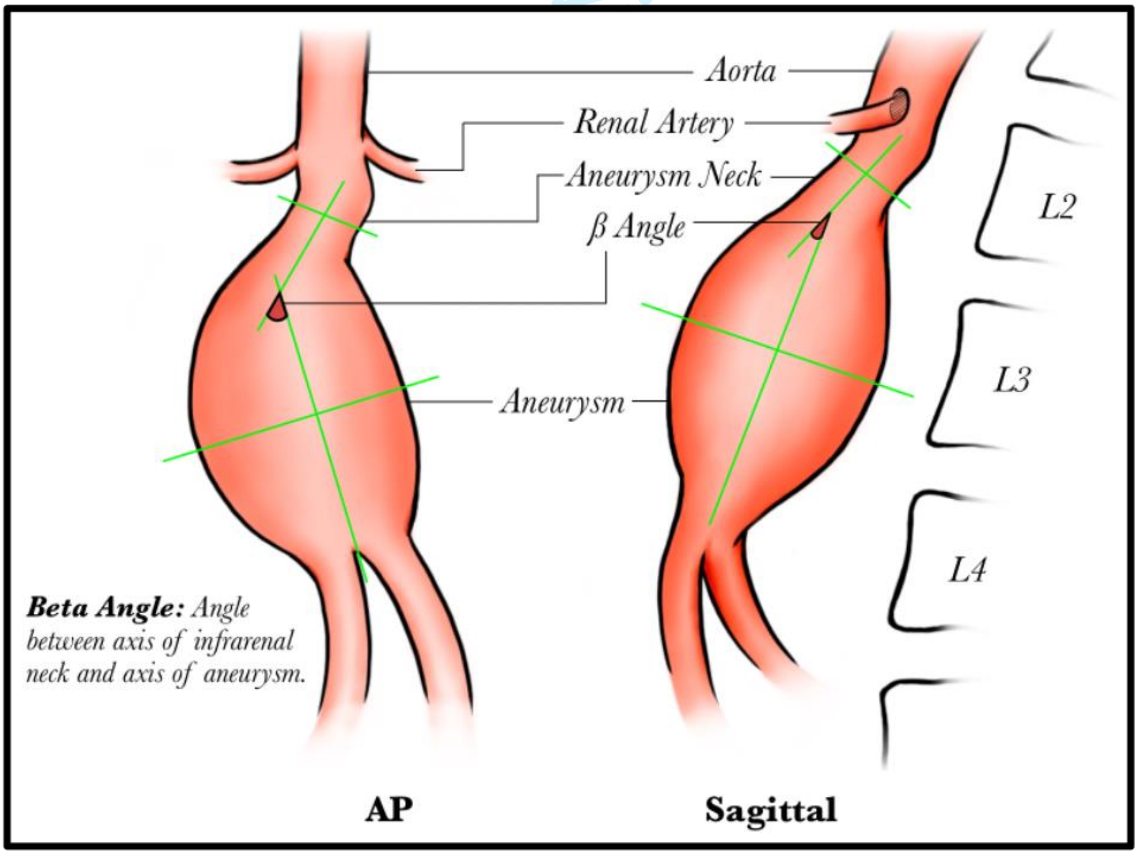

4. <u>*Neck Calcification*</u>

Excessive calcium load is defined as the presence of calcification affecting >1/3 circumference of the neck along a 3mm length of neck, anywhere in the first 15mm of neck (the "seal zone"), either as 1 continuous "plate" of calcium, multiple plaques or as speckled coverage.

Measurement involves profiling the neck in both coronal and sagittal views, with subsequent assessment in the orthogonal axial over 3mm lengths of neck (Figures 4a and 4b). Presence of excess calcification is determined based on a visual estimation of the 1/3 circumference threshold and recorded as binary outcome.

4a

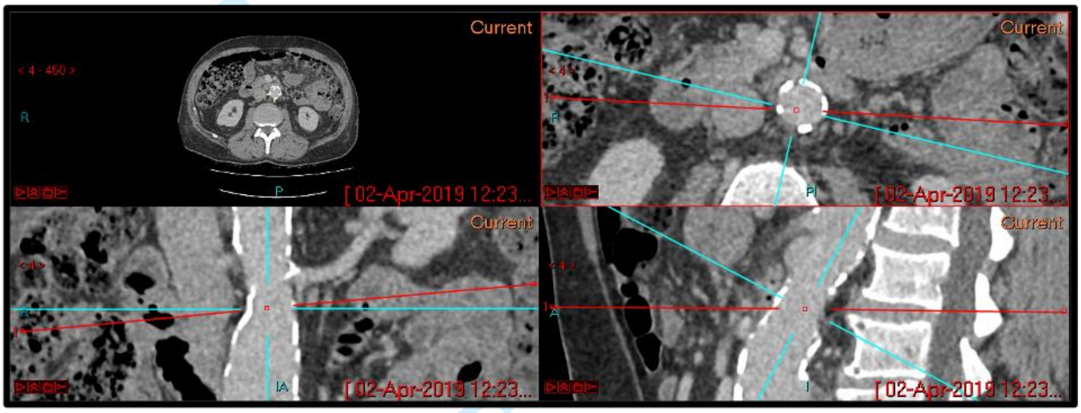

4b

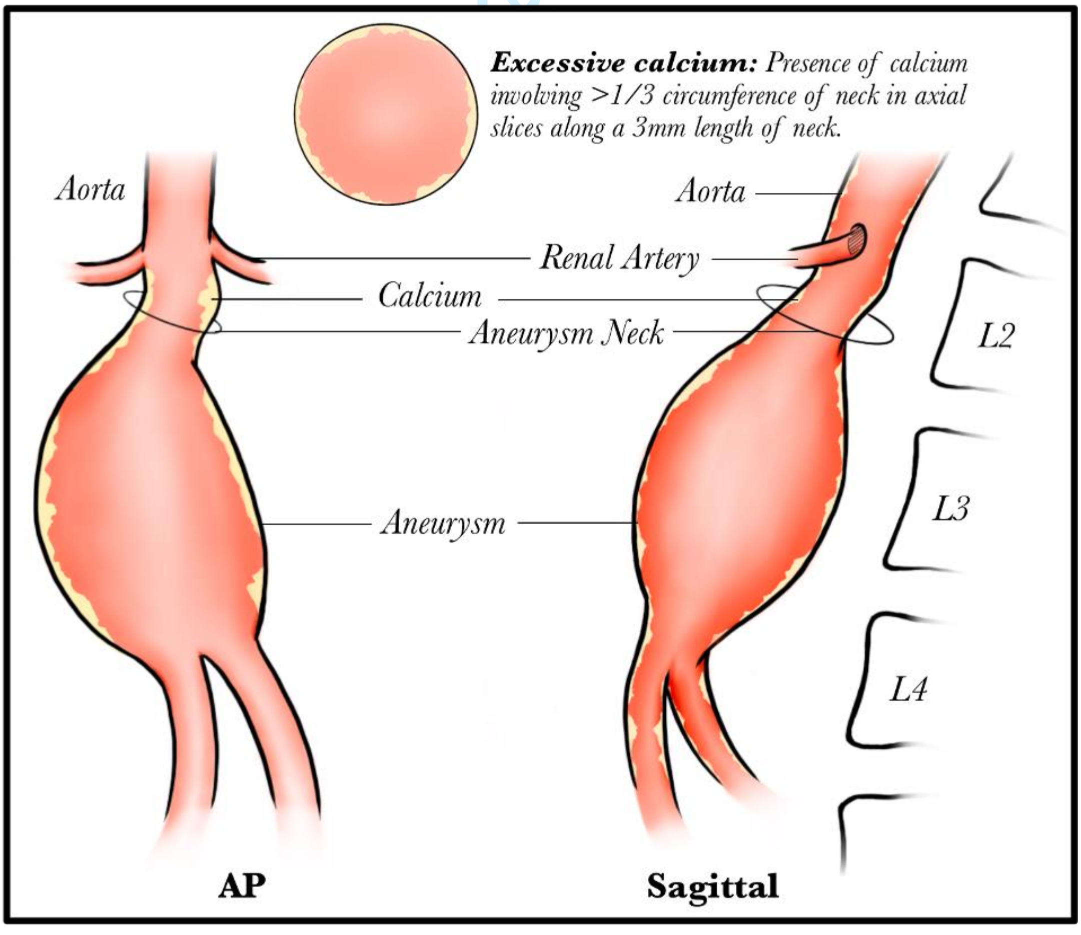

5. <u>*Neck Thrombus Load*</u>

Excessive thrombus load is defined as the presence of thrombus within the lumen of the aortic neck, lining >1/3 circumference of the neck OR filling 1/3 the surface area of the neck in axial slices along 3mm of neck length, anywhere in the first 15mm of neck (the "seal zone").

Assessment involves profiling the neck in both coronal and sagittal views, with subsequent assessment in the orthogonal axial view. Thrombus load is assessed on visual estimation of the 1/3 circumference threshold or 1/3 surface area threshold across multiple axial views of the neck (Figures 5a and 5b) and recorded as a binary outcome.

5a

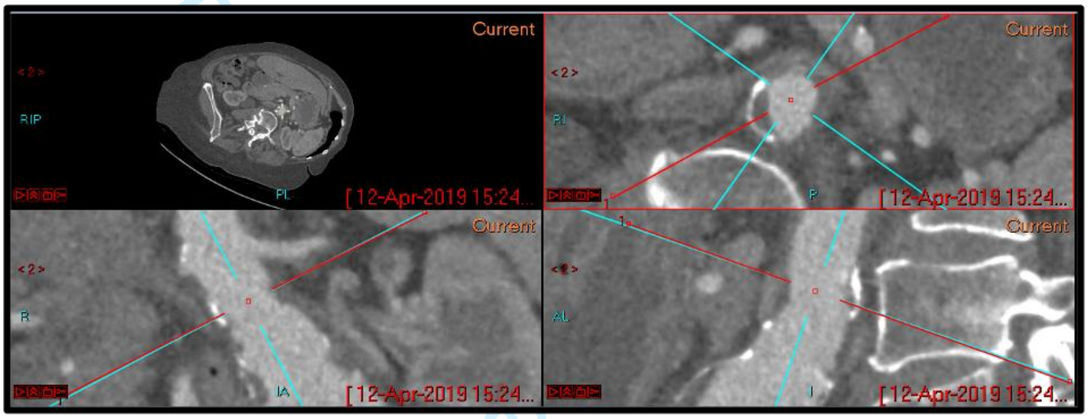

5b

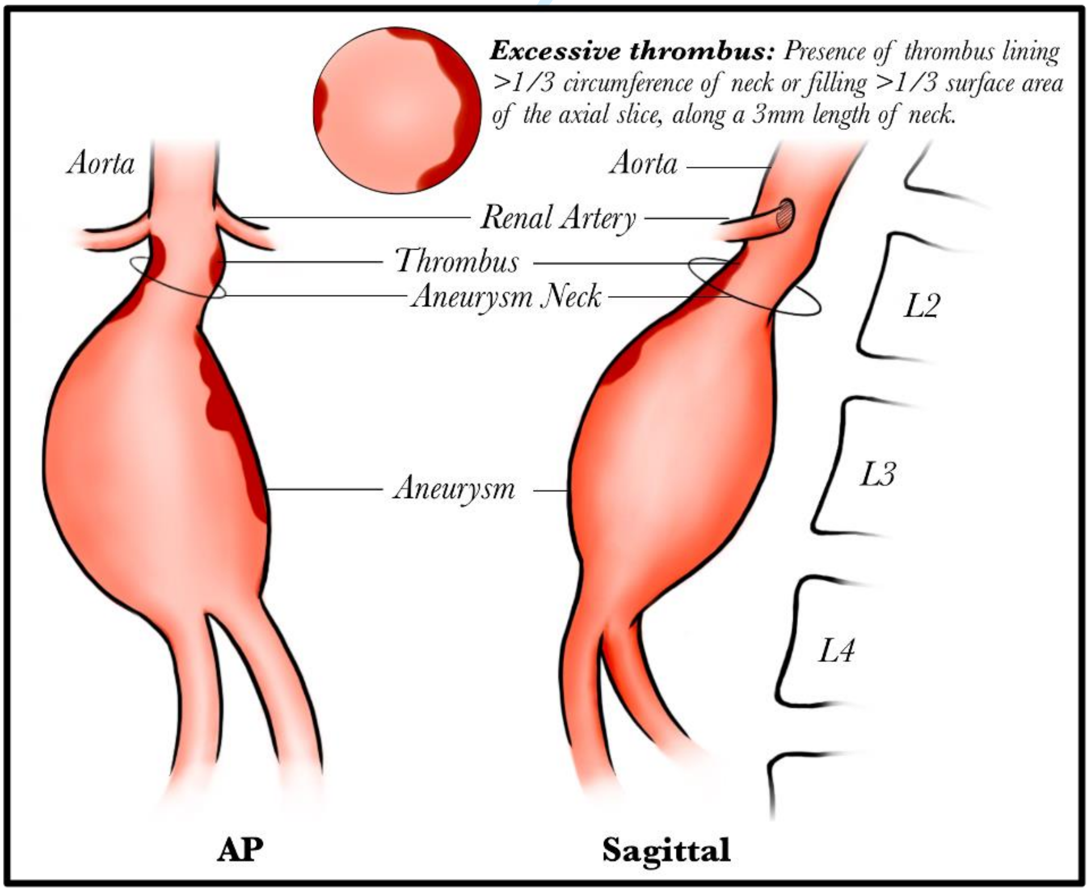

6. Neck diameters

Neck diameter is defined as the maximal outer to outer diameter (mm) in orthogonal cross section. The aneurysm neck is profiled in both coronal and sagittal views with subsequent assessment in the orthogonal axial view. Neck diameters are measured at the level of the lowermost renal artery (Figure 6a), at 5mm below this point (Figure 6b), at 10mm below this point (Figure 6c), and at 15mm below this point (Figure 6d). 15mm of neck length was chosen for diameter assessment as it is the most common length of seal zone stipulated in an EVAR stent graft's instructions for use document. The various positions along the length of the neck will be defined using the ruler function on the coronal view as seen in Figures 6a to 6e. Where the neck is <15mm in length, a neck diameter measurement will additionally be taken at the most distal point of the neck.

6a

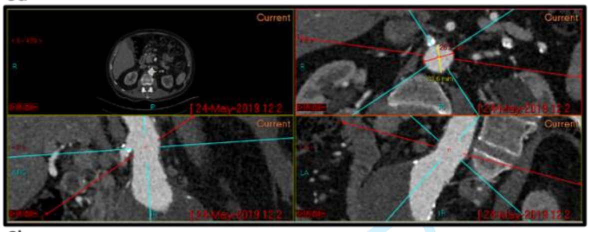

6c

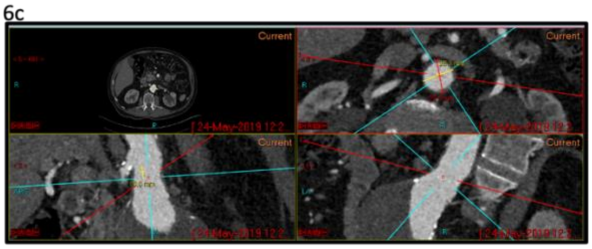

6b

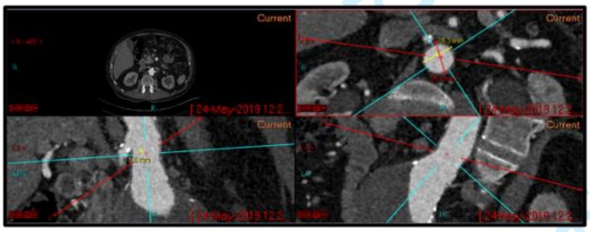

6d

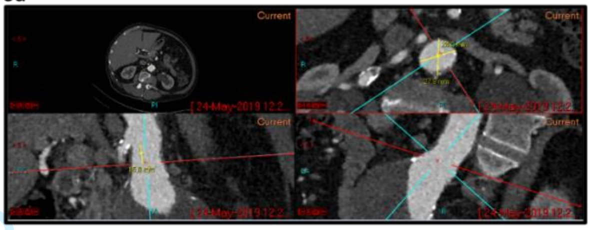

6e

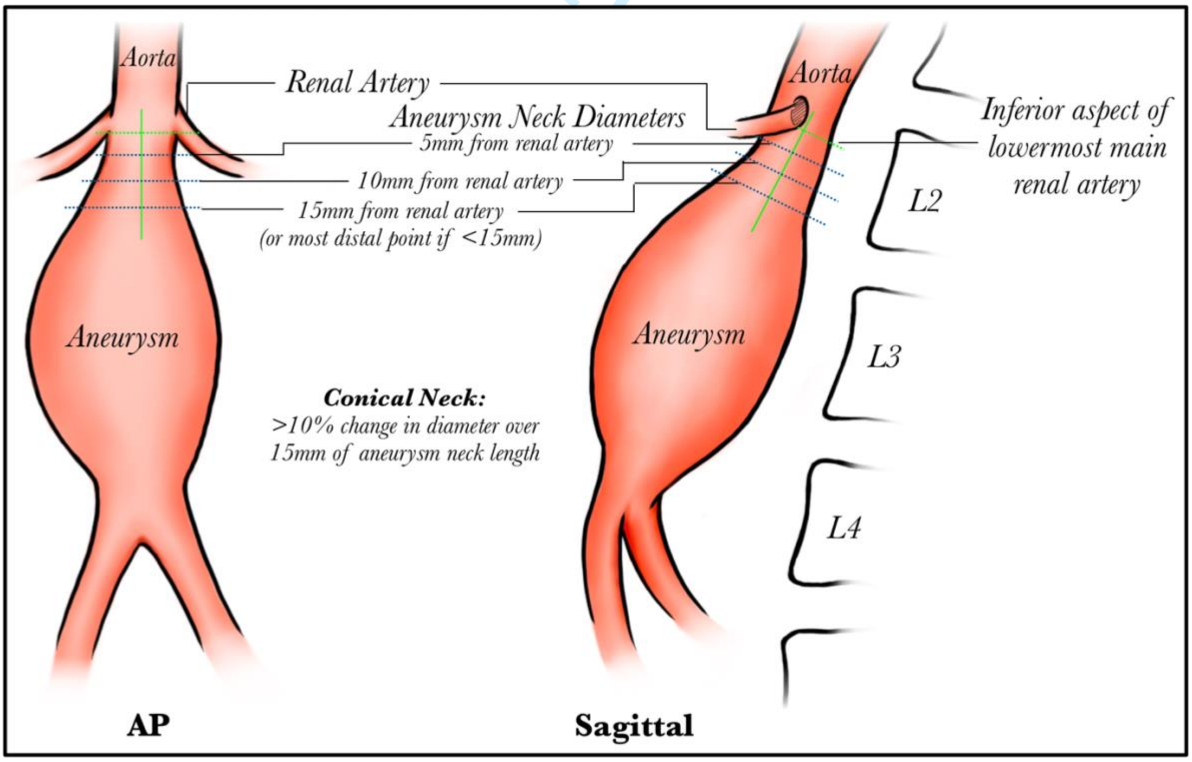

7. *<u>α neck angle</u>*

α neck angle refers to the angle, in degrees, between the axis of the suprarenal aorta and the axis of the infrarenal neck.

The first step of measurement is alignment of the axis of the neck in both coronal and sagittal planes. Tilting the double-oblique marker lines in the axial view in the next step will align the aorta in the coronal or sagittal views with the neck visible in the same field. The angle function is used to measure the angle between the neck and the suprarenal aorta and α neck angle is calculated by subtraction from 180 (Figures 7a and 7b).

7a

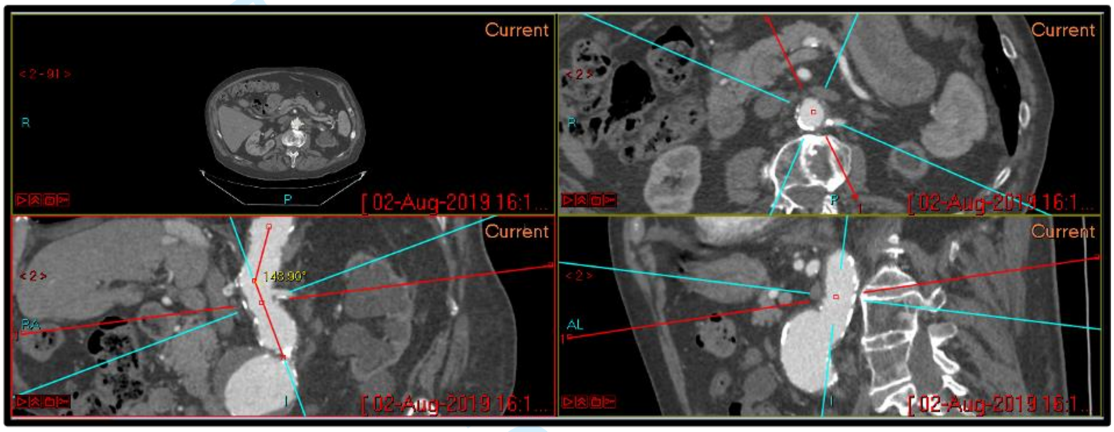

7b

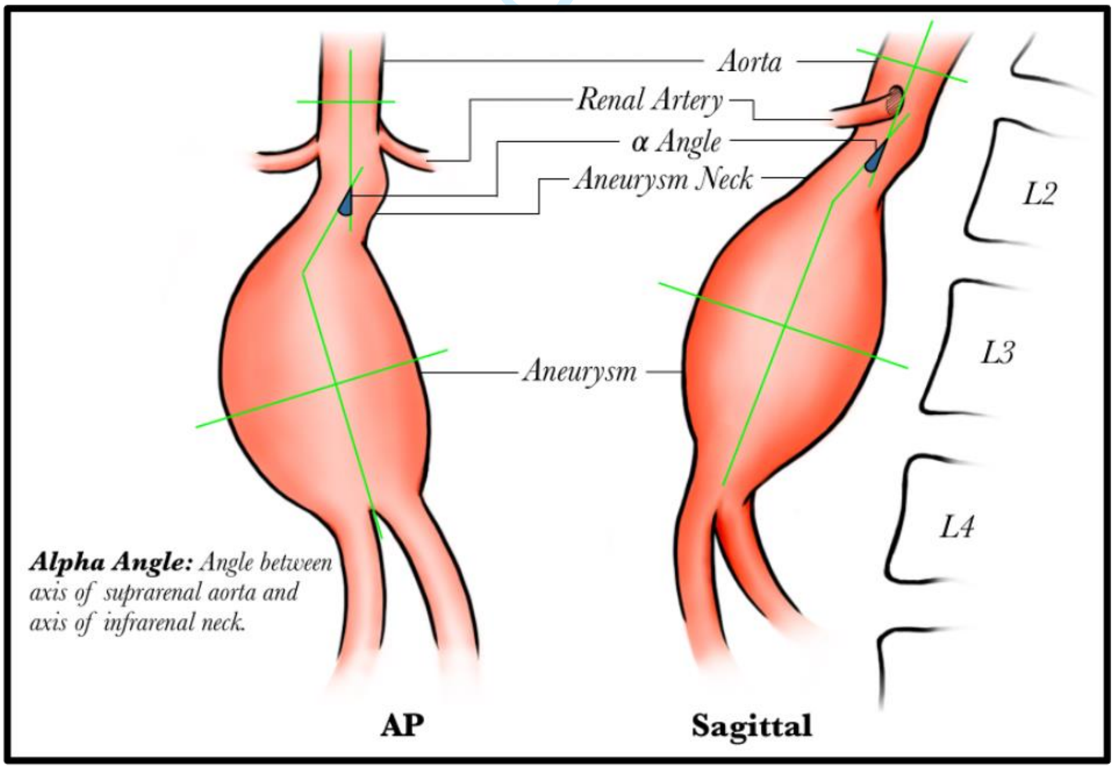

8. Non-straight necks

Non-straight neck refers to the presence of intra-neck angulation >60 degrees. This is recorded as binary outcome. If present, then a further “juxtarenal neck length” is measured, referring to the length of aneurysm neck proximal to the point of angulation but below the level of the lowermost major renal artery. This is demonstrated in Figures 8a and 8b.

Measurement of intra-neck angulation utilises the same tools and techniques as described for the aforementioned α and β neck angle measurements (Figures 3 and 7). Measurement of juxtarenal neck length utilises the same tools and techniques as described for the aforementioned total neck length measurement (Figure 2).

8a

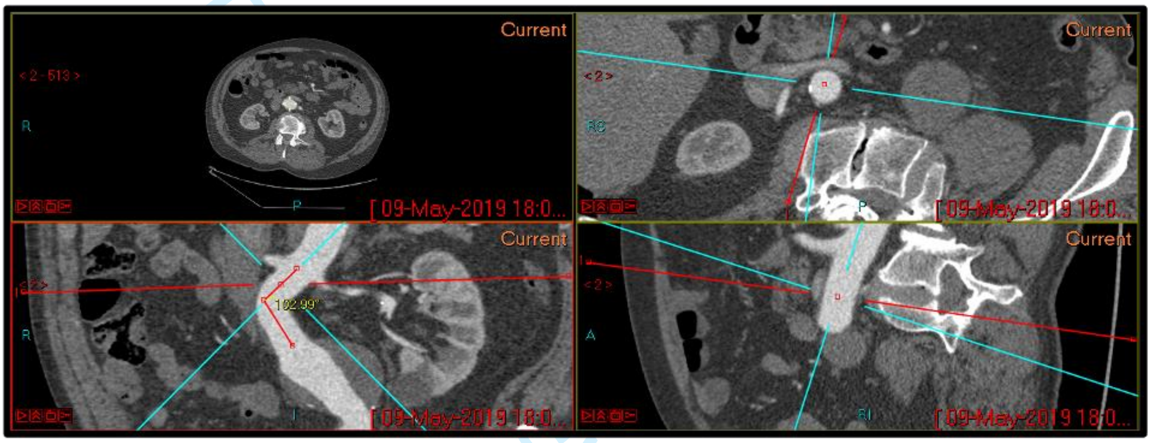

8b

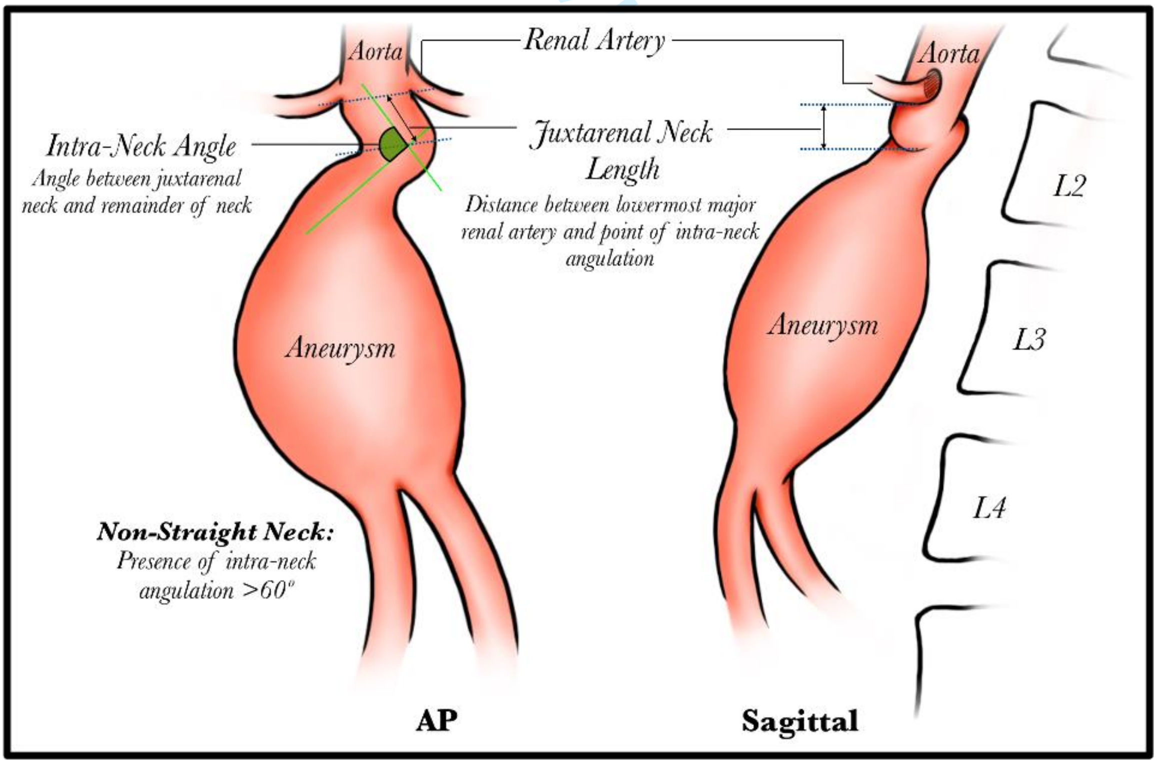

9. *Estimated Aortic Clamp Level*

This refers to the most likely position of an aortic cross clamp in the event of the aneurysm being repaired by open surgery. The levels are:

- **Infra-renal** (below the lowermost major renal artery)- if total neck length ≥5mm and free from excessive thrombus.
- **Inter-renal** (between the left and right renal arteries) - if total neck length <5mm and there is an aortic length of ≥5mm between the renal arteries.
- **Supra-renal** (between the uppermost major renal artery and the superior mesenteric artery) - if total neck length <5mm and there is no adequate inter-renal space and there is distance between uppermost renal artery and SMA ≥5mm.
- **Supra-coeliac** (above the coeliac axis) - if none of the aforementioned clamp positions are possible.

Estimating clamp level prediction requires 3 separate measurements:

1) Total neck length - this has been previously described.

2) Inter-vessel separation for renal arteries.
   This can be measured by profiling the main renal arteries on either side in the coronal view and using the ruler function to measure the distance between the two renal arteries.

3) Inter-vessel separation distance between uppermost main renal artery and SMA.
   This can be measured by profiling the renal arteries in the coronal view, marking the position of the pointer at that level so that the renal artery position can be correlated on the sagittal view. In the sagittal view, the SMA can be profiled enabling measurement of distance between the 2 with the ruler function.

For peer review only

Section 4 - The UK-COMPASS Corelab protocol – internal validation of the measurement process.

An internal validation study was performed on a subset of 70 CT scans, randomly selected from a pool of the first 500 CT scans received in the Corelab. 3 assessors of varying seniority (medical student, junior vascular trainee and senior vascular trainee) undertook independent and blinded assessment of the CT scans using the Corelab Protocol measurement process described in this manuscript. Seven measurements undergoing validity testing included maximal aneurysm diameter, total neck length, beta angle and neck diameters at 4 points along a 15mm length. Additionally, the results of the two trainees (of a seniority level reasonably expected to perform the Corelab analysis) underwent analysis to investigate potential discrepancies of patient grouping (by neck length) as a result of the measurement process.

Comparisons between Rater 1 vs Rater 2, Rater 3 vs Rater 1 and Rater 3 vs Rater 2 were performed in order to calculate and summarise the inter-rater variability. A two-way mixed effects model (each measurement done by the same set of raters, who are the only possible evaluators) was used to estimate the intra-class correlations (ICC) along with 95% confidence intervals for all 7 different measurements. Furthermore, Bland-Altman plots were generated. All statistical analysis was performed using STATAv15.1 (StataCorp. 2017. Stata Statistical Software: Release 15. College Station, TX: StataCorp LLC) An alpha level of 5% was assumed.

Inter-rater variability for the 3 raters across the 7 different measurement domains is demonstrated:

Statistic	Total neck length (units)	β Neck angle	Aneurysm diameter (units)	Diameter 0 (units)	Diameter 5 (units)	Diameter 10 (units)	Diameter 15 (units)
n	210	190	210	190	184	180	164
Mean	1.03	-0.38	0.56	0.03	0.05	0.10	-0.11
SD	6.65	10.14	2.47	1.10	1.27	1.18	2.43
Minimum	-18.0	-51.0	-4.0	-3.0	-6.0	-4.0	-19.0
Maximum	31.0	19.0	15.0	5.0	5.0	4.0	3.0

For peer review only

There was minimal inter-rater variability for all 7 domains as shown in these Bland-Altman plots:

Bland Altmann plots demonstrating excellent inter-rater agreement for measuring (a) neck length, (b) beta angle, (c) aneurysm diameter, (d) diameter of neck at level of renal artery, (e) diameter of neck 5mm below renal artery, (f) diameter of neck 10mm below renal artery, (g) diameter of neck 15mm below renal artery.
To generate the scatter plots below the mean value across the three observers (for each anatomical parameter) was calculated for each scan (x-axis). The average difference was calculated for each measurement for all three observers (y-axis).

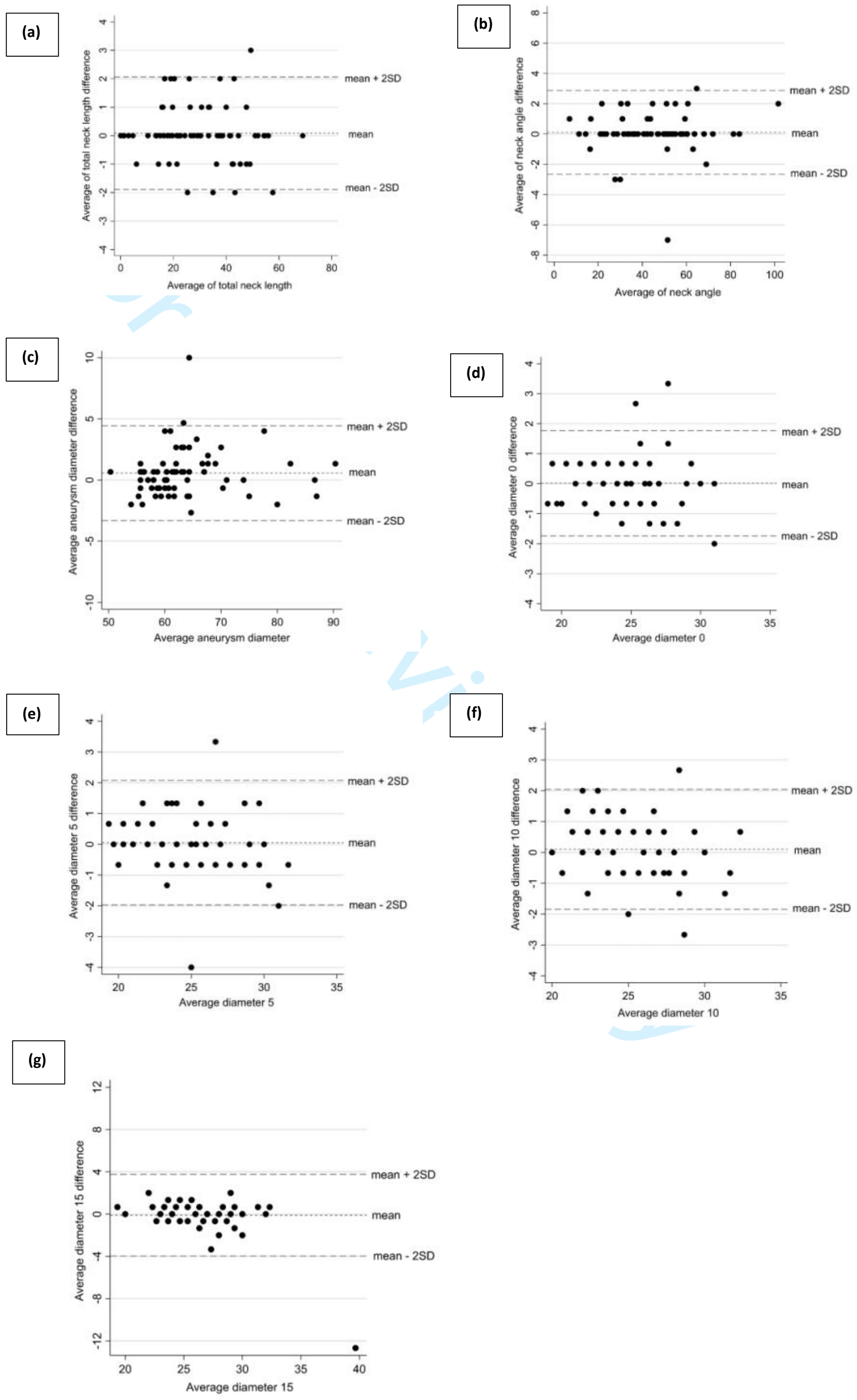

Inter-class correlation is demonstrated:

Statistic	Total neck length (units)	β Neck angle	Aneurysm diameter (units)	Diameter 0 (units)	Diameter 5 (units)	Diameter 10 (units)	Diameter 15 (units)
ICC	0.972	0.952	0.983	0.976	0.971	0.974	0.909
95% CI	0.959 – 0.982	0.927 – 0.969	0.974 – 0.989	0.964 – 0.985	0.956 – 0.982	0.960 – 0.983	0.857 – 0.945
F (df1, df2)	F(69, 138)=36.76	F(62, 124)=20.60	F(69, 138)=62.32	F(62, 124)=41.22	F(60, 120)=34.36	F(59, 118)=38.09	F(52, 104)=10.89
p-value	*<0.001*	*<0.001*	*<0.001*	*<0.001*	*<0.001*	*<0.001*	*<0.001*

A high degree of reliability was found between all measurements in all 7 domains across all 3 raters (ICC >90% for all 7 measures; p<0.001 for all).

For the grouping validation exercise of the 2 trainee measurements, a kappa statistic was calculated in order to examine inter-rater reliability. The distribution of grouping numbers for the two raters is shown:

		Rater 1				
		**1**	**2**	**3**	**4**	**Total**
**Rater 2**	**1**	*8*	*0*	*0*	*0*	*8*
	**2**	*0*	*2*	*0*	*0*	*2*
	**3**	*0*	*0*	*27*	*0*	*27*
	**4**	*0*	*0*	*0*	*33*	*33*
	**Total**	*8*	*2*	*27*	*33*	*70*

(Grp 1: length <5mm, Grp 2: length 4-9mm, Grp 3: length ≥10mm, Grp 4: not complex)

There was a perfect inter-rater agreement [k=1.00, se=0.0881], suggesting that any discrepancies in measurement did not correlate to discrepancies in subsequent grouping for analysis.

For peer review only - http://bmjopen.bmj.com/site/about/guidelines.xhtml

Section 5 - Microcosting exercise data collection template

Micro-costing Template for UK-COMPASS

Please complete the proforma below and provide quantities and further details in the free text boxes, in as much detail as possible e.g. brands/sizes/versions etc.

Date of procedure

1) Operation for juxtarenal aneurysm. Please tick one:

FEVAR	Open repair	Infrarenal EVAR	EVAR + Endoanchors	EVAR + Chimneys	Other (please specify)

2) Pre-operative care *(from day of diagnosis of complex aneurysm until the day of surgery)*

ITEM	Quantity	Further details
**Outpatient appt with operator**		
**Outpatient appt with other** *(Anaesthetics, another referral)*		
**Pre-assessment appt**		
**Blood tests**		
**Scans**		

For peer review only

**ECG**		
**Spirometry**		
**Transthoracic ECHO**		
**Dobutamine stress ECHO**		
**Exercise stress ECHO**		
**CPET**		
**Other tests (please specify)**		

For peer review only

3) Surgery *(from arrival to hospital until the day of discharge)*

- **Theatre utilisation**

ITEM	Quantity	Further details
**Hybrid suite (time in mins)**		
**Operating theatre (time in mins)**		

**IR suite (time in mins)**		

- **Personnel**

ITEM	Quantity	Further details
**Surgeon**		
**Radiologist**		
**Assistants**		
**Anaesthetist**		
**Anaesthetist Juniors**		
**ODP**		
**Scrub nurse**		
**Other theatre staff (please specify)**		

- **Medications**

ITEM	Quantity	Further details

For peer review only

**Anaesthetic Drugs**		
**Antibiotics**		
**Heparin**		
**Other intra-operative meds (please specify)**		

- **Standard Equipment**

ITEM	Quantity	Further details
**Anaesthetic equipment (endotracheal tube, cannulas, other lines etc.)**		
**Theatre trays**		
**Skin prep**		
**Drapes**		

For peer review only

**Other general theatre equipment (please specify)**		

- **Specialist Equipment**

ITEM	Quantity	Further details
**USS machine for puncture**		
**Puncture kit with standard J wire x2 (for both sides)**		
**Initial Sheaths x2 (with puncture, both sides)**		
**Large Sheaths (for device delivery and cannulation, both sides)**		
**Pigtail catheter**		
**Stiff guidewire x2 (for both sides)**		
**PROXIMAL MAIN BODY (fenestrated piece if FEVAR)**		
**Catheters for target vessels (if appropriate)**		

For peer review only

**Guidewires for target vessels (if appropriate)**		
**Sheaths for target vessels (if appropriate)**		
**Target vessel stents (for FEVAR and Chimney cases)**		
**DISTAL MAIN BODY (for FEVAR)**		
**CONTRALATERAL LIMB + ADDITIONAL LIMBS ON EITHER SIDE**		
**Coda Balloon**		
**Contrast Agent**		
**Other specialist equipment (please specify)**		

For peer review only

4) Post-operative care *(from day 1 post-op to discharge from service/death)*

- **Inpatient stay**

ITEM	Quantity	Further details
**Length of stay in bed on ward (level 1)**		

For peer review only - http://bmjopen.bmj.com/site/about/guidelines.xhtml

**Length of stay in Level 2 bed**		
**Length of stay in Level 3 bed**		
**Nursing care**		
**Physiotherapy care**		
**OT care**		
**Intermediate care if applicable**		

- **Outpatient care**

ITEM	Quantity	Further details
**Follow-up clinic appointments**		
**Scans**		
**Community Nursing input**		
**GP visits**		

For peer review only

STROBE Statement—Checklist of items that should be included in reports of ***cohort studies***

	Item No	Recommendation
**Title and abstract**	1	(*a*) Indicate the study's design with a commonly used term in the title or the abstract<br>As per title, we have referred to "cohort comparison study".
		(*b*) Provide in the abstract an informative and balanced summary of what was done and what was found<br>Page 2: Methods and Analysis section in the abstract details the design ("what was done").<br>"what was found" is not appropriate for a protocol.
**Introduction**		
Background/rationale	2	Explain the scientific background and rationale for the investigation being reported<br>Pages 4 and 5: This is provided throughout the introduction.
Objectives	3	State specific objectives, including any prespecified hypotheses<br>Page 5: This is provided in the final paragraph of the introduction, and in Table 1.
**Methods**		
Study design	4	Present key elements of study design early in the paper<br>Page 6 and 7: This is presented in the first subsection of the Methods and Analysis section, See: "Overview".
Setting	5	Describe the setting, locations, and relevant dates, including periods of recruitment, exposure, follow-up, and data collection<br>Page 7 and 8: Routinely collected data stream (clinical outcomes) and Site-reported data stream (quality of life) are described in detail separately in the Methods Section.
Participants	6	(*a*) Give the eligibility criteria, and the sources and methods of selection of participants. Describe methods of follow-up<br>Table 2: eligibility criteria for Routinely collected data stream<br>Page 8: eligibility criteria for Site-reported data stream<br>Pages 7 and 8: information regarding sources of patients/data and follow-up for both study streams.
		(*b*) For matched studies, give matching criteria and number of exposed and unexposed<br>n/a
Variables	7	Clearly define all outcomes, exposures, predictors, potential confounders, and effect modifiers. Give diagnostic criteria, if applicable<br>Top of page 7: Primary and secondary outcome measures clearly defined.<br>Page 9: "Data analysis plan" section provides details on the confounders that are being adjusted.<br>Supplementary material file: Section 2 on Page 4 provides definitions of neck parameters
Data sources/ measurement	8*	For each variable of interest, give sources of data and details of methods of assessment (measurement). Describe comparability of assessment methods if there is more than one group<br>Pages 7 and 8: information regarding sources of patients/data.<br>Pages 9 and 10: methods of assessment detailed in the "Data Analysis Plan" section.
Bias	9	Describe any efforts to address potential sources of bias<br>Pages 9 and 10: "Data Analysis Plan" section details how variation in anatomy and physiological risk will be addressed – using propensity scoring and group stratification techniques
Study size	10	Explain how the study size was arrived at<br>Bottom of page 8, and page 9: Sample Size calculations are provided

For peer review only

Quantitative variables	11	Explain how quantitative variables were handled in the analyses. If applicable, describe which groupings were chosen and why<br>Page 9 and 10: This is detailed in the Data Analysis Plan section
Statistical methods	12	(*a*) Describe all statistical methods, including those used to control for confounding<br>Page 9 and 10: This is detailed in the Data Analysis Plan section
		(*b*) Describe any methods used to examine subgroups and interactions<br>Page 9 and 10: This is detailed in the Data Analysis Plan section – subgroups are based on aortic neck length.
		(*c*) Explain how missing data were addressed<br>Page 10 and 11: "It is proposed that covariates with small amounts of missing data (≤10%) will be included on a complete case basis and covariates with a large amount of missing data (>10%) will be excluded from the analysis."
		(*d*) If applicable, explain how loss to follow-up was addressed<br>Only the site-reported data/prospective aspect to the trial will be subject to loss of follow-up. There is an expected loss to follow-up rate of 50% through noncompliance and natural attrition from death during the follow-up period. This was taken into consideration with sample size calculation as explained on page 10 of the mnuscript
		(*e*) Describe any sensitivity analyses<br>Page 11: "Sensitivity analyses are carried out to ensure the results are not sensitive to either the propensity model or the method of matching. With respect to the propensity model, sensitivity analyses shall be carried out by removing each covariate included in calculating the propensity score and repeating the analysis of the primary endpoints. The results of all sensitivity analyses shall be presented in a forest plot along with the overall effect from the primary analysis."
**Results: this whole section cannot be completed as this is a protocol publication and no results are presented.**		
Participants	13*	(a) Report numbers of individuals at each stage of study—eg numbers potentially eligible, examined for eligibility, confirmed eligible, included in the study, completing follow-up, and analysed
		(b) Give reasons for non-participation at each stage
		(c) Consider use of a flow diagram
Descriptive data	14*	(a) Give characteristics of study participants (eg demographic, clinical, social) and information on exposures and potential confounders
		(b) Indicate number of participants with missing data for each variable of interest
		(c) Summarise follow-up time (eg, average and total amount)
Outcome data	15*	Report numbers of outcome events or summary measures over time
Main results	16	(*a*) Give unadjusted estimates and, if applicable, confounder-adjusted estimates and their precision (eg, 95% confidence interval). Make clear which confounders were adjusted for and why they were included
		(*b*) Report category boundaries when continuous variables were categorized
		(*c*) If relevant, consider translating estimates of relative risk into absolute risk for a meaningful time period
Other analyses	17	Report other analyses done—eg analyses of subgroups and interactions, and sensitivity analyses
**Discussion this whole section cannot be completed as this is a protocol publication.**		
Key results	18	Summarise key results with reference to study objectives
Limitations	19	Discuss limitations of the study, taking into account sources of potential bias or imprecision. Discuss both direction and magnitude of any potential bias
Interpretation	20	Give a cautious overall interpretation of results considering objectives, limitations,

For peer review only - http://bmjopen.bmj.com/site/about/guidelines.xhtml

		multiplicity of analyses, results from similar studies, and other relevant evidence
Generalisability	21	Discuss the generalisability (external validity) of the study results
**Other information**		
Funding	22	Give the source of funding and the role of the funders for the present study and, if applicable, for the original study on which the present article is based

*Give information separately for exposed and unexposed groups.

Note: An Explanation and Elaboration article discusses each checklist item and gives methodological background and published examples of transparent reporting. The STROBE checklist is best used in conjunction with this article (freely available on the Web sites of PLoS Medicine at http://www.plosmedicine.org/, Annals of Internal Medicine at http://www.annals.org/, and Epidemiology at http://www.epidem.com/). Information on the STROBE Initiative is available at http://www.strobe-statement.org.

For peer review only
